# Development of 1-(4-(Substituted)piperazin-1-yl)-2-((2-((4-methoxybenzyl)thio)pyrimidin-4-yl)oxy)ethanones That Target Poly (ADP-Ribose) Polymerase in Human Breast Cancer Cells

**DOI:** 10.3390/molecules27092848

**Published:** 2022-04-29

**Authors:** Suresha N. Deveshegowda, Prashant K. Metri, Rashmi Shivakumar, Ji-Rui Yang, Shobith Rangappa, Ananda Swamynayaka, Muthu K. Shanmugam, Omantheswara Nagaraja, Mahendra Madegowda, Priya Babu Shubha, Arunachalam Chinnathambi, Sulaiman Ali Alharbi, Vijay Pandey, Kwang Seok Ahn, Peter E. Lobie, Basappa Basappa

**Affiliations:** 1 Laboratory of Chemical Biology, Department of Studies in Organic Chemistry, University of Mysore, Manasagangotri, Mysore 570006, India; sureshand92@gmail.com (S.N.D.); prashant.metri@gmail.com (P.K.M.); rashmis1410@gmail.com (R.S.); 2 Tsinghua Berkeley Shenzhen Institute, Tsinghua Shenzhen International Graduate School, Tsinghua University, Shenzhen 518055, China; yang.jirui@sz.tsinghua.edu.cn (J.-R.Y.); vijay.pandey@sz.tsinghua.edu.cn (V.P.); 3 Adichunchanagiri Institute for Molecular Medicine, BG Nagara, Nagamangala Taluk, Mandya 571448, India; shobithrangappa@gmail.com; 4 Department of Studies in Physics, University of Mysore, Manasagangotri, Mysore 570006, India; anandas@physics.uni-mysore.ac.in (A.S.); omantheswara@physics.uni-mysore.ac.in (O.N.); mahendra@physics.uni-mysore.ac.in (M.M.); 5 Department of Pharmacology, Yong Loo Lin School of Medicine, National University of Singapore, Singapore 117600, Singapore; phcsmk@nus.edu.sg; 6 Department of Studies in Chemistry, University of Mysore, Manasagangotri, Mysore 570006, India; priyabs_chem@yahoo.com; 7 Department of Botany and Microbiology, College of Science, King Saud University, Riyadh 11451, Saudi Arabia; carunachalam@ksu.edu.sa (A.C.); sharbi@ksu.edu.sa (S.A.A.); 8 KHU-KIST Department of Converging Science and Technology, Kyung Hee University, Seoul 02447, Korea; ksahn@khu.ac.kr; 9 Department of Science in Korean Medicine, Kyung Hee University, 24 Kyungheedae-ro, Dongdaemun-gu, Seoul 02447, Korea; 10 Institute of Biopharmaceutical and Health Engineering, Tsinghua Shenzhen International Graduate School, Tsinghua University, Shenzhen 518055, China; 11 Shenzhen Bay Laboratory, Shenzhen 518055, China

**Keywords:** PARPi, breast cancer, thiouracil amides, apoptosis

## Abstract

A number of uracil amides cleave poly (ADP-ribose) polymerase and therefore novel thiouracil amide compounds were synthesized and screened for the loss of cell viability in a human-estrogen-receptor-positive breast cancer cell line. The synthesized compounds exhibited moderate to significant efficacy against human breast cancer cells, where the compound **5e** IC_50_ value was found to be 18 μM. Thouracil amide compounds **5a** and **5e** inhibited the catalytical activity of PARP1, enhanced cleavage of PARP1, enhanced phosphorylation of H2AX, and increased CASPASE 3/7 activity. Finally, in silico analysis demonstrated that compound **5e** interacted with PARP1. Hence, specific thiouracil amides may serve as new drug-seeds for the development of PARP inhibitors for use in oncology.

## 1. Introduction

Poly (ADP-ribose) polymerase (PARP) is an enzyme which catalyzes poly ADP-ribosylation of specific target proteins. The PARP family contains 17 enzymes, among which PARP1 is the most abundant and widely studied [1,2,3]. It is involved in a number of cellular functions, such as DNA repair, recombination, and gene transcription [4]. More than 70 PARP inhibitors are undergoing clinical trials, some of which are approved by the U.S. Food and Drug Administration, for instance, Niraparib, Olaparib, Talazoparib, and Rucaparib [5,6,7,8]. Evidence suggests that PARP plays an important role in cancer cell signaling, chromatin regulation, and metastatic processes that involve cell survival, migration, and invasion [9]. For example, in estrogen-receptor-positive (ER+) breast cancer, PARP regulates the transcriptional activity of ERα, and is reported to be downstream of ER-dependent transcriptional responses [10].

A number of PARP1 inhibitors (PARPi) have been reported; however, the commonly used example of a PARPi, 3-substituted benzamide (3-AB) was a nicotinamide analog [11]. Generally, benzamides inhibit PARP due to their structural analogy to NAD and therefore compete with NAD at the catalytic domain of PARPs [12]. Despite their ability to elucidate the function of PARP, benzamides fail to show PARP inhibitory activity in nanomolar ranges and exhibited off-target effects that precluded their clinical evaluation. Additionally, indazole amides have been shown to be PARP1 inhibitors [13].

Conversely, substituted uracil derivatives have been reported as potent inhibitors of PARP by inhibiting thymidylate synthesis as well as basal excision repair (BER) mechanisms [14,15]. Indeed, thiouracil- or uracil-based compounds are known to act on cancer cells via an anti-folate mechanism, where its misincorporation is genotoxic and eventually contributes to DNA double-strand break and ultimately cell death [16].

The synthesis, characterization, and efficacy of new thiouracil amides were therefore investigated herein and found a lead compound **5e** that inhibits both PARP1 catalytic activity and breast cancer cell viability.

## 2. Results and Discussion

### 2.1. Chemical Synthesis of Novel Thiouracil Amides

**Scheme 1:**
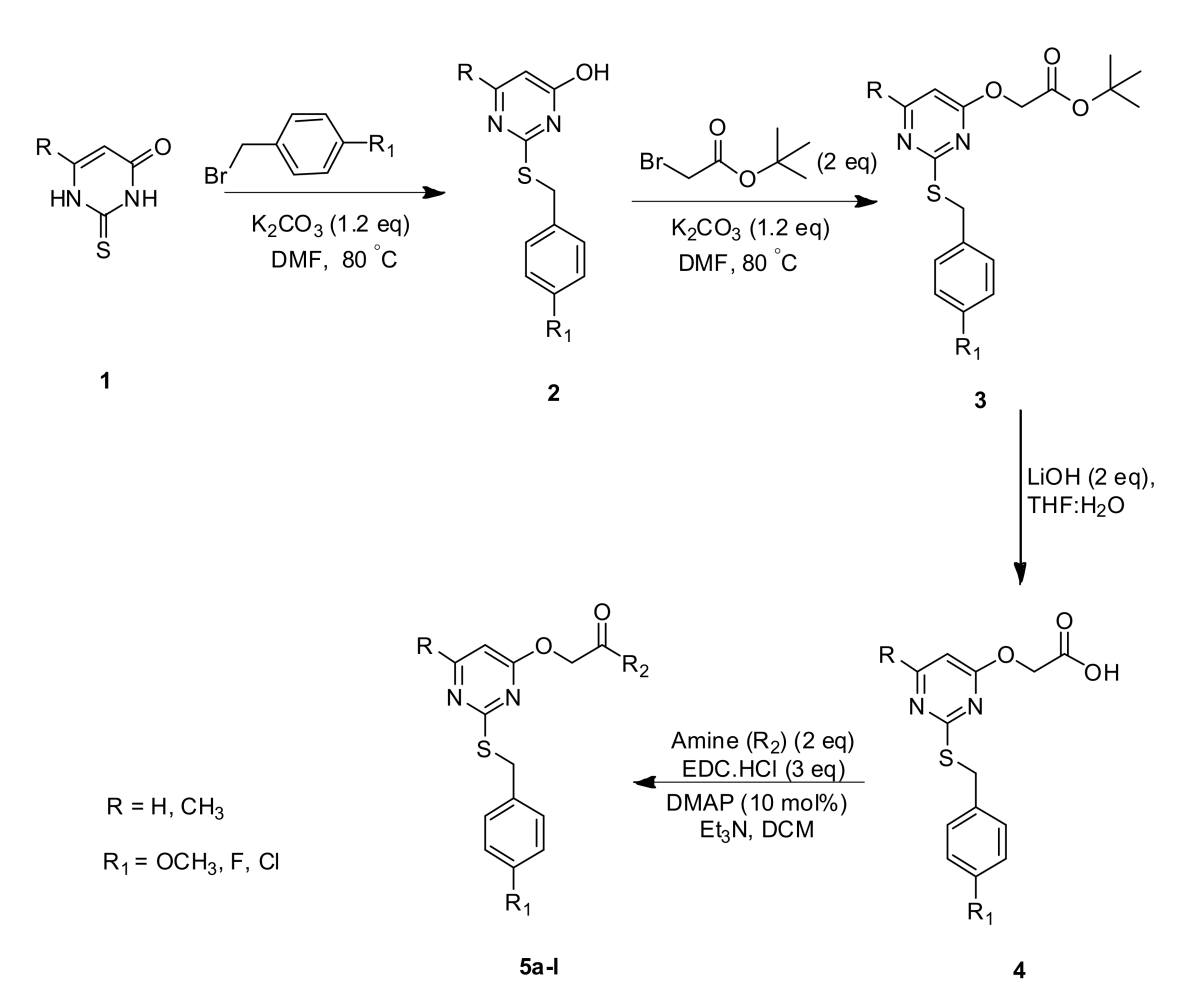
Synthesis of thiouracil containing amide derivatives.

The synthesis of novel thiouracil derivatives and their characterization is herein reported. In the present investigation, regioisomeric 2-thiopyrimidine acetic acids were utilized as a template to prepare a library of novel amides with various amines, as described in Scheme 1.

The synthetic sequence commenced by reacting 2-thiouracil with substituted benzyl derivative to afford S-benzylated products. The phenolic hydroxyl group in S-benzylated products was treated with tert-butyl acetate to afford tert-butyl esters which were subjected to hydrolysis to afford 2-thiopyrimidine acetic acid. The acid so obtained was coupled with various amines to afford amides **5a**–**5l**. The structures of these newly synthesized amide derivatives were confirmed by ^1^H NMR, ^13^CNMR, and LC/MS analysis.

### 2.2. Novel Thiouracil Derivatives Produce Loss of Breast Cancer Cell Viability

**Figure 1:**
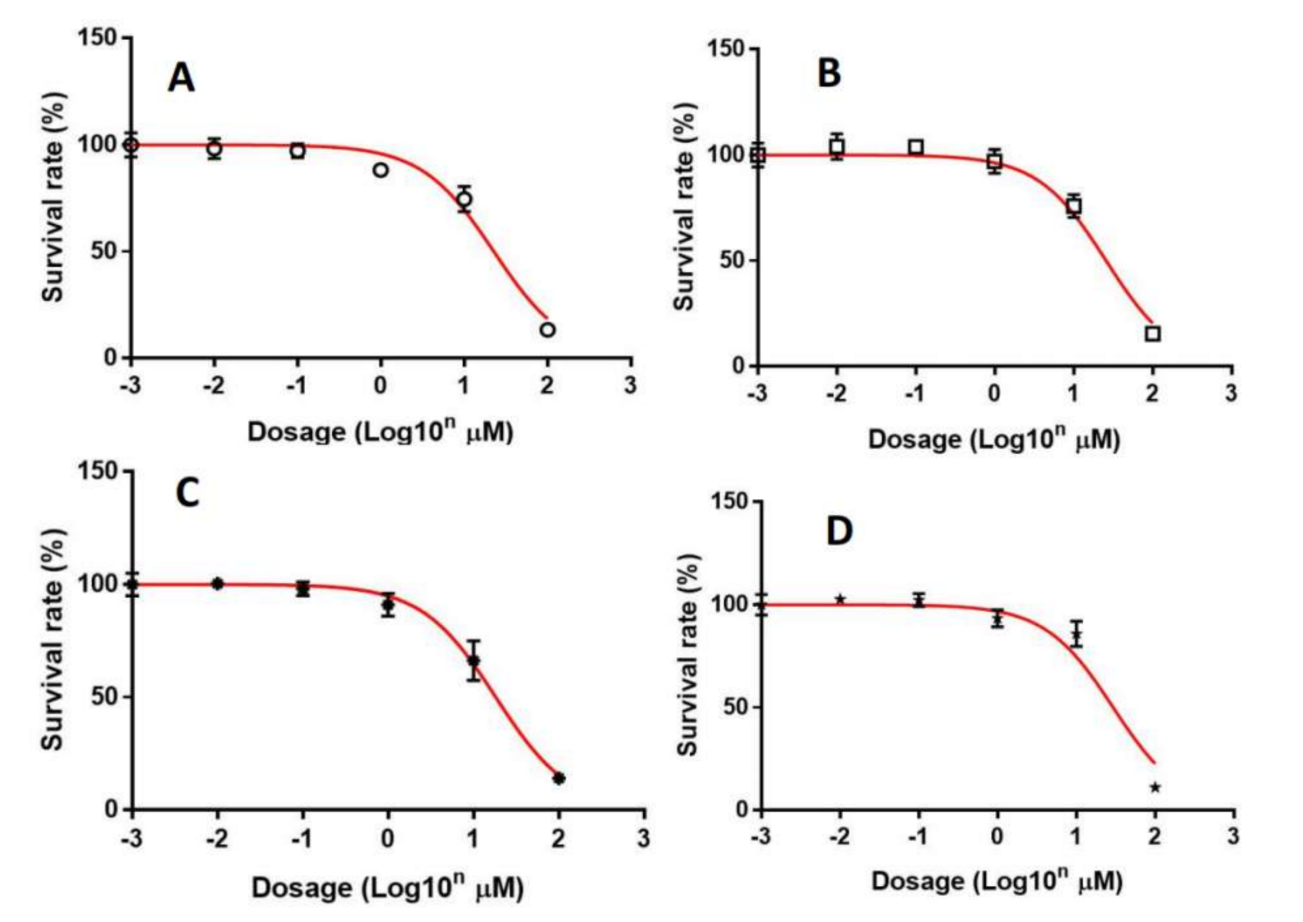
Loss of MCF-7 cell viability produced by compounds **5a**, **5b**, **5e**, and **5f**. MCF-7 were exposed to **5a** (**A**), **5b** (**B**), **5e** (**C**), and **5f** (**D**) for 72 h and the viability of cells was analyzed by Alamar Blue assays. The results are presented as mean ± S.E.M. of triplicate determinations.

The synthesized compounds were screened for loss of cell viability using the ER+ breast cancer cell line by Alamar Blue cell viability assays [17,18,19,20,21,22]. The compounds were dissolved in DMSO and cells were treated in a dose-dependent manner for 72 h. Amongst the tested amides, compounds **5a**, **5b**, **5e**, and **5f** showed significant loss of cell viability with IC_50_ values of 22.68, 25.71, 18.23, and 29.34 µM, respectively, as shown in Figure 1 and Table 1. The other compounds tested from the series exhibited less efficacy. The thiouracil amides possessing tolyl or halophenyl groups displayed significant loss of breast cancer cell viability, according to studies on the structure–activity relationship of this library of compounds. Furthermore, the loss of cell viability produced by the lead compounds (**5a**, **5e**) was compared with Olaparib using immortalized but otherwise normal human mammary epithelial cells (MCF-10A) as an indicator of effect on normal cells. It was observed that these compounds produced loss of cell viability of MCF-10A cells with IC_50_ values of 46.9 and 52.3 µM for compounds **5a** and **5e**, respectively, which were comparable to Olaparib, where IC_50_ was observed to be 57.3 µM (Supplementary Materials).

### 2.3. Compounds **5a** and **5e** Inhibit PARP1 Activity

**Figure 2:**
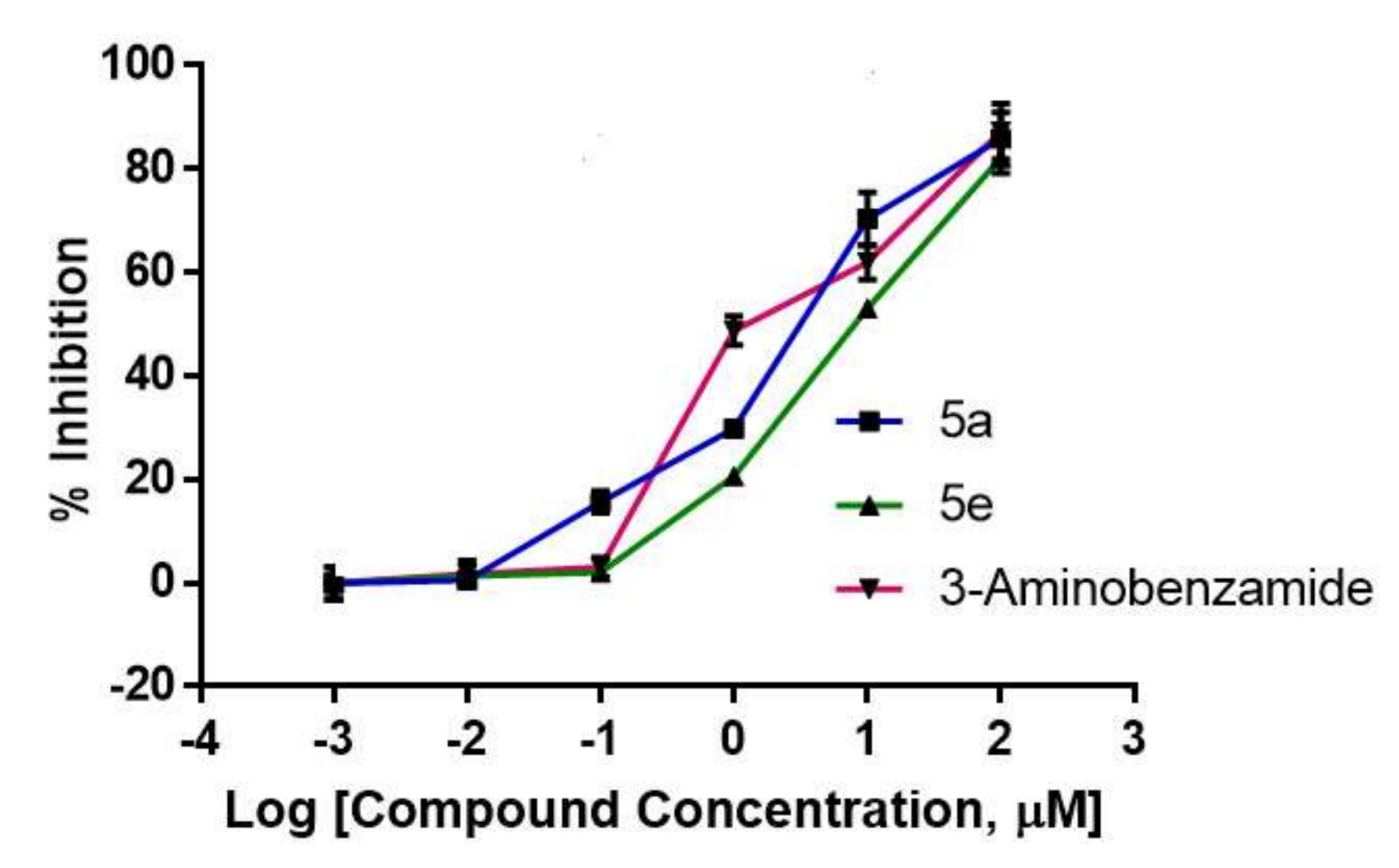
Compounds **5a**, **5e**, and 3-aminobenzamide inhibited the PARP1 catalytic activity. Graphical representation of the inhibition of the catalytic activity of PARP1 by indicated compounds (*n* = 3). Data are expressed as mean ± SEM.

Compounds **5a** and **5e** were tested for their capacity to inhibit PARP1 catalytic activity [23]. Compounds **5a** and **5e** were used at concentrations of 0.01, 0.1, 1, 10, and 100 µM. Compound **5a** was found to inhibit the catalytical activity of PARP1 0.5, 15.6, 29.8, 70.3, and 85.8% at the tested concentrations, whereas compound **5e** inhibited PARP1 activity at 1.3, 2.0, 20.6, 53, and 82.1%. 3-aminobenzamide inhibited PARP1 activity by 1.7, 3, 48.7, 61.9, and 87.2% at the tested concentrations, respectively (Figure 2).

### 2.4. Lead Compounds Increased PARP1 Cleavage, Phospho-H2AX Levels, and Caspase-3/7 Activation in MCF-7 Cells

**Figure 3:**
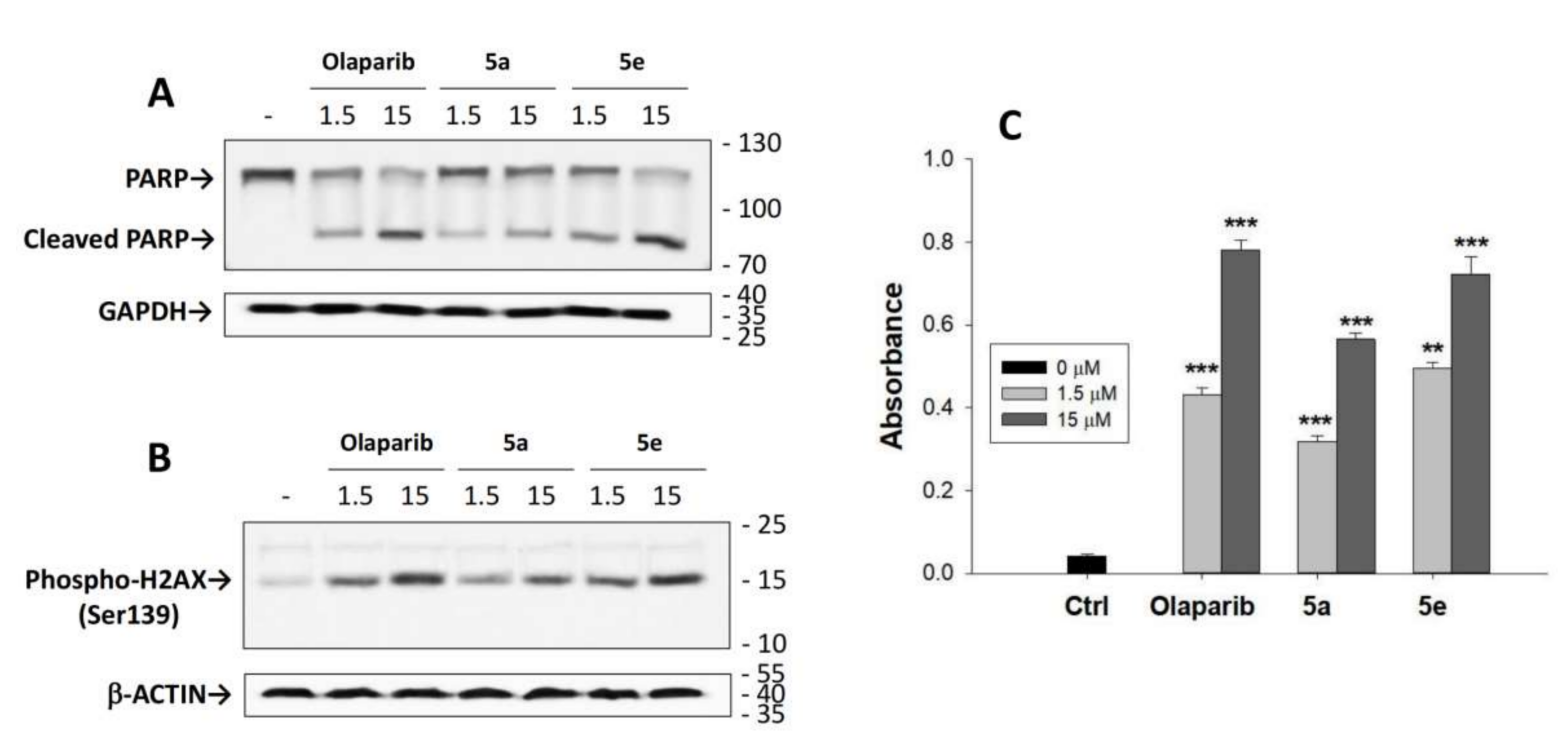
Compounds **5a**, **5e**, and Olaparib cleave PARP1 (**A**) and increase phosphorylation of H2A-X (**B**) and CASPASE-3/7 activity (**C**) in MCF-7 cells. MCF-7 cells were treated with or without compounds **5a, 5e**, or Olaparib at the indicated concentrations. The respective protein bands were detected by Western blot analysis. GAPDH or β-ACTIN were used as input controls. Data are expressed as mean ± SEM. ** *p* ≤ 0.01; *** *p* ≤ 0.001.

The effects of compounds **5a**, **5e,** and Olaparib on the cleavage of PARP1 in MCF-7 cells were performed using the previously reported protocol [24,25,26,27]. MCF-7 cells were incubated with **5a, 5e,** or Olaparib at the indicated concentrations. As observed in Figure 3A, treatment of breast cancer cells with either compound **5a** or **5e** produce PARP cleavage comparable to that observed with Olaparib.

Phosphorylation of Ser139 in H2AX is an indicator of cellular DNA damage which occurs upon PARP inhibition [28]. For example, Olaparib has been reported to enhance H2AX phosphorylation in various cancer cell lines [29]. Therefore, the effect of compounds **5a** or **5e** or Olaparib on the level of p-H2AX in MCF-7 cells was determined (Figure 3B). It was observed that compounds **5a** and **5e** increased phosphorylation of H2AX in MCF-7 cells comparable to that observed with Olaparib.

The effect of compounds **5a** or **5e** and Olaparib on CASPASE 3/7 activity was also examined as an indicator of apoptosis [30,31,32]. Hence, CASPASE 3/7 activity was determined after treatment of breast cancer cells with compounds **5a** or **5e** or Olaparib. Both compounds **5a** and **5e** increased CASPASE 3/7 activity in a dose-dependent manner and were roughly comparable to that observed with Olaparib (Figure 3C).

### 2.5. Density Functional Theory (DFT) Calculations

**Figure 4:**
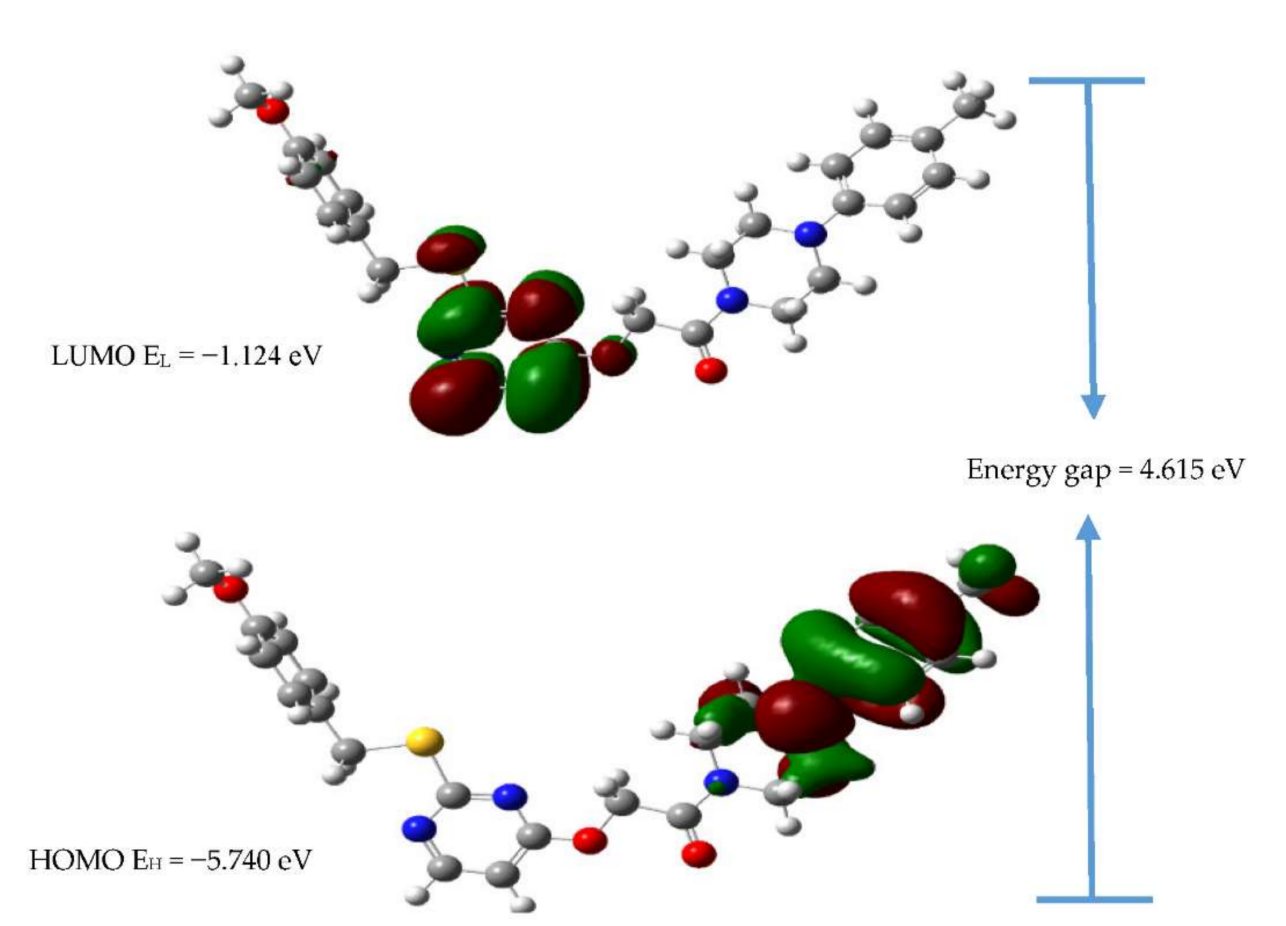
The HOMO and LOMO of molecule **5e** obtained by the DFT/B3LYP/6-31+G (d,p) method.

DFT calculations were performed to understand the stability of compound **5e** in the catalytical grove of PARP1 [33,34,35]. According to the frontier molecular orbital theory, the highest occupied molecular orbitals (HOMO) and lowest unoccupied molecular orbitals (LUMO) are the most effective factors in bioactivity. HOMO has the priority to provide electrons, while LUMO can accept electrons. Thus, studies on the frontier orbital energy may provide useful information of the biological mechanism [36]. The B3LYP level of theory was used to calculate the energy of HOMO, LUMO, and the E_gap_ value, which were used to study the chemical stability, molecular polarizability, and chemical softness or hardness of compound **5e**. The distribution and energy levels of the HOMO and LUMO for **5e** are shown in Figure 4. The positive phase of the molecule is represented in red color, whereas the negative phase is in green color. It is clear from the plots that the HOMO is localized on the 1-(4-methylphenyl)piperazine ring. In contrast, LUMO is delocalized on the methoxypyrimidine ring and sulfur atom. The HOMO and LUMO values of conformer **5e** are −5.740 eV and −1.124 eV, respectively, and the energy separation between HOMO and LUMO is 4.615 eV. The stabilization and interactions of the compounds with the catalytic site of the enzyme may be explained by global reactivity descriptors and are represented in Table 2. The ionization energy can be expressed as I = −E_HOMO_ = 5.740 eV, and the electron affinity can be expressed as A = −E_LUMO_ = 1.124 eV. Hardness represents the resistance of a chemical system against the deformation of the electron cloud under the slight interference encountered in the chemical process and can be expressed as η = (E_LUMO_ − E_HOMO_)/2. The hardness and softness values of the **5e** molecule are 2.307 eV and 0.216 e^−1^ V^−1^, respectively. With the smaller value of softness, the molecule is chemically stable.

**Figure 5:**
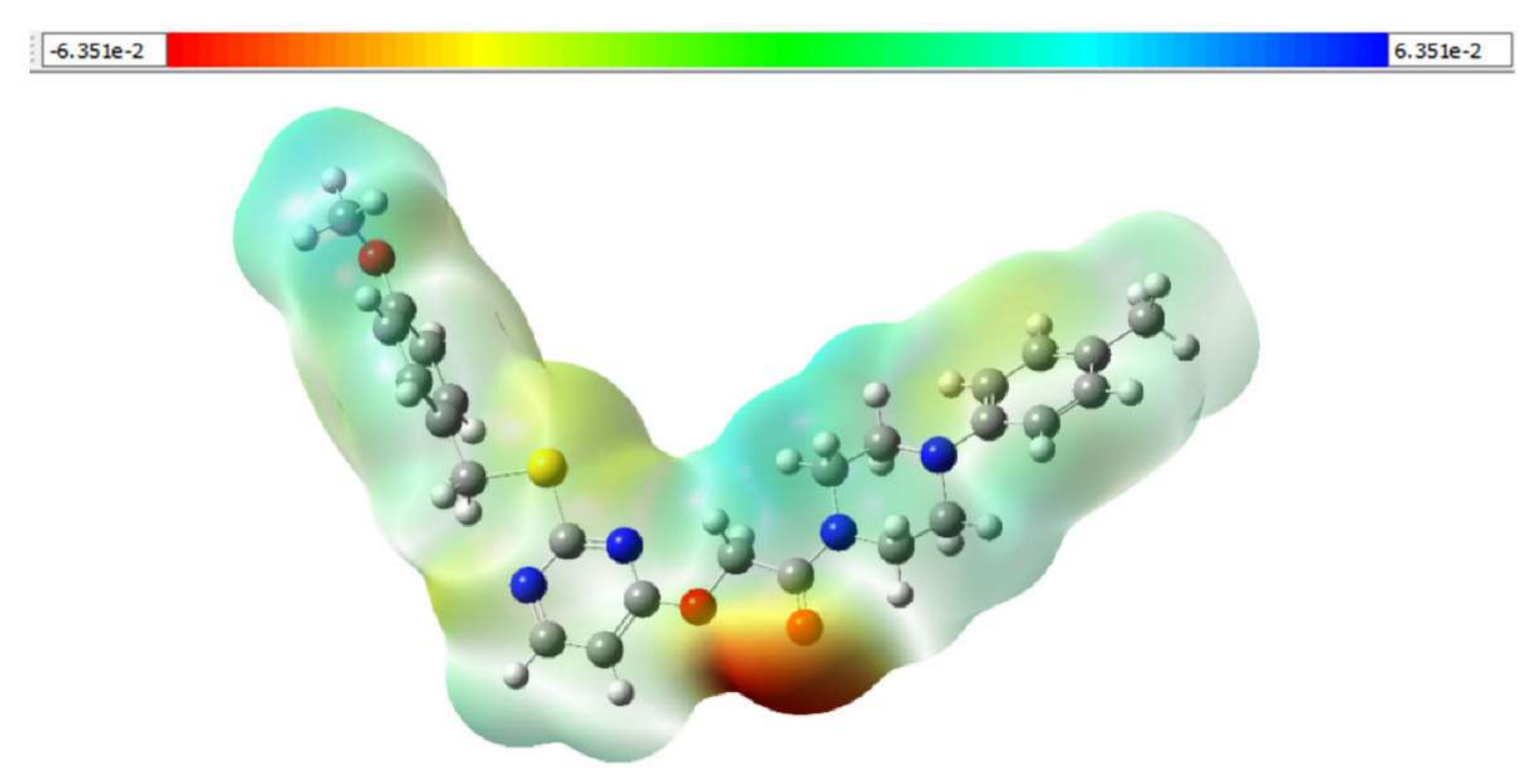
Molecular electrostatic potential surfaces of compound **5e**.

MEPs are fundamental measures of the interaction strength of the nearby charges, nuclei, and electrons at a particular position, thus enabling visualization of the charge distribution and charge-related characteristics of molecules. To facilitate interpretation of the electrostatic potential data, a visual representation with a chromatogram is used. The B3LYP/6-31+G (d,p) level of theory was used to study the molecular electrostatic potential in the intermolecular interaction region of conformer **5e**. The resulting MEP map with a range of −6.351 × 10^−2^–6.351 × 10^−2^ depicts its potential distribution (see Figure 5). The O atom of the C=O bond of molecule **5e** is surrounded by a positive charge, indicating a possible nucleophilic attack site. Furthermore, a negatively charged region is located on the hydrogen atoms of the piperazine ring, indicating a probable electrophilic attack site.

To determine the conformational stability of compounds at the PARP1 binding site, MD simulations are employed to assess structural and dynamic changes throughout the protein–ligand interactions [37]. The complex structures are solved using orthorhombic simple point charge (SPC) water models. On periodic boundary conditions, the system was determined to have a cut-off for Lennard–Jones interactions of 10 Å. In the integration steps, a time step of 2.0 fs was used. The system temperature and pressure were set to 300 K and 1.01325 bar, respectively, using the Nose–Hoover thermostat and Martyna–Tobias–Klein protocols. Before the production run, the system was minimized, equilibrated, and 100 ns MD simulations were then performed [38].

### 2.6. Bioinformatic Analysis of the Binding of Compound **5e** towards the Catalytical Grove of PARP1

**Figure 6:**
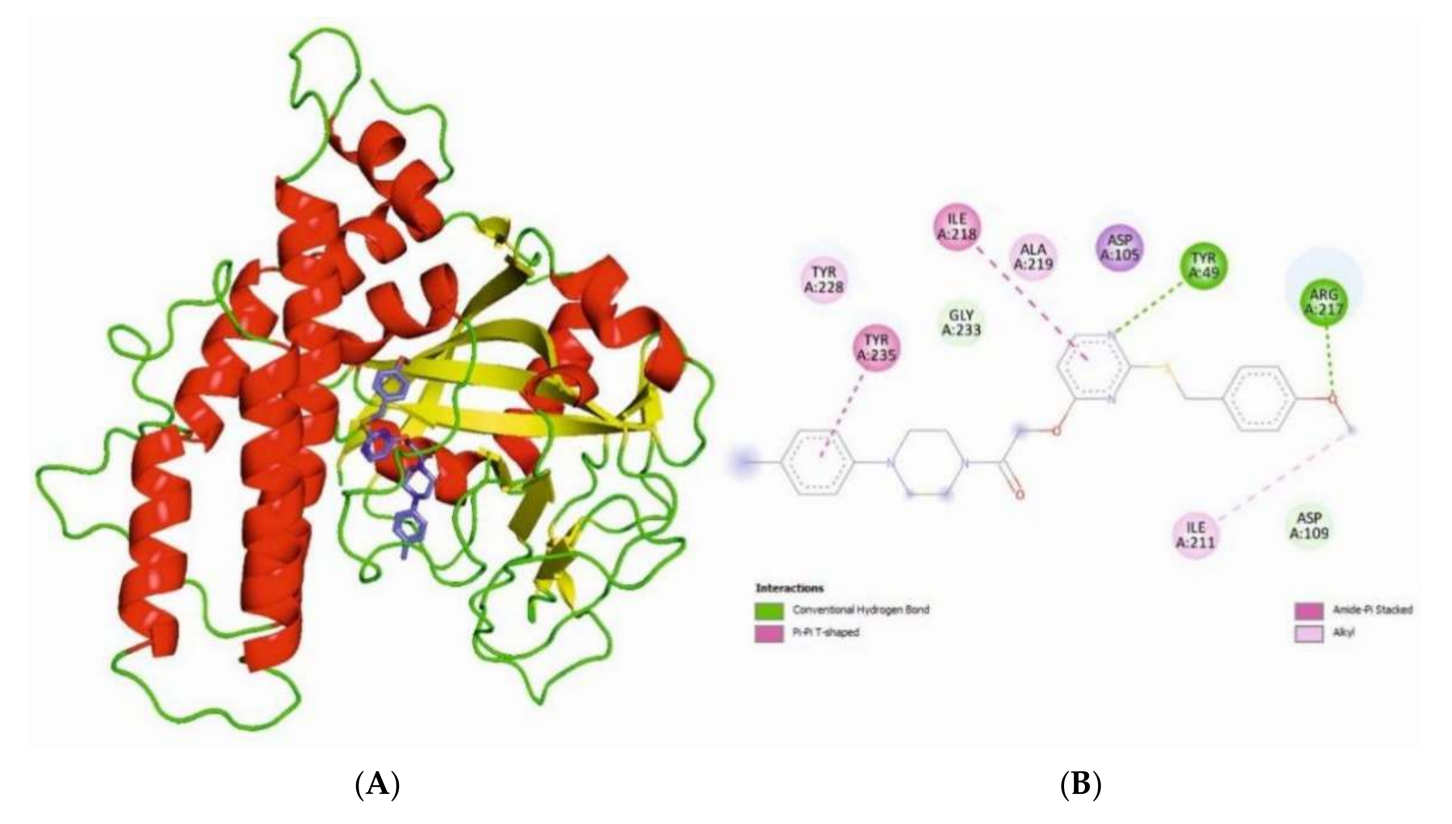
Cartoon representation of PARP1 with compound **5e** (**A**) and its 2D interactions (4HHY) (**B**).

**Figure 7:**
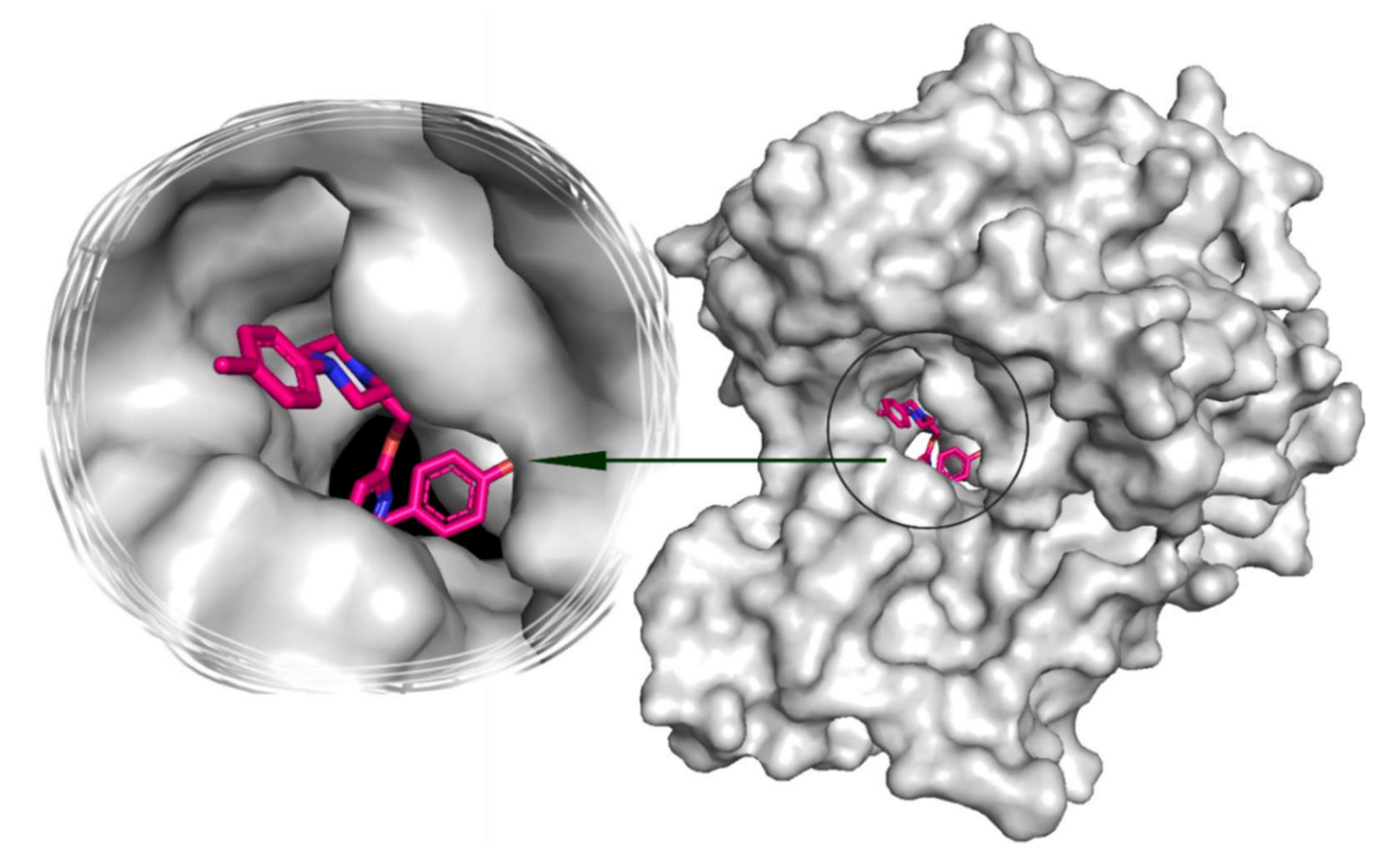
3D surface view of PARP1 and compound **5e** and a zoomed view of the compound **5e** in the active site cavity.

**Figure 8:**
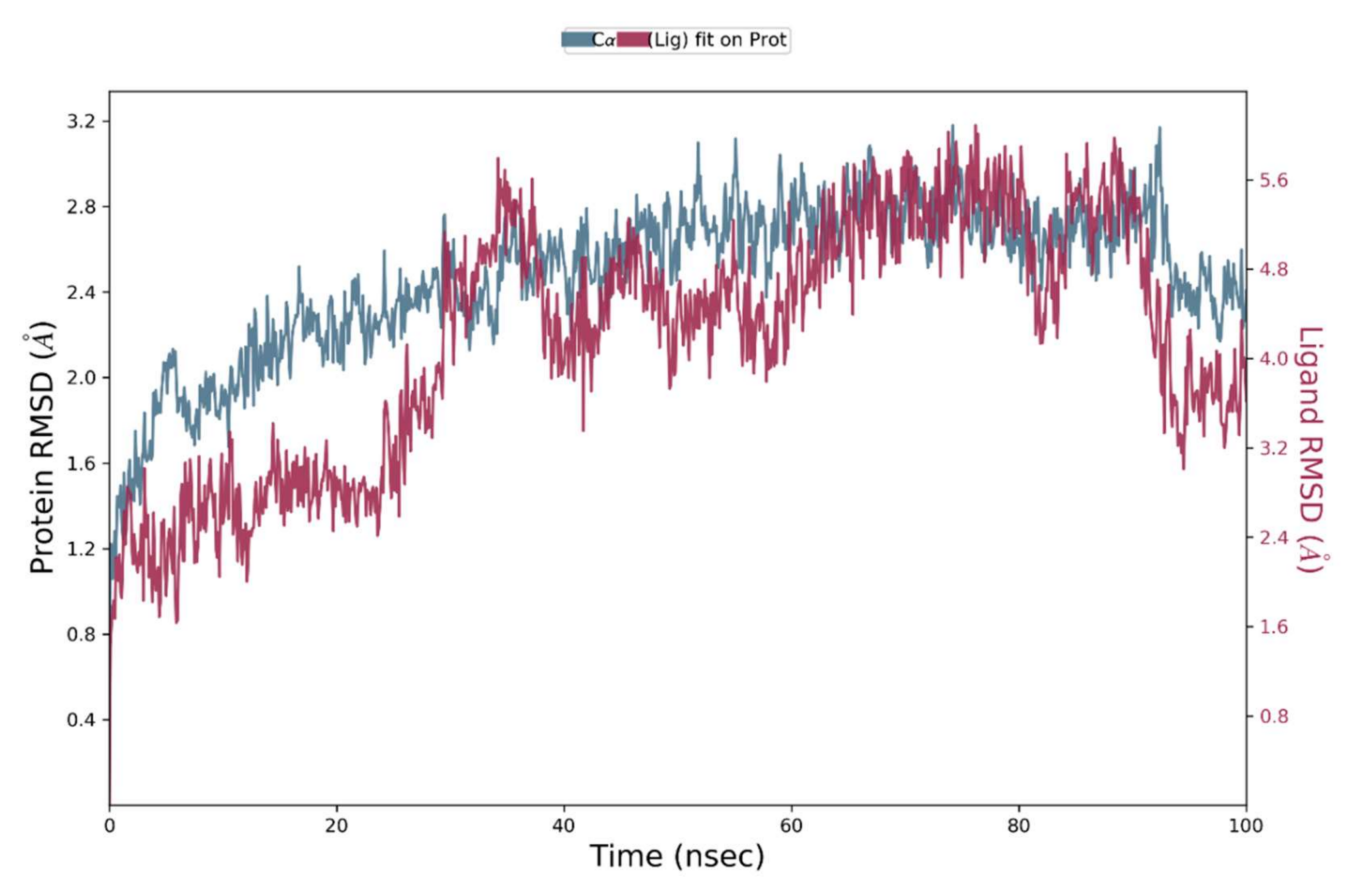
RMSD plot of ligand **5e** and PARP1.

Compound **5e** was studied using molecular docking modeling to assess the binding of compound **5e** towards the catalytical grove of PARP1 [39]. Figure 6A depicts the binding pose of compound **5e** with PARP1. Docking simulations revealed the prominent interactions of compound **5e** with PARP1 active site residues (Figure 6B). The docking score was assigned to compound **5e** that binds to the active site with −7.33 kcal/mol energy and forms potential hydrogen bonds. TYR49 is bonded with the nitrogen of the pyrimidine ring and ARG217 with the oxygen of methoxybenzene with the bond distances 2.50 and 3.56 Å (Figure 7), respectively. Furthermore, pi–pi stacking interactions exist with ILE218 and TYR235. In addition to the latter, hydrophobic interactions have been discovered in the active site [39]. 100 ns MD simulations were run for the molecule in the PARP1 binding pocket, and the associated coordinates throughout the MD simulation were gathered separately in the trajectory file. Figure 8 depicts the root-mean-square deviations (RMSDs) of C*α* atoms in the investigated systems. After 30 ns of MD simulations, all of the studied systems have relaxed and show no significant fluctuations in RMSD values. The evaluated system exhibited an average RMSD of less than 3.0 Å. The LigFitProt RMSD plot displays the RMSD of ligands when the protein–ligand complex is initially aligned as a reference state with the backbone atoms of the protein.

**Figure 9:**
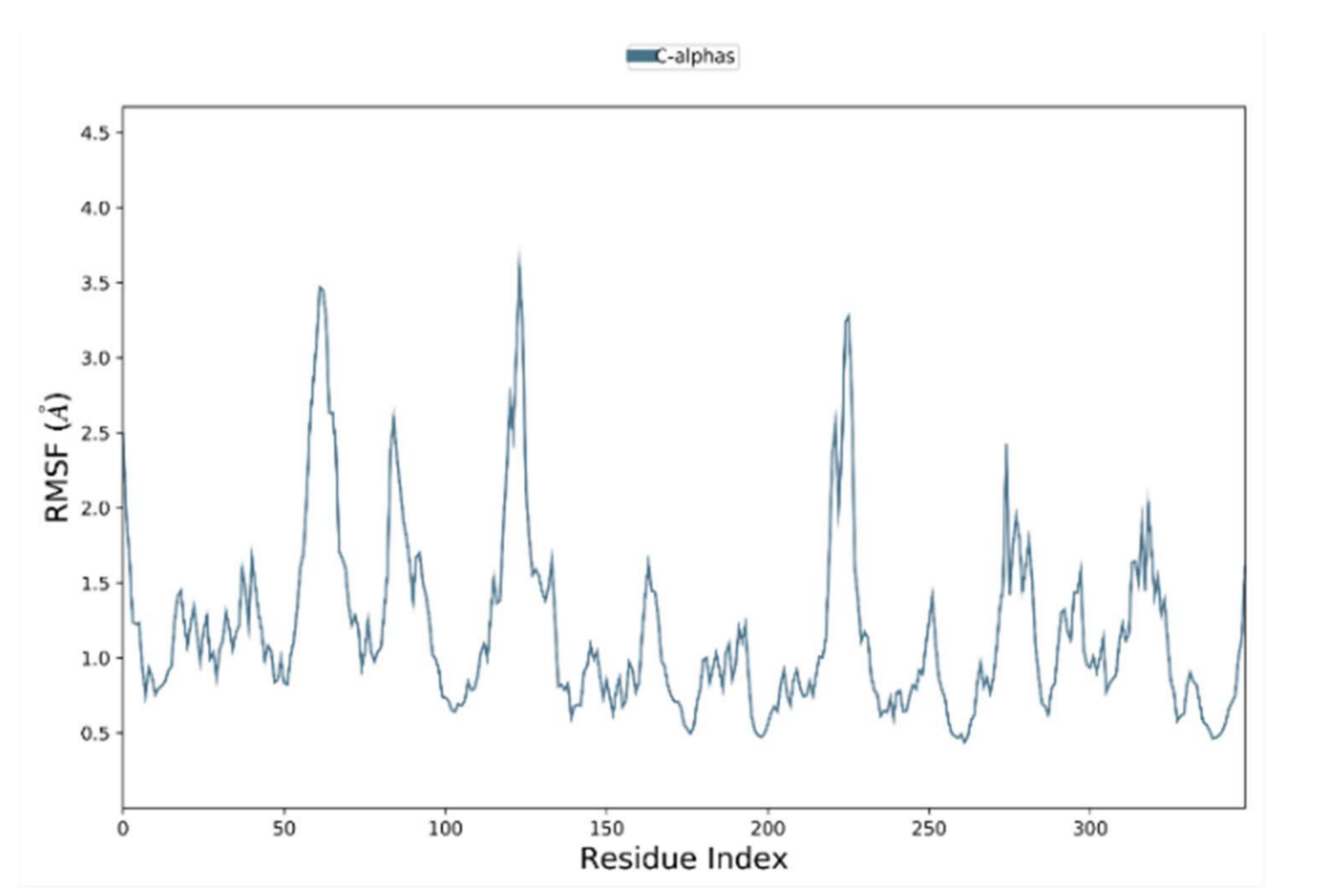
RMSF plot of protein molecule (4HHY) PARP1.

**Figure 10:**
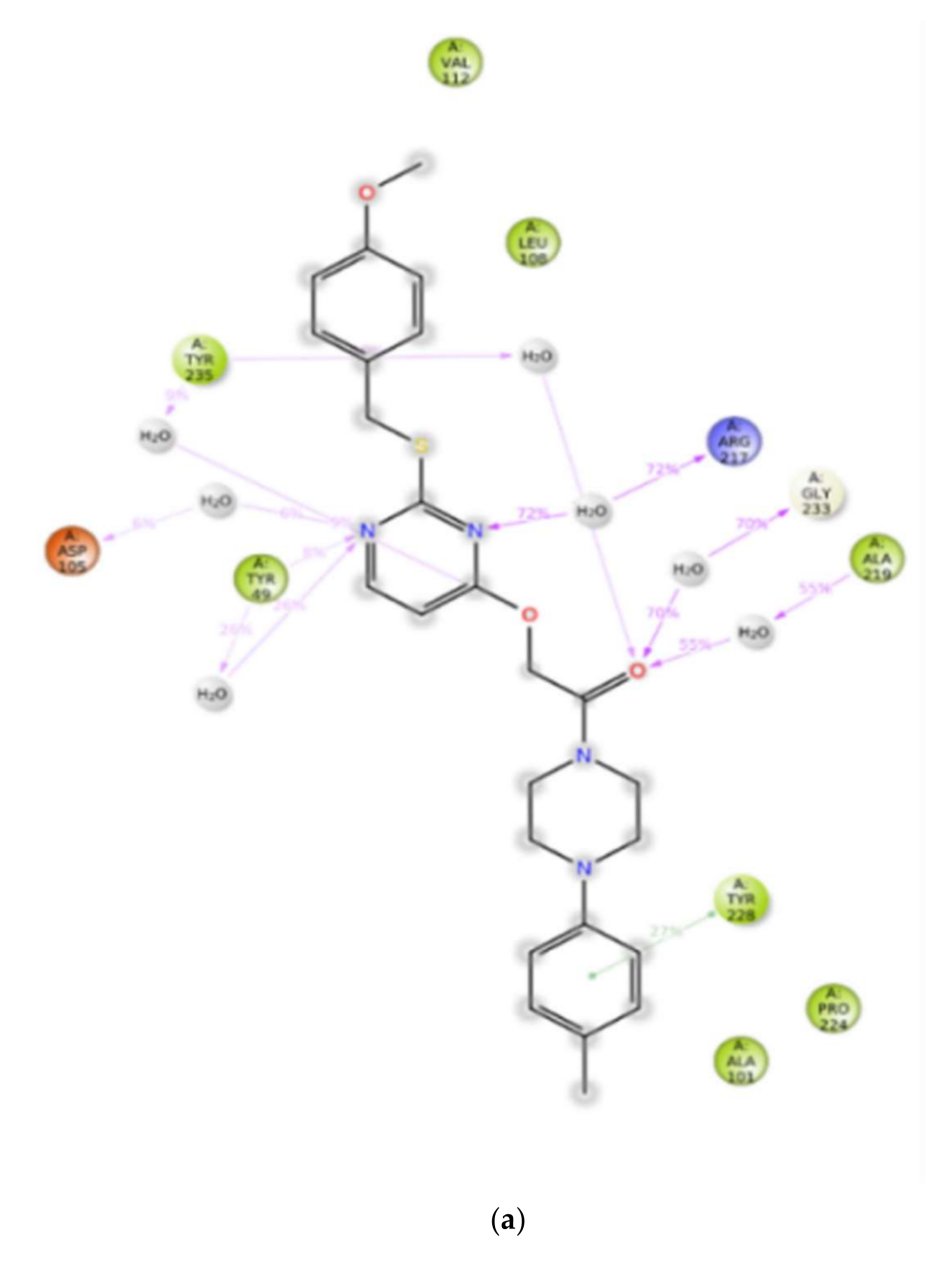

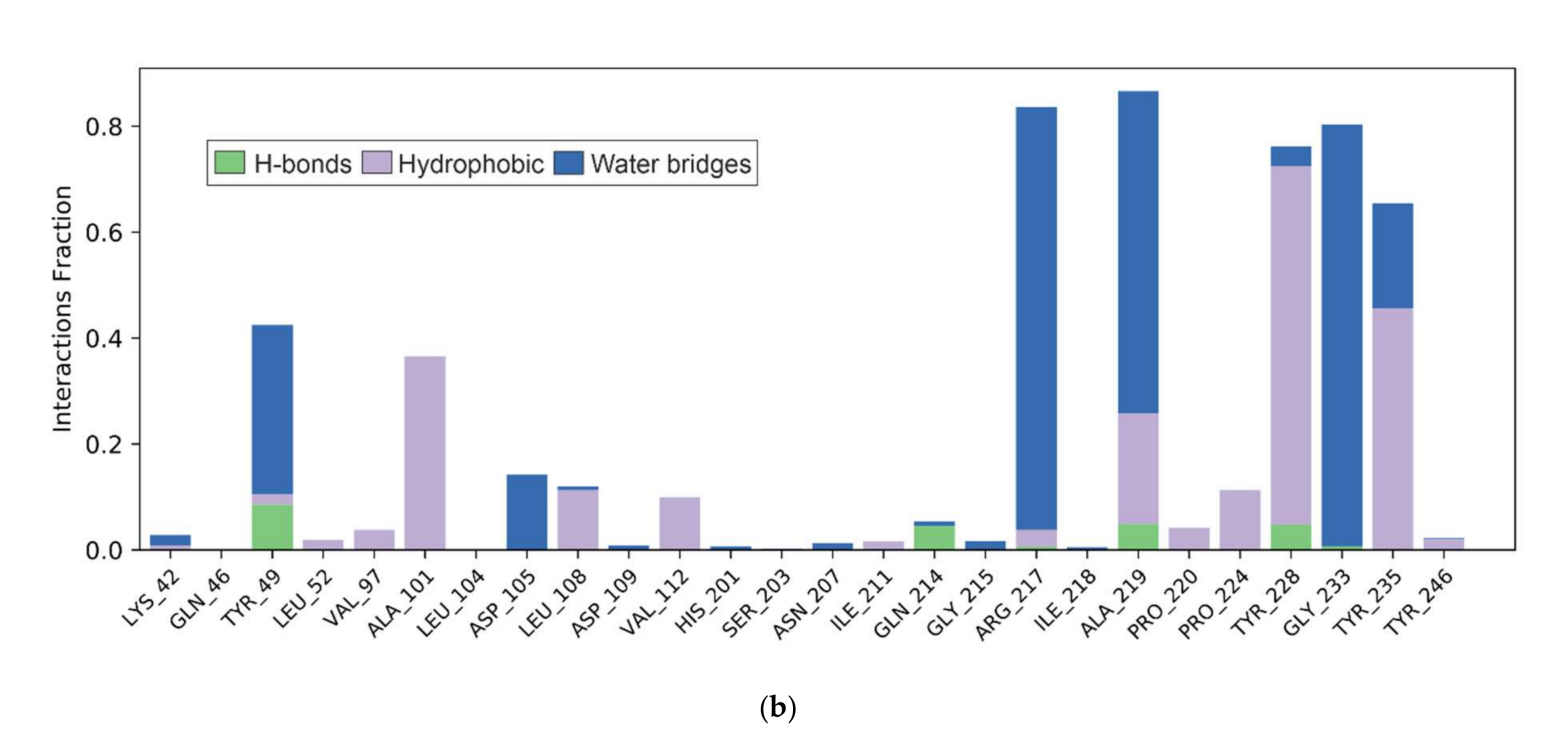
(**a**) Compound **5e**-PARP1 interaction and (**b**) histogram charts of the interaction percentages of the MD simulation for the complex.

Root-mean-square fluctuation (RMSF) values were also calculated during simulations to investigate the effect of defined hits on the mobility of the target protein’s backbone atoms (Figure 9). Figure 10A depicts associations that appear in the chosen path for more than 6% of the simulation time (0.00–100.00 ns). Hydrogen bonds with TYR49 and π–π stacking interactions with TYR228 produce significant interactions in the molecule. ASP105, ARG217, ALA219, GLY233, and TYR235 have stable non-bonded chemical contacts throughout the simulations, as shown by the protein–ligand interactions graph Figure 10B. The stability between compound **5e** and PARP1 is furthermore enhanced by the interaction with the amino acid residues ARG217, GLY233, ALA219, and TYR49 of PARP1 via water bridges [40].

## 3. Materials and Methods

All organic chemicals used were purchased from Sigma-Aldrich. ^1^H and ^13^C NMR spectra were recorded on a Bruker WH-200 (400 MHz) and JEOLJSM-ECS (400 MHz) spectrometer in CDCl_3_ as solvent, using TMS as an internal standard, and chemical shifts are expressed as ppm. High-resolution mass spectra were determined on a Bruker Daltonics instrument. Mass spectra were determined on an Agilent LC-MS and the elemental analyses were carried out using an Elemental Vario Cube CHNS rapid analyzer. The progress of the reaction was monitored by TLC precoated silica gel G plates [41].

### 3.1. Synthesis of Compound **2**

To a stirred solution of 2-thiouracil, p-methoxy benzyl bromide in DMF was added potassium carbonate, and the reaction mixture was heated to 80 °C. The reaction was monitored by thin-layer chromatography, quenched with water, and extracted with ethyl acetate. The organic layer was washed with brine and dried over sodium sulfate and concentrated under vacuum. The crude product was purified by column chromatography with silica-60 to yield the alcohol **2** in 81% yield.

### 3.2. Synthesis of Compound **3**

To a stirred solution of alcohol **2**, tert-butyl bromoacetate in DMF was added potassium carbonate (1.2 eq), and the reaction mixture was heated to 80 °C. The reaction was monitored by thin-layer chromatography. After completion of the reaction, it was quenched with water and extracted with ethyl acetate. The organic layer was washed with brine and dried over sodium sulfate and concentrated under vacuum. The crude product was purified by column chromatography with silica-60 to afford the ester **3** in 85% yield.

### 3.3. Synthesis of Compound **4**

To a stirred solution of ester **3** in dichloromethane, was added trifluoroacetic acid and stirred overnight. After completion of the reaction as monitored by thin-layer chromatography, it was quenched with sodium bicarbonate and extracted with ethyl acetate. The organic layer was washed with brine and dried over sodium sulfate and concentrated under vacuum. The crude product was purified by column chromatography with silica-60 to afford acid in 65% yield.

### 3.4. General Synthetic Method for the Preparation of **5a**–**5l**

To a stirred solution of the acid (100 mg, 0.33 mol) in dry dichloromethane (4 mL) under nitrogen atmosphere, was added EDC. HCl (93.7 mg, 0.49 mol). Then, DMAP (4 mg, 10 mol%), amine derivative (130.8 mg, 0.49 mol) was added to the reaction mixture and stirred for five minutes. Triethylamine (0.15 mL, 0.99 mol) was then added slowly, dropwise, to the reaction mixture and it was stirred for 2–3 h. After completion of the reaction, as indicated by TLC, it was quenched with water (5 mL) and extracted with ethyl acetate (2 × 10 mL). The combined organic layers were washed with brine (5 mL) and dried over anhydrous sodium sulfate. Evaporation of the solvent gave the crude residue which was purified by silica gel column chromatography using petroleum ether/EtOAc (3:7) as eluent to furnish amides **5a**–**5l**.

*1-(4-(2,3-Dichlorophenyl)piperazin-1-yl)-2-((2-((4-methoxybenzyl)thio)pyrimidin-4-yl)oxy)ethanone* (**5a**), Reddish yellow solid; MP: 145–148 °C; 88% yield; ^1^H NMR (400 MHz, CDCl_3_): *δ* 8.29 (d, *J* = 5.6 Hz, 1H), 7.30 (d, *J* = 8.4 Hz, 2H), 7.18 (d, *J* = 8.0 Hz, 1H), 7.11 (t, *J* = 8.0 Hz 1H), 6.83–6.78 (m, 3H), 6.58 (d, *J* = 5.6 Hz, 1H), 5.03 (s, 2H), 4.33 (s, 2H), 3.77 (s, 5H), 3.59 (s, 2H), 3.07 (s, 4H; ^13^C NMR (100 MHz, CDCl_3_): *δ* 173.1, 169.6, 167.3, 160.8, 159.7, 152.3, 150.3, 136.0, 132.0, 130.9, 129.7, 129.4, 127.2, 120.8, 120.5, 115.9, 105.9, 65.0, 57.2, 53.4, 47.1, 44.2, 41.2, 36.8; LCMS (ESI): *m*/*z* for C_24_H_24_Cl_2_N_4_O_3_S+H, calcd 519.0946; found: 519.0929.

*1-(4-(3,4-Difluorophenyl)piperazin-1-yl)-2-((2-((4-methoxybenzyl)thio)pyrimidin-4-yl)oxy)ethanone* (**5b**), Yellow white solid; MP: 135–138 °C; 92% yield; ^1^H NMR (400 MHz, CDCl_3_): *δ* 8.29 (d, *J* = 5.7 Hz, 1H), 7.28 (s, 1H), 7.02 (d, *J* = 9.5 Hz, 2H), 6.80 (d, *J* = 8.5 Hz, 2H), 6.61–6.51 (m, 3H), 5.02 (s, 2H), 4.31 (s, 2H), 3.76 (d, 5H), 3.58 (s, 2H), 3.07 (d, *J* = 16.2 Hz, 4H); ^13^C NMR (100 MHz, CDCl_3_): *δ* 167.8, 165.5, 159, 158.0, 148.0, 130.0, 129.1, 117.5, 114.1, 112.5, 106.3, 104.1, 63.11, 55.2, 50.1, 49.8, 44.8, 41.9, 40.0, 35.1, 22.8; LCMS (ESI): *m*/*z* for C_24_H_24_F_2_N_4_O_3_S+H, calcd 487.1537; found: 487.0645.

*1-(4-(4-Chloro-2-fluorophenyl)piperazin-1-yl)-2-((2-((4-methoxybenzyl)thio)pyrimidin-4-yl)oxy)ethanone* (**5c**), White solid; MP: 142–145 °C; 90% yield; ^1^H NMR (400 MHz, CDCl_3_): *δ* 8.29 (d, *J* = 5.6 Hz, 1H), 7.28 (d, *J* = 24.8 Hz, 2H), 7.05 (dd, *J* = 12, 2.4 Hz, 1H), 6.97 (d, *J =* 8.4 Hz, 1H) 6.82 (d, *J* = 8.8 Hz, 2H), 6.73 (m,1H), 6.58 (d, *J* = 5.6 Hz, 1H), 5.02 (s, 2H), 4.31 (s, 2H), 3.77 (s, 5H), 3.58 (m, 2H), 3.01 (m, 4H); ^13^C NMR (100 MHz, CDCl_3_): 171.2, 167.7, 165.3, 158.9, 157.8, 156.6, 154.1, 138.3, 138.2, 130.0, 129.0, 124.6, 124.5, 120.0, 117.1, 116.8, 114.0, 104.0, 63.0, 55.2, 50.7, 50.2, 42.0, 34.9.; LCMS (ESI): *m*/*z* for C_24_H_24_ClFN_4_O_3_S+H, calcd 503.1242; found: 503.0788.

*1-(4-Acetyl-1,4-diazepan-1-yl)-2-((2-((4-methoxybenzyl)thio)pyrimidin-4-yl)oxy)ethanone* (**5d**), ^1^H NMR (400 MHz, CDCl_3_): *δ* 8.27 (d, *J* = 5.8 Hz, 1H), 7.29 (d, *J* = 8.4 Hz, 2H), 6.82 (d, *J* = 8.2 Hz, 2H), 6.56 (d, *J* = 5.9 Hz, 1H), 5.00–4.96 (m, 2H), 4.31 (d, *J* = 5.0 Hz, 2H), 3.77 (s, 3H), 3.63–3.47 (m, 8H), 2.07 (d, *J* = 3.4 Hz, 3H), 1.90 (d, *J* = 31.1 Hz, 2H); ^13^C NMR (100 MHz, CDCl_3_): *δ* δ171.2, 170.7, 166.9, 159.1, 157.7, 130.1, 129.3, 114.1, 104.2, 63.2, 62.9, 55.4, 49.2, 48.6, 45.1,44.4, 34.9; LCMS (ESI): *m*/*z* for C_21_H_26_N_4_O_4_S+H, calcd 431.1675; found: 431.0702.

*2-((2-((4-Methoxybenzyl)thio)pyrimidin-4-yl)oxy)-1-(4-(p-tolyl)piperazin-1-yl)ethanone* (**5e**), ^1^H NMR (400 MHz, CDCl_3_): *δ* 8.29 (d, *J* = 5.6 Hz, 1H), 7.29 (d, *J* = 16.0 Hz, 2H), 7.07 (d, *J* = 8.0 Hz, 2H), 6.87–6.74 (m, 4H), 6.57 (d, *J* = 5.6 Hz, 1H), 5.03 (s, 2H), 4.32 (s, 2H), 3.78 (s, 5H), 3.57 (S, 2H), 3.16–3.02 (m, 4H), 2.28 (s, 3H); ^13^C NMR (100 MHz, CDCl_3_): *δ* 171.4, 167.9, 165.5, 159.0, 157.9, 130.5, 130.2 129.9, 117.4, 114.1, 104.1, 63.3, 55.4, 50.4, 50.1, 45.0, 42.1, 35.0, 20.6; LCMS (ESI): *m*/*z* for C_25_H_28_N_4_O_3_S+H, calcd 465.1882; found: 465.0852.

*1-(4-(2-Fluorophenyl)piperazin-1-yl)-2-((2-((4-methoxybenzyl)thio)pyrimidin-4-yl)oxy)ethanone* (**5f**), ^1^H NMR (400 MHz, CDCl_3_): *δ* 8.28 (d, *J* = 5.6 Hz, 1H), 7.29 (d, *J* = 8.8 Hz, 2H), 7.05–6.94 (m, 4H), 6.86–6.81 (m, 3H), 6.57 (d, *J* = 5.6 Hz, 1H), 5.03 (s, 2H), 4.32 (s, 2H), 3.77 (s, 5H), 3.58 (s, 2H), 3.12–3.02 (m, 4H); ^13^C NMR (100 MHz, CDCl_3_): *δ* 171.2, 167.8, 165.3, 158.8, 157.8, 157.0, 139.4, 139.3, 130.1, 130.0, 129.5, 128.5, 125.1, 123.3, 119.3, 117.6, 116.3, 113.5, 103.5, 68.1, 63.1, 55.3, 50.8, 50.3, 45.0, 42.1, 34.8; LCMS (ESI): *m*/*z* for C_24_H_25_FN_4_O_3_S+H, calcd 469.1631; found: 469.2454.

*2-((2-((4-Methoxybenzyl)thio)pyrimidin-4-yl)oxy)-N-(5-(trifluoromethyl)-1,3,4-thiadiazol-2-yl)acetamide* (**5g**), ^1^H NMR (400 MHz, CDCl_3_): δ 8.33 (d, *J* = 5.3 Hz, 1H), 7.18 (d, *J* = 8.0 Hz, 2H), 6.74 (d, *J* = 8.0 Hz, 2H), 6.62 (d, *J* = 5.4 Hz, 1H), 5.21 (s, 2H), 4.23 (s, 2H), 3.73 (s, 3H); ^13^C NMR (100 MHz, CDCl_3_): *δ* 171.1, 166.6, 166.4, 161.0, 158.3, 157.7, 129.5, 129.3, 128.2, 113.4, 103.4, 63.6, 54.7, 34.2; LCMS (ESI): *m*/*z* for C_17_H_14_F_3_N_5_O_3_S_2_+H, calcd 458.0490; found: 458.1220.

*N-(Furan-2-ylmethyl)-2-((2-((4-methoxybenzyl)thio)pyrimidin-4-yl)oxy)acetamide* (**5h**), ^1^H NMR (400 MHz, CDCl_3_): *δ* 8.29 (d, *J* = 5.6 Hz, 1H), 7.40–7.26 (m, 3H), 6.83 (d, *J* = 8 Hz, 2H), 6.58 (brs, 1H), 6.48 (d, *J* = 6.0 Hz, 1H), 6.32 (s, 1H), 6.24 (s, 1H), 4.86 (s, 2H), 4.51 (d, *J* = 5.6 Hz, 2H), 4.31 (s, 2H), 3.78 (s, 3H); ^13^C NMR (100 MHz, CDCl_3_): *δ* 172.1, 167.0, 158.8, 158.1, 150.6, 142.3, 130.8, 130.1, 1291, 128.8, 114.5, 110.5, 107.7, 103.4, 68.1, 64.8, 55.2, 36.0, 34.8; LCMS (ESI): *m*/*z* for C_19_H_19_N_3_O_4_S+H, calcd 386.1096; found: 386.1792.

*2-((2-((4-Methoxybenzyl)thio)pyrimidin-4-yl)oxy)-N-(1,2,3,4-tetrahydronaphthalen-1-yl)acetamide* (**5i**), ^1^H NMR (400 MHz, CDCl_3_): *δ* 8.27 (d, *J* = 5.7 Hz, 1H), 7.70 (dd, *J* = 5.7, 3.3 Hz, 1H), 7.53 (dd, *J* = 5.7, 3.3 Hz, 1H), 7.34 (d, *J* = 8.6 Hz, 2H), 7.18 (s, 1H), 7.11 (s, 1H), 6.85 (d, *J* = 8.6 Hz, 2H), 6.44 (d, *J* = 5.7 Hz, 1H), 4.89 (d, *J* = 3.5 Hz, 2H), 4.35 (s, 2H), 4.22 (m,1H), 3.78 (s, 3H), 2.79 (dd, *J* = 12.4, 5.9 Hz, 2H), 1.81 (dd, *J* = 12.0, 6.7 Hz, 4H); ^13^C NMR (100 MHz, CDCl_3_): *δ* 171.9, 167.7, 167.1, 166.6, 158.8, 158.0, 137.63, 136.1, 130.84, 130.0, 129.2, 129.0, 128.43, 128.4, 128.2, 127.4, 126.4, 113.9, 103.5, 68.1, 55.3, 47.3, 38.7, 34.9, 20.1.

*2-((2-((4-Chlorobenzyl)thio)-6-methylpyrimidin-4-yl)oxy)-1-(4-(3,4-dichlorophenyl)piperazin-1-yl)ethan-1-one* (**5j**), Off white solid; MP: 135–138 °C; 88% yield; ^1^H NMR (400 MHz, CDCl_3_): *δ* 7.32 (d, *J* = 8.4 Hz, 2H), 7.24 (d, *J* = 8.2 Hz, 2H), 7.19 (dd, *J* = 8.0, 1.1 Hz, 1H), 7.10 (t, *J* = 8.0 Hz, 1H), 6.80 (dd, *J* = 8.0, 1.1 Hz, 1H), 6.42 (s, 1H), 4.99 (s, 2H), 4.32 (s, 2H), 3.76 (s, 2H), 3.57 (s, 2H), 3.03–2.94 (m, 4H), 2.38 (s, 3H); ^13^C NMR (100 MHz, CDCl_3_): *δ* 169.8, 168.4, 165.6, 150.3, 136.4, 134.2, 133.0, 131.1, 130.1, 128.2, 127.8, 127.5, 125.3, 118.8, 102.7, 63.1, 51.2, 50.9, 50.7, 45.1, 42.2, 34.5, 30.3, 23.8. LCMS (ESI): *m*/*z* for C_24_H_23_Cl_3_N_4_O_2_S, calcd 537.8890; found:537.0455.

*1-(4-(4-Chlorophenyl)piperazin-1-yl)-2-((2-((4-fluorobenzyl)thio)-6-methylpyrimidin-4-yl)oxy)ethanone* (**5k**), Off white solid; MP: 138–142 °C; 90% ^1^H NMR (400 MHz, CDCl_3_): *δ* 7.34 (dd, *J* = 8.1, 5.6 Hz, 2H), 7.22 (d, *J* = 8.8 Hz, 2H), 6.96 (t, *J* = 8.6 Hz, 2H), 6.82 (d, *J* = 8.5 Hz, 2H), 6.43 (s, 1H), 5.00 (s, 2H), 4.32 (s, 2H), 3.75 (s, 2H), 3.59 (s, 2H), 3.12 (d, *J* = 20.8 Hz, 4H), 2.40 (s, 3H). LCMS (ESI): *m*/*z* for C_24_H_24_ClFN_4_O_2_S, calcd 486.9894; found: 487.1152.

*2-((2-((4-Fluorobenzyl)thio)-6-methylpyrimidin-4-yl)oxy)-1-(4-(p-tolyl)piperazin-1-yl)ethanone* (**5l**), Yellowish white solid; MP: 134–137 °C; 88% yield; ^1^H NMR (400 MHz, CDCl_3_): *δ* 7.35 (dd, *J* = 8.4, 5.5 Hz, 2H), 7.09 (d, *J* = 8.3 Hz, 2H), 6.96 (t, *J* = 8.6 Hz, 2H), 6.83 (d, *J* = 5.1 Hz, 2H), 6.43 (s, 1H), 5.01 (s, 2H), 4.33 (s, 2H), 3.76 (s, 2H), 3.59 (s, 2H), 3.10 (d, *J* = 17.7 Hz, 4H), 2.40 (s, 3H), 2.29 (s, 3H). LCMS (ESI): *m*/*z* for C_25_H_27_FN_4_O_2_S, calcd 466.5709; found: 467.1684.

### 3.5. Cell Viability Assay

MCF-7 and MCF-10A cells were procured from Procell Life Science and Technology Co. LTD, China. Cells (2000) were cultured in MEM or Leibovitz’s L-15 medium enriched with 2% FBS, MCF-10A cells were cultured in DMEM with 5% horse serum (HS) (general culture: 2% HS was used for experimental conditions), 20 ng/mL EGF, 0.5μg/mL hydrocortisone, 10μg/mL insulin, 1% NEAA (non-essential amino acids); and maintained in a humidified atmosphere of 5% CO_2_ at 37 °C. DMSO-dissolved compounds were stored as a stock solution, and diluted with cell culture medium as required. Cells (4 × 10^3^) were cultured for 12 h in 96-well plates and treated with thiouracil amides or Olaparib at 0, 0.01, 0.1, 10, 100, and 1000 µM concentrations for 72 h. The inhibitory effect of the compounds was assessed using Alamar Blue [42,43,44,45].

### 3.6. Assay of PARP1 Catalytic Activity

The catalytic activity of PARP1 was determined using the PARP/Apoptosis Universal Colorimetric Assay Kit (4677-096-K, R&D Systems Ltd., Abingdon, UK) [46]. Compounds **5a** or **5e** or Olaparib were added to recombinant human PARP enzyme prior to evaluation as per manufacturer’s instructions.

### 3.7. Western Blot Analysis

Western blot analysis was performed to determine the levels of cleaved PARP1, and the phosphorylation ofH2AX in MCF-7 cells using previously reported methods [29]. Cells at 50–60% confluence were treated with compounds and incubated for 60 h in 2% FBS. MCF-7 cells were also treated with Olaparib, **5a**, or **5e,** and the levels of cleaved PARP1 and the phosphorylation of p-H2AX were determined using the respective antibodies, as previous [29].

### 3.8. CASPASE-3/7 Activity Assay

CASPASE-3/7 activity was determined by the Caspase-Glo^®^ 3/7 assay kit (Promega corporation, Madison, WI, USA) according to the manufacturer’s instructions [30]. MCF-7 cells were seeded in 96-well plates and incubated overnight. Compounds or vehicle were added to cells after 12 h. After a 24 h incubation period, Caspase-Glo^®^ 3/7 reagent and media (1:1) were added into each well and processed according to the manufacturer’s instructions.

### 3.9. DFT Calculations for the Compound **5e**

Computational DFT studies for the **5e** molecule were performed using Gaussian09 [47]. The molecule was built and visualized by Gaussview 5 program package. The molecular geometry of the **5e** molecule was optimized at B3LYP (Becke’s three parameter (B3)) level with a 6-311+G (d,p) basis set [48].

### 3.10. Bioinformatics Studies for the Compound **5e**

The Scripps Research Institute’s AutoDock Tools (ADT) (v1.5.7) [49,50] was used to generate grid and docking parameter files.The crystal structure of PARP1 with the ligand was used to retrieve the crystal structure of PARP1 with a ligand (PDB ID: 4HHY) and use it as afor the docking purpose (PDB ID: 4HHY). With the centroid rendered from inhibitor in the crystallographic structure, the grid box size of 65 × 65 × 65 Å with 0.481 Å spacing is defined. The empirical free-energy function and the Lamarckian genetic algorithm were used to perform molecular docking with the macromolecule, with an initial population of 150 randomly placed individuals, a maximum number of 2,500,000 energy evaluations, a mutation rate of 0.02, a crossover rate of 0.80, and 10 docking runs. Visualization plots were examined using BIOVIA Discovery Studio Visualizer (v21.10.20298) [51] and PyMOL (v2.5.2) [52,53].

### 3.11. Statistical Analysis

The data were analyzed by Student’s *t*-test and *p* < 0.05 was considered statistically significant (GraphPad Prism 5.0; GraphPad Software, La Jolla, CA, USA).

## 4. Conclusions

A series of thiouracil derivatives containing amide linkages were synthesized and screened for loss of viability of breast cancer cells. The compounds exhibited moderate to significant activity in MCF-7 cells. Compounds **5a** and **5e** inhibited PARP1 activity, produced PARP cleavage, and enhanced phosphorylation of H2AX. Computational methodologies demonstrated conclusively that compound **5e** interacts with PARP.

## Figures and Tables

**Table 1:** Thiouracil amides structure and their cytotoxicity results tabulated.

Entry	Acid	Amine (R_2_)	Structure	MCF-7 (IC_50_ µM)
**5a**	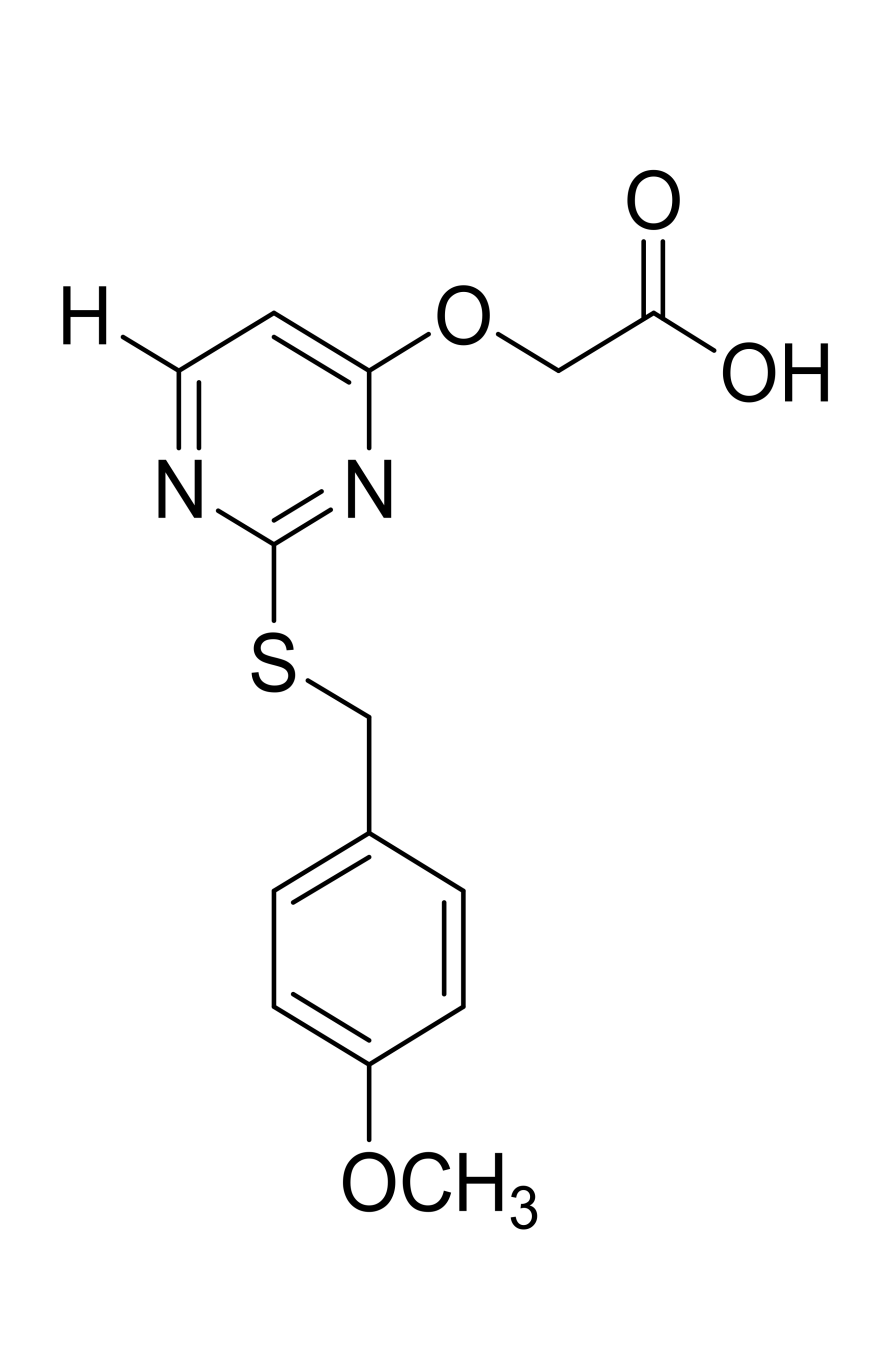	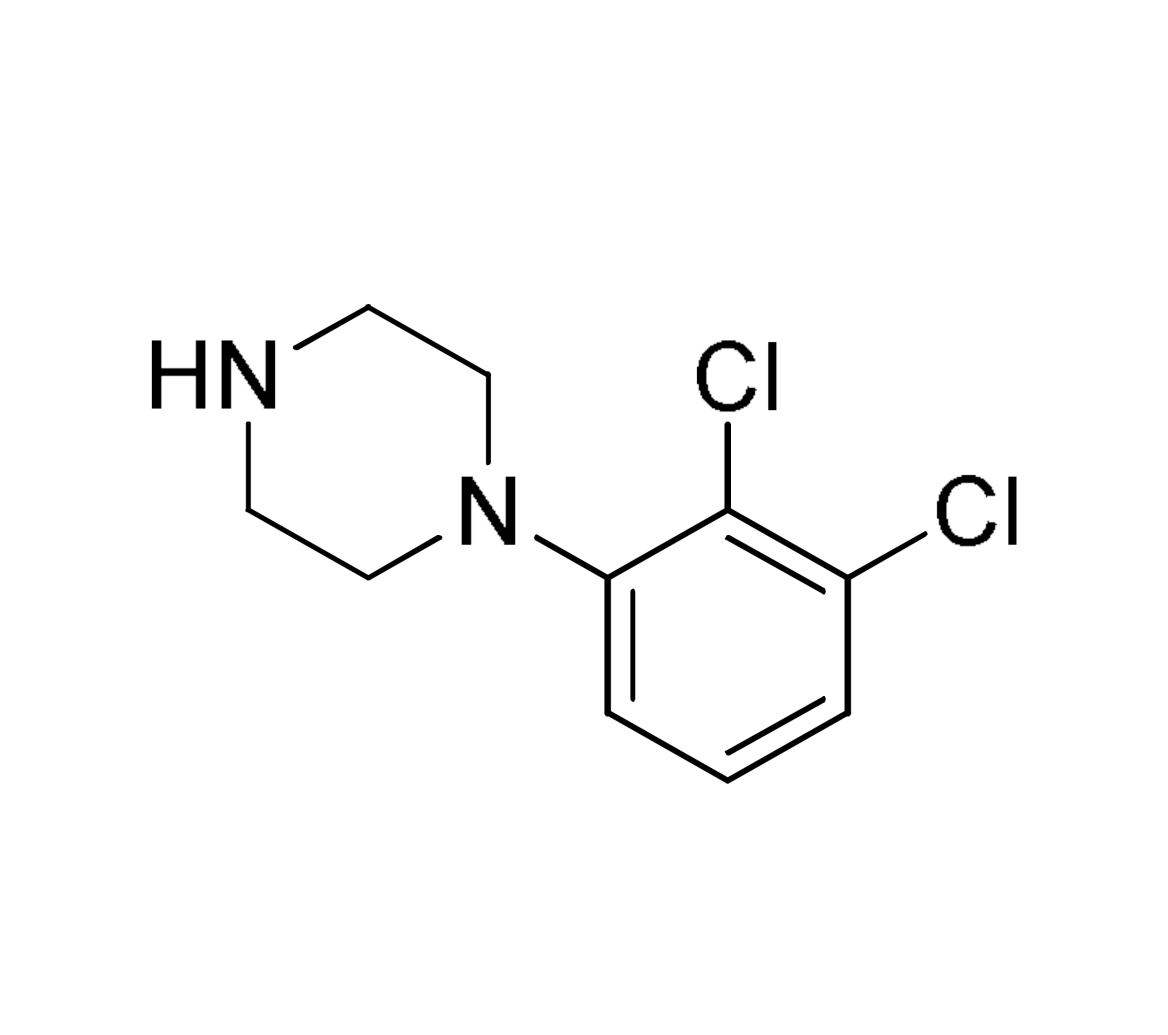	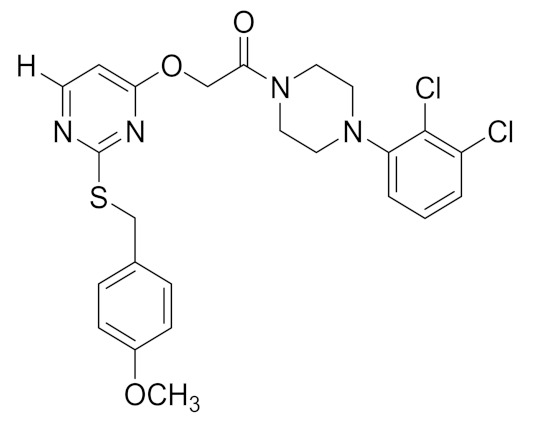	**22.68**
**5b**		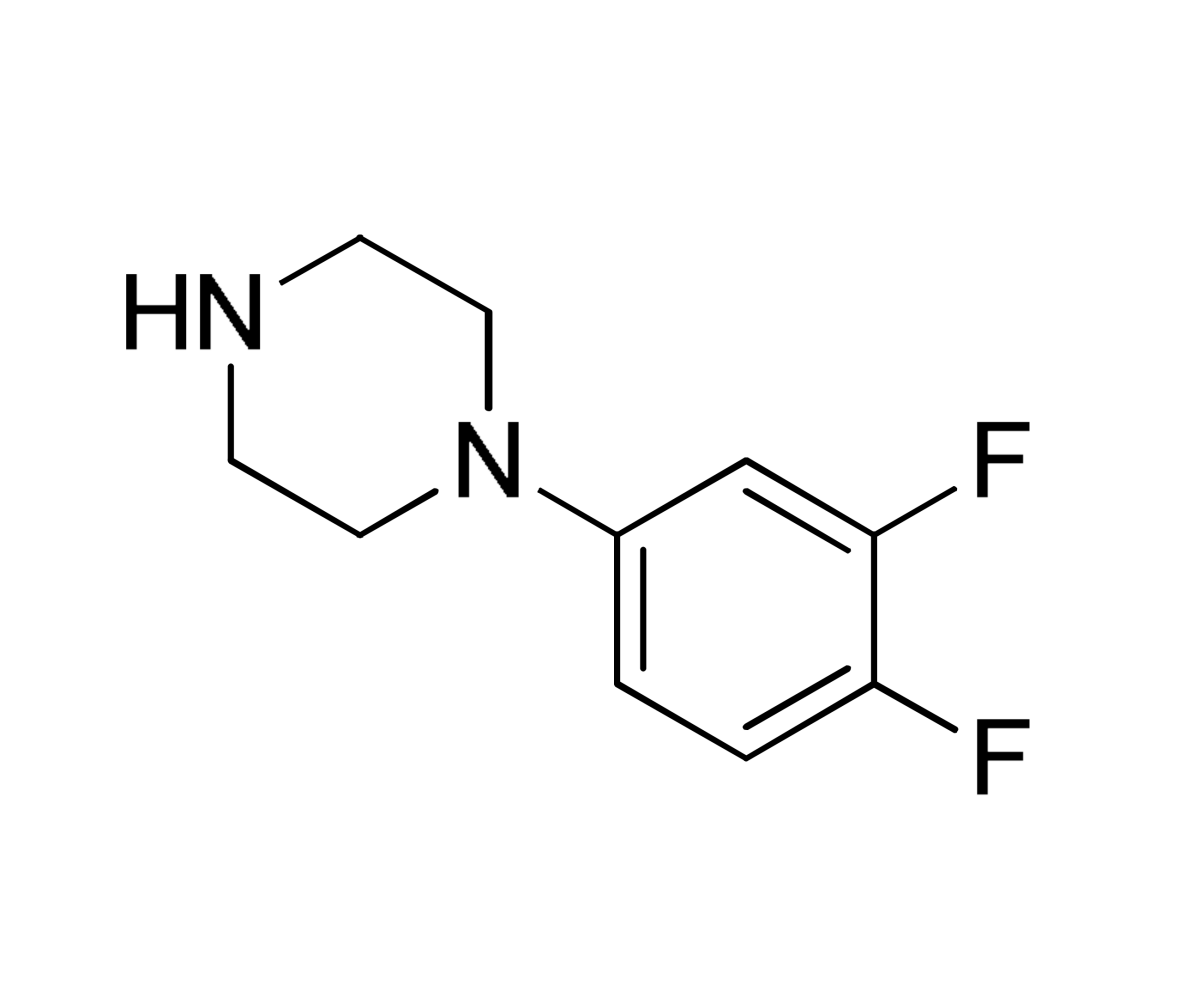	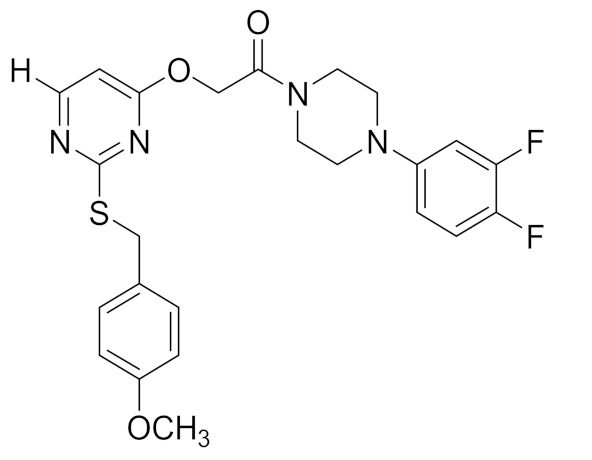	**25.71**
**5c**		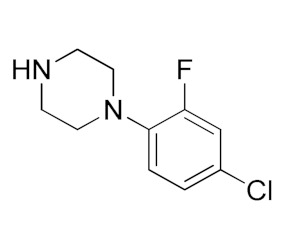	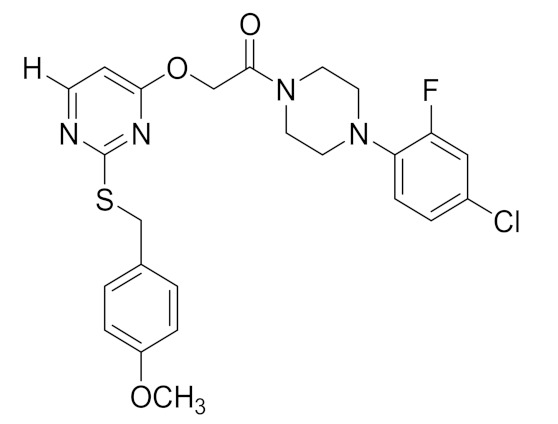	49.72
**5d**		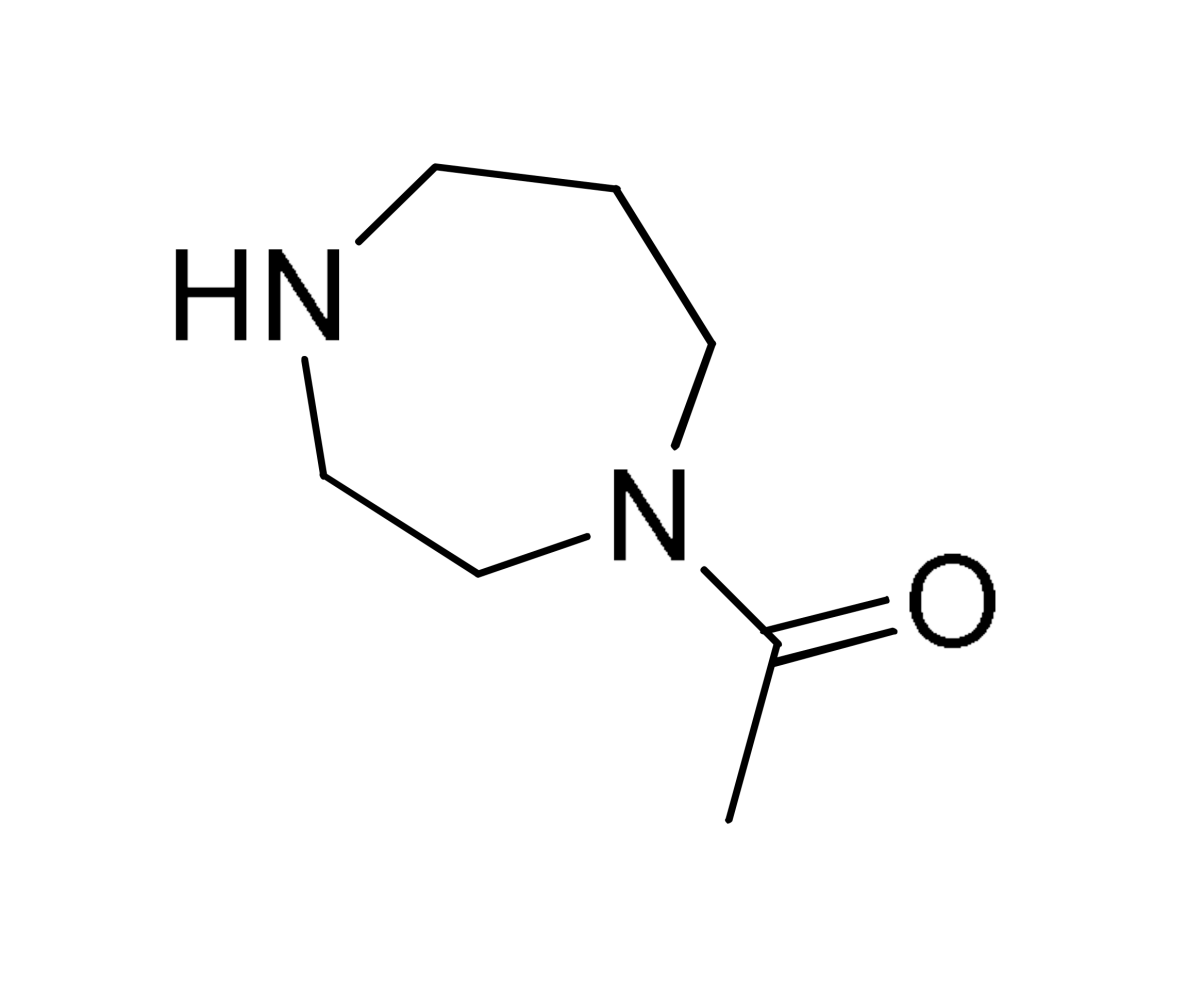	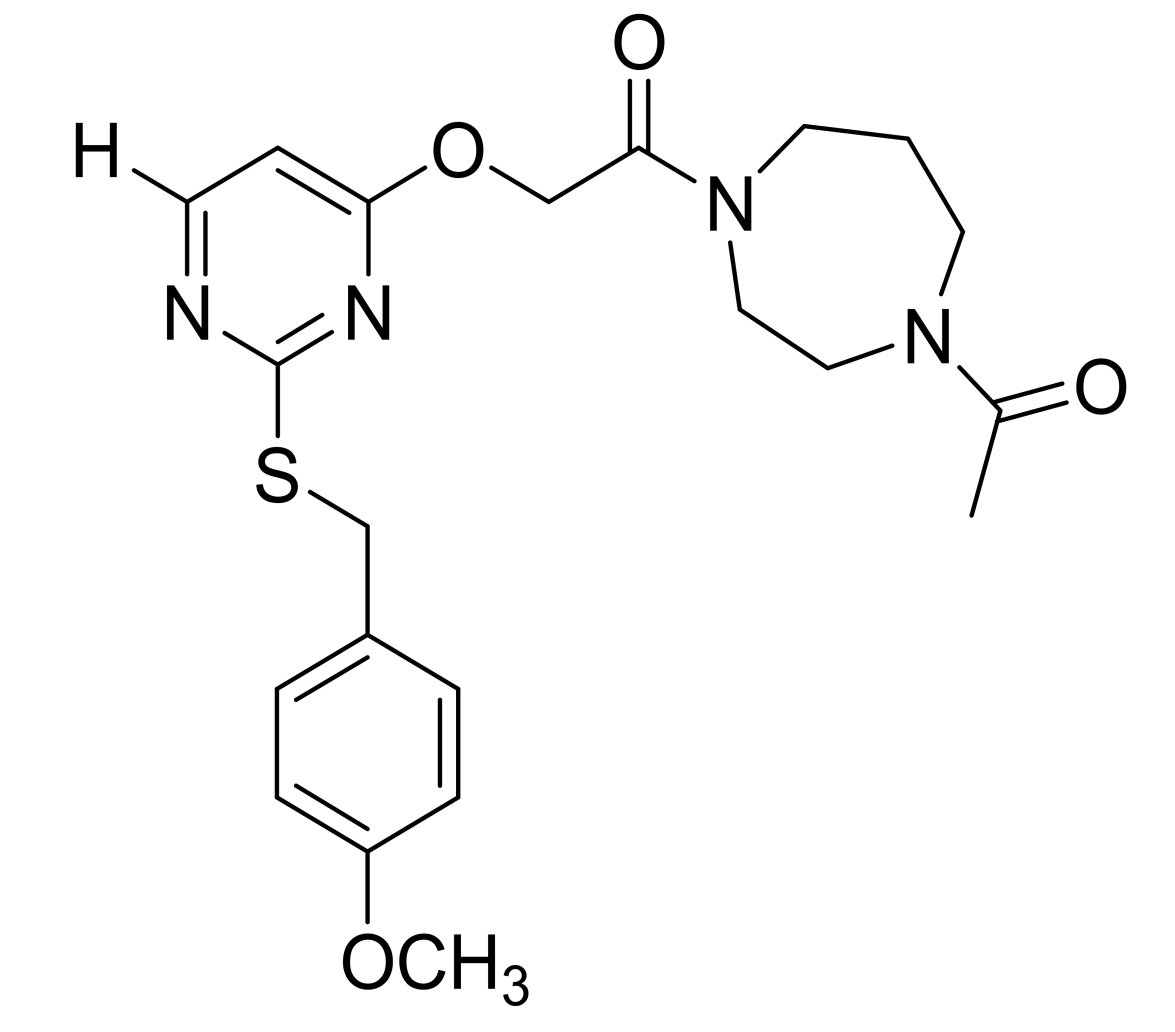	106.20
**5e**		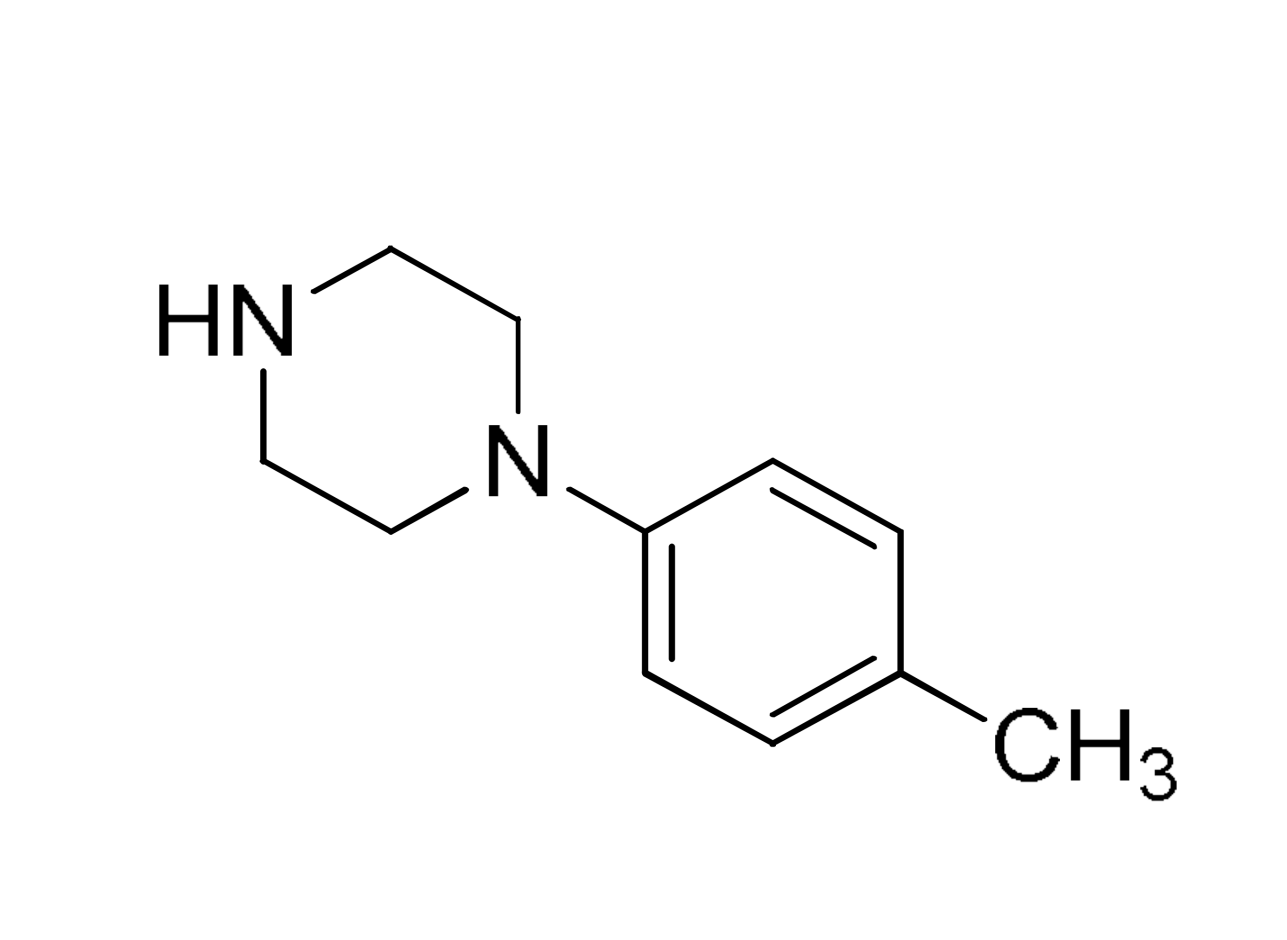	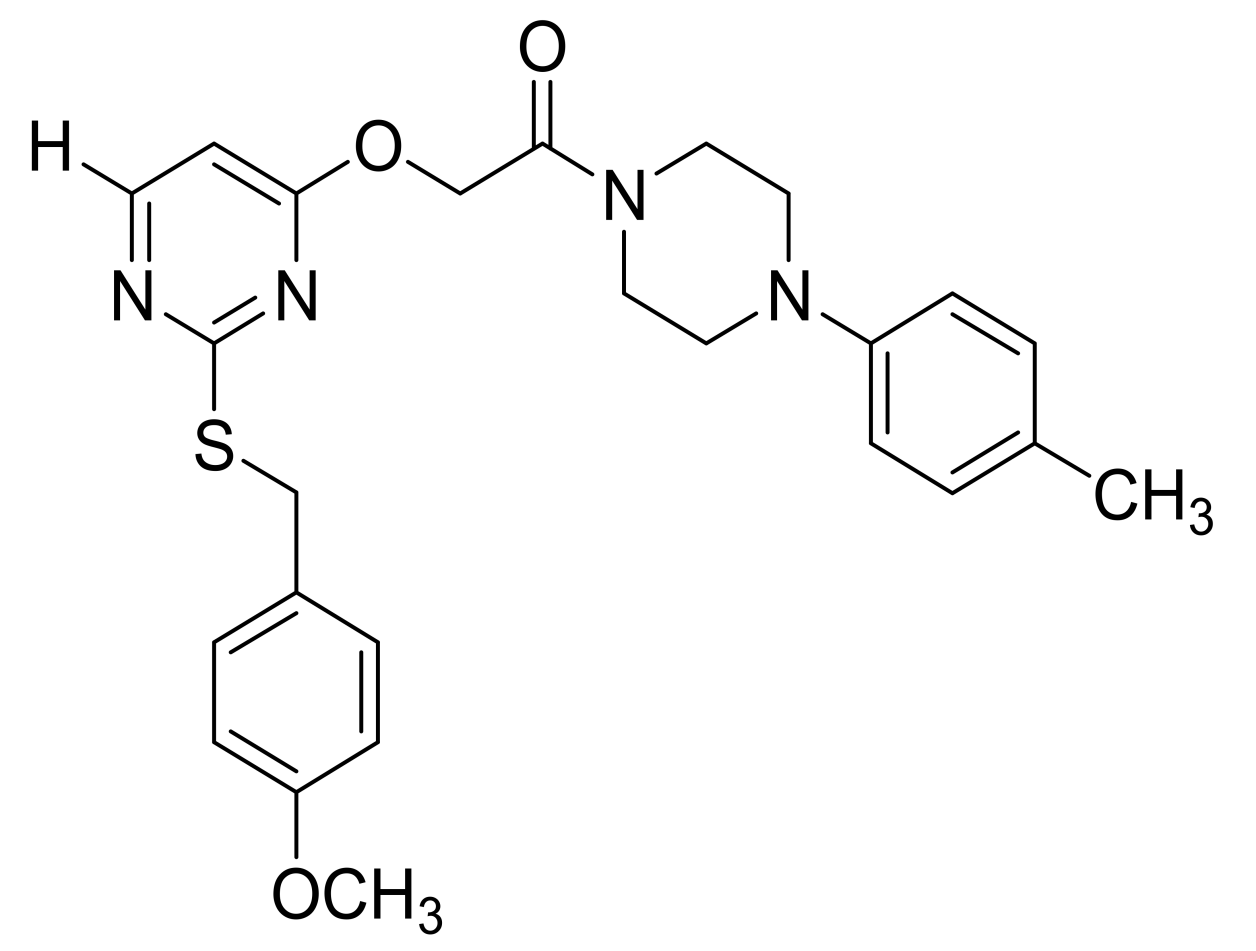	**18.23**
			**Lead molecule**	
**5f**		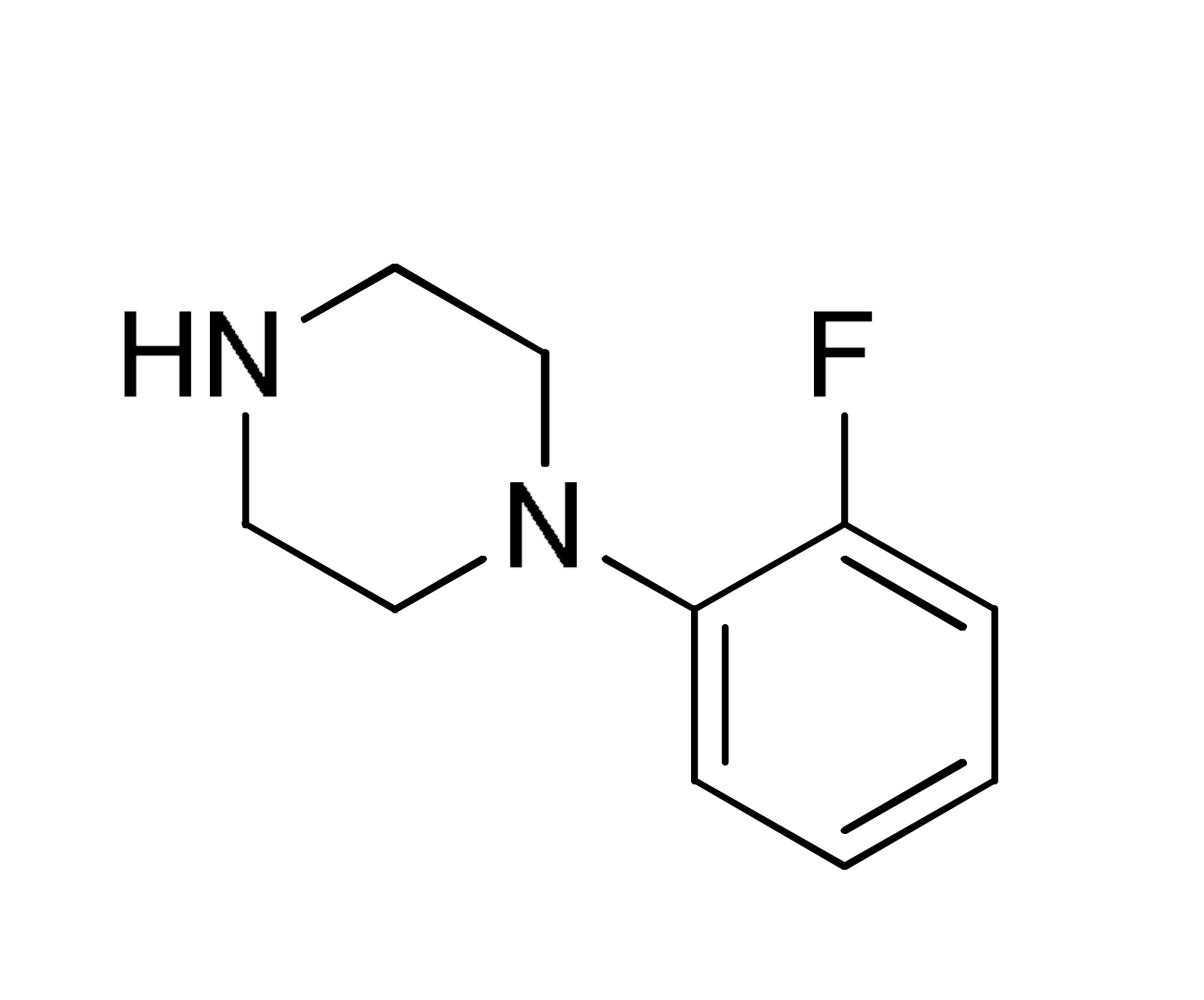	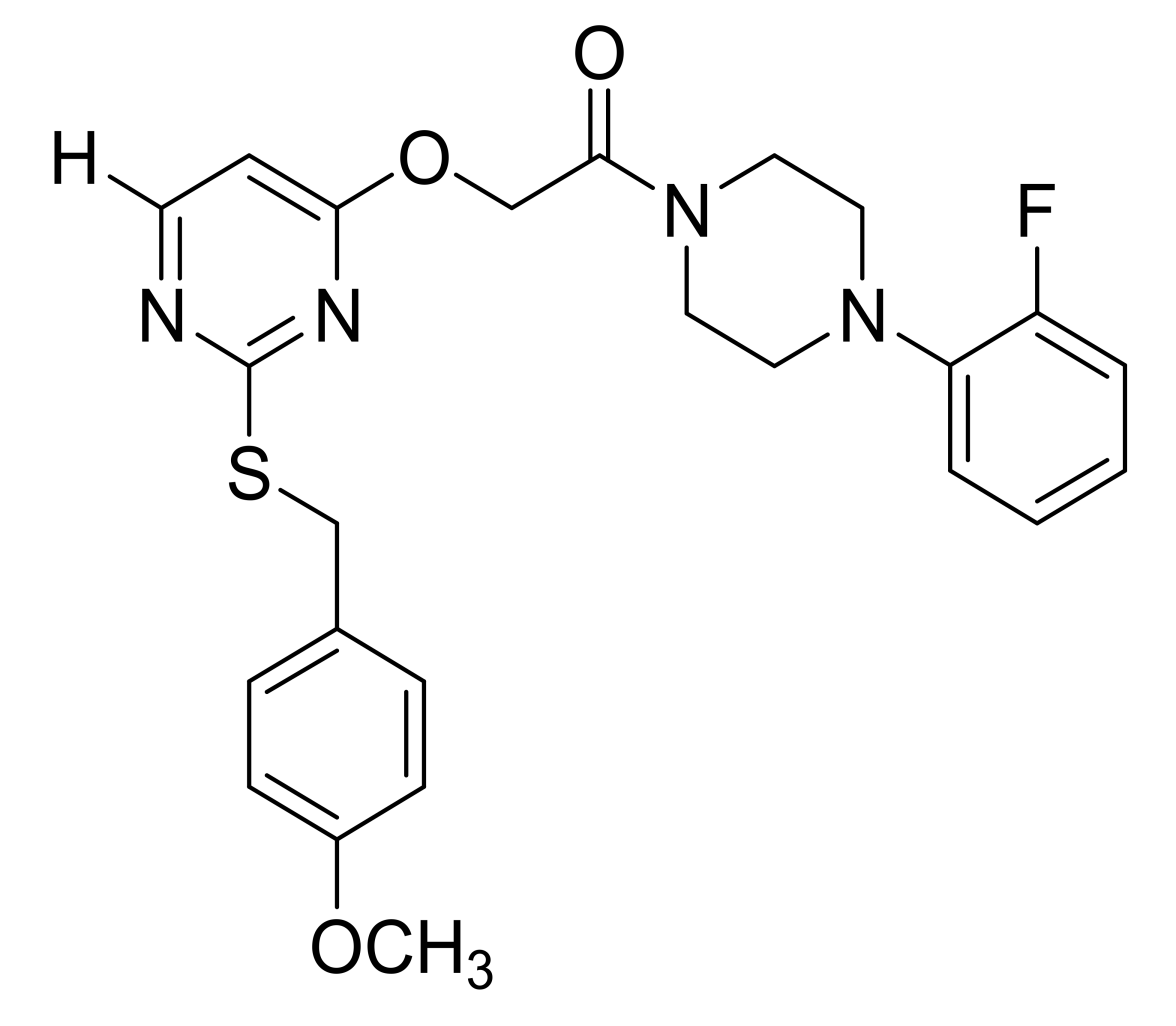	29.34
**5g**		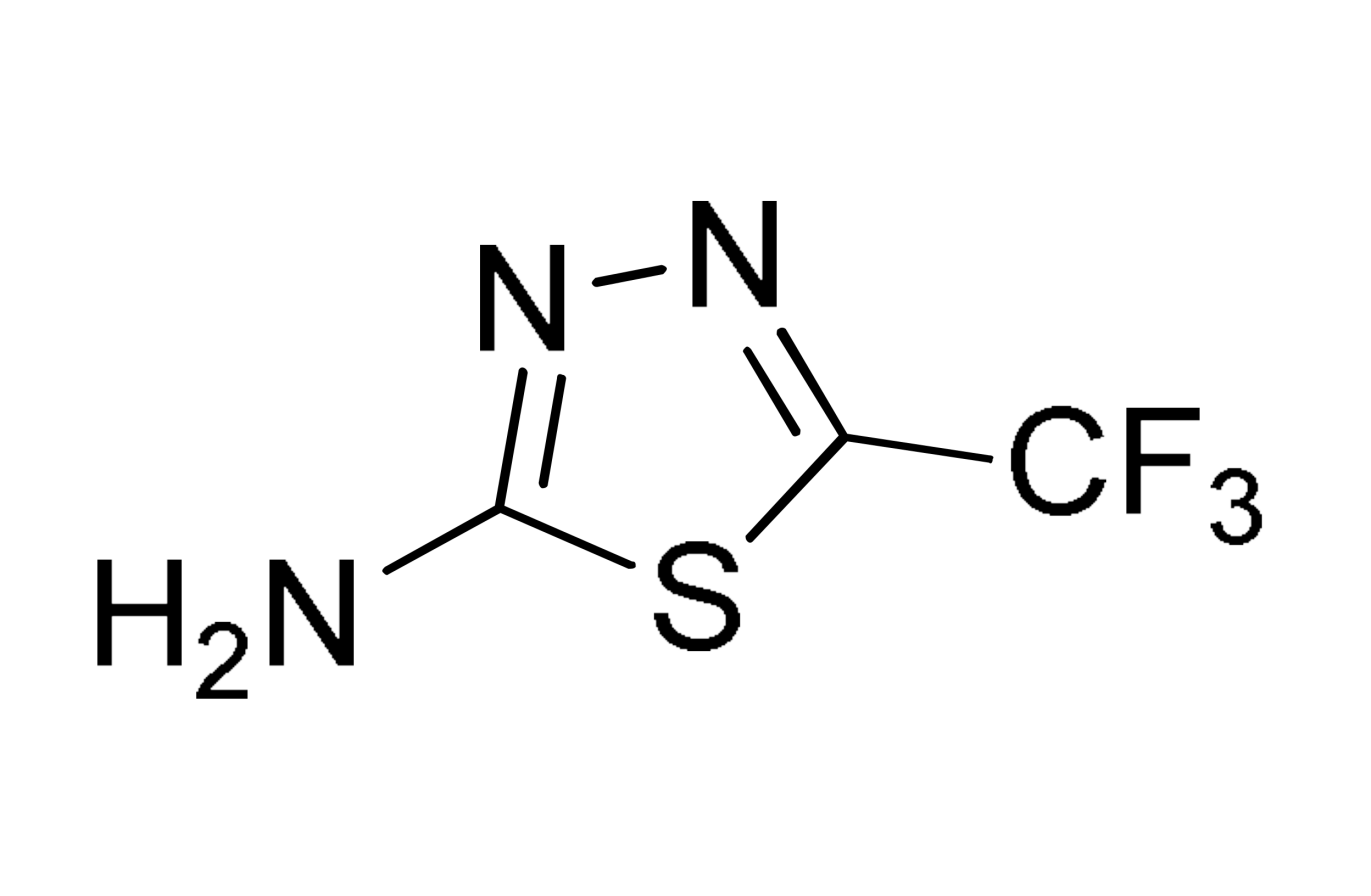	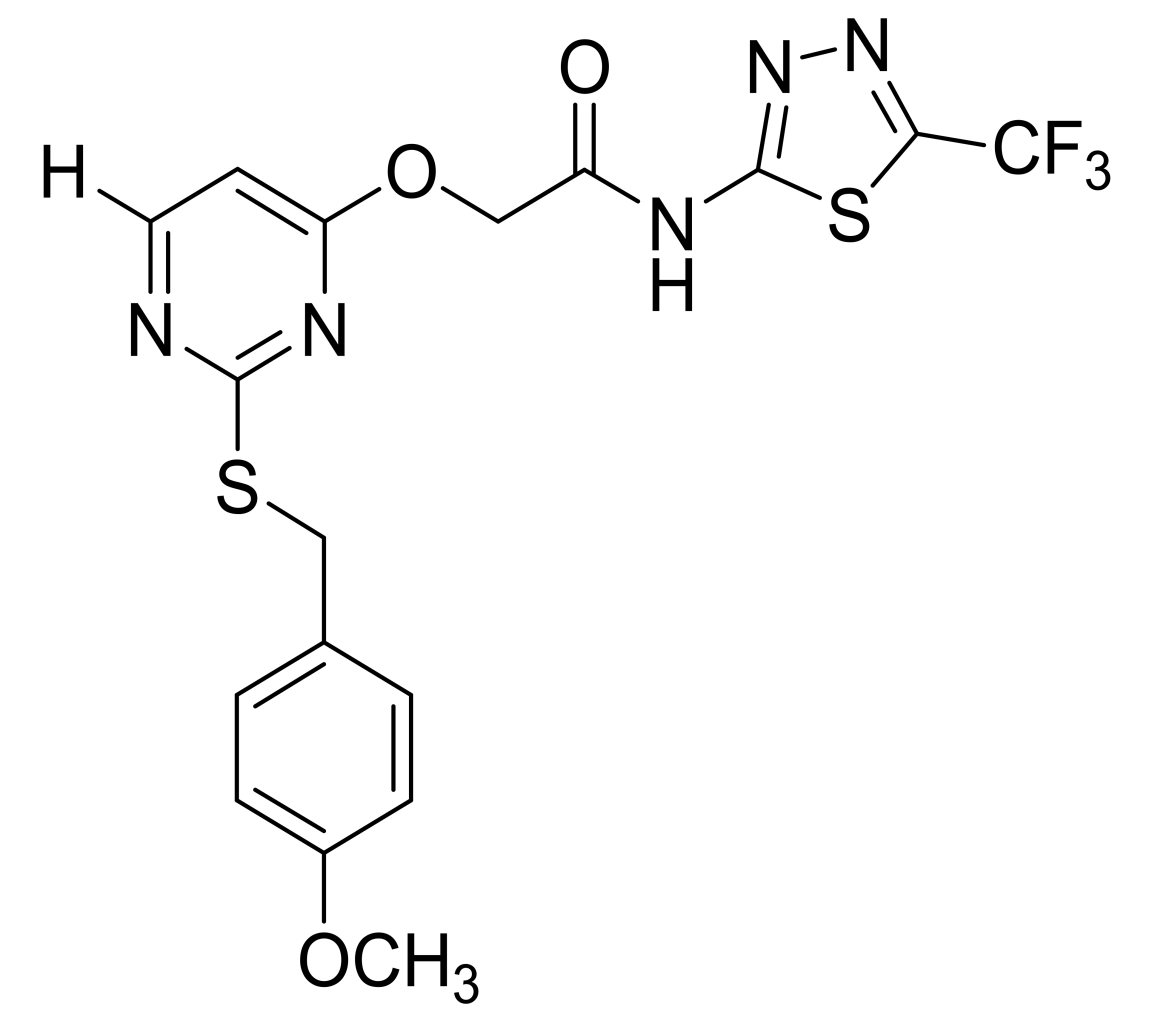	39.27
**5h**		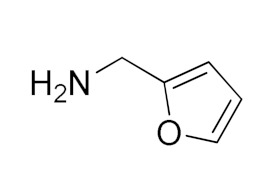	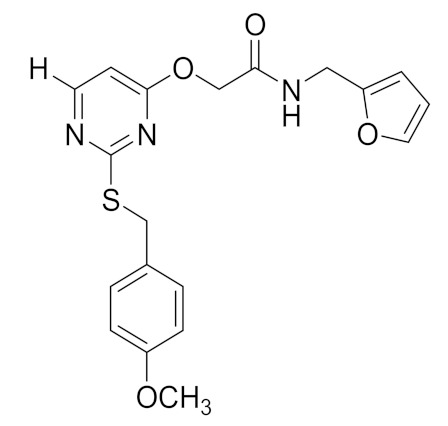	68.18
**5i**		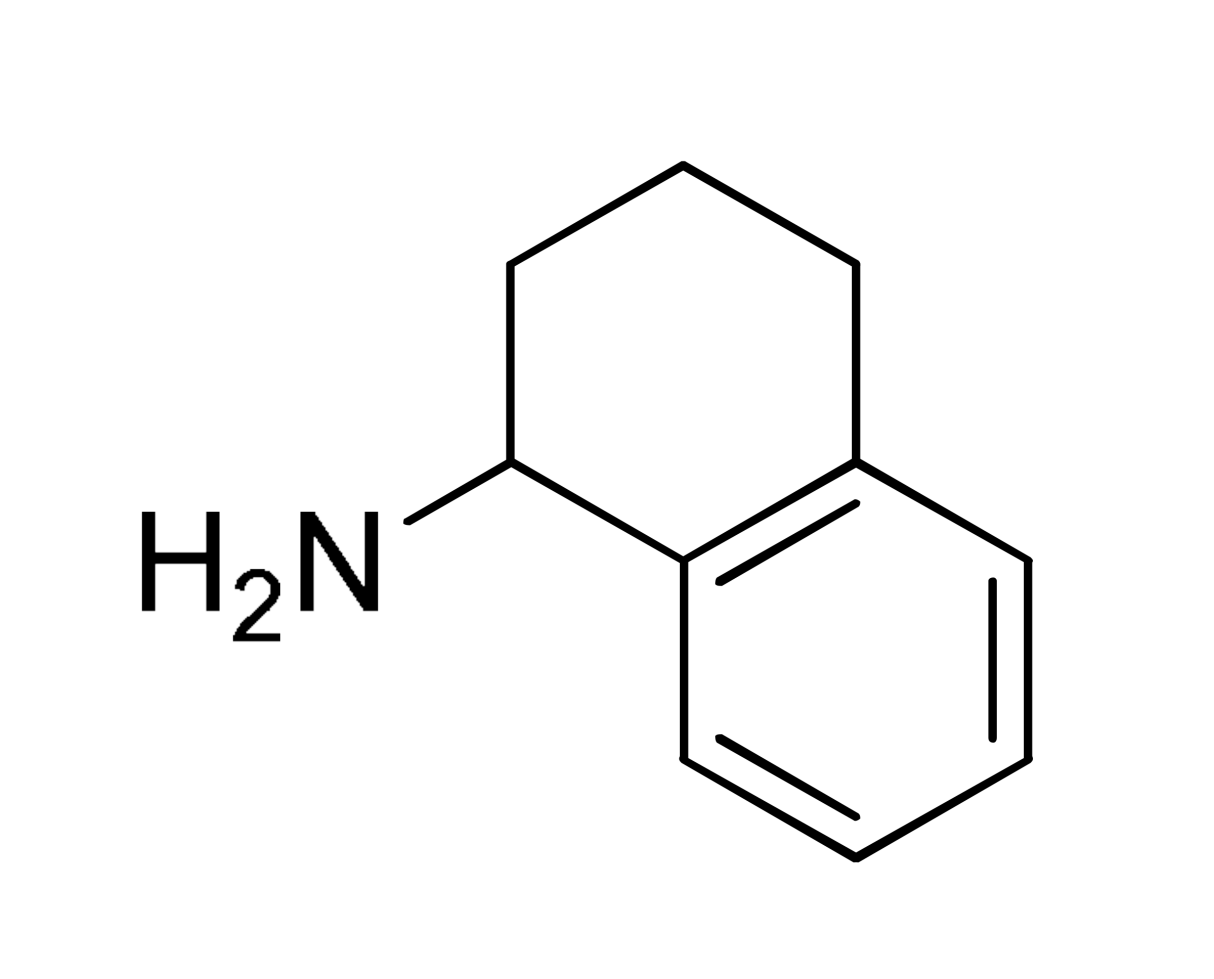	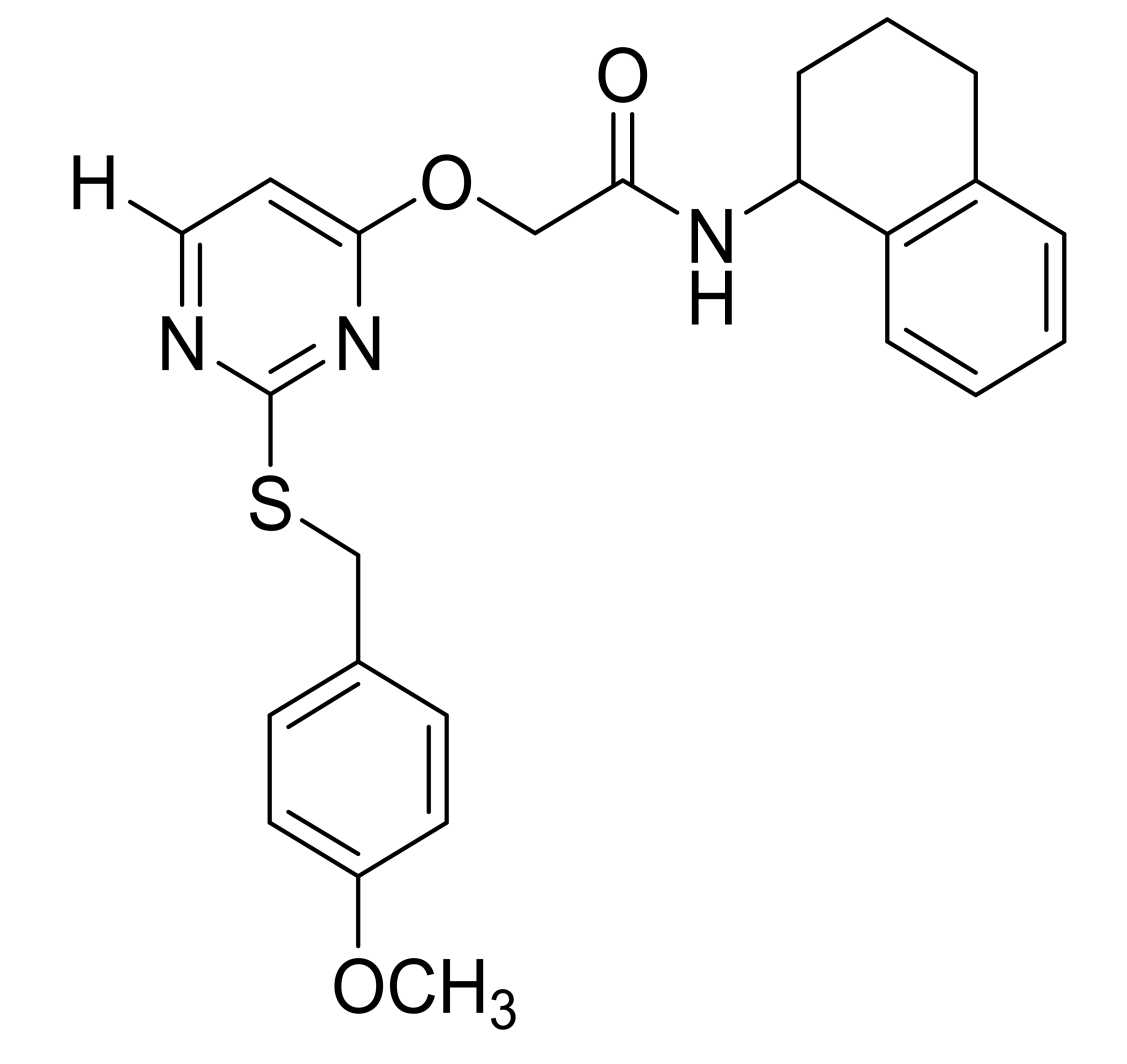	31.14
**5j**	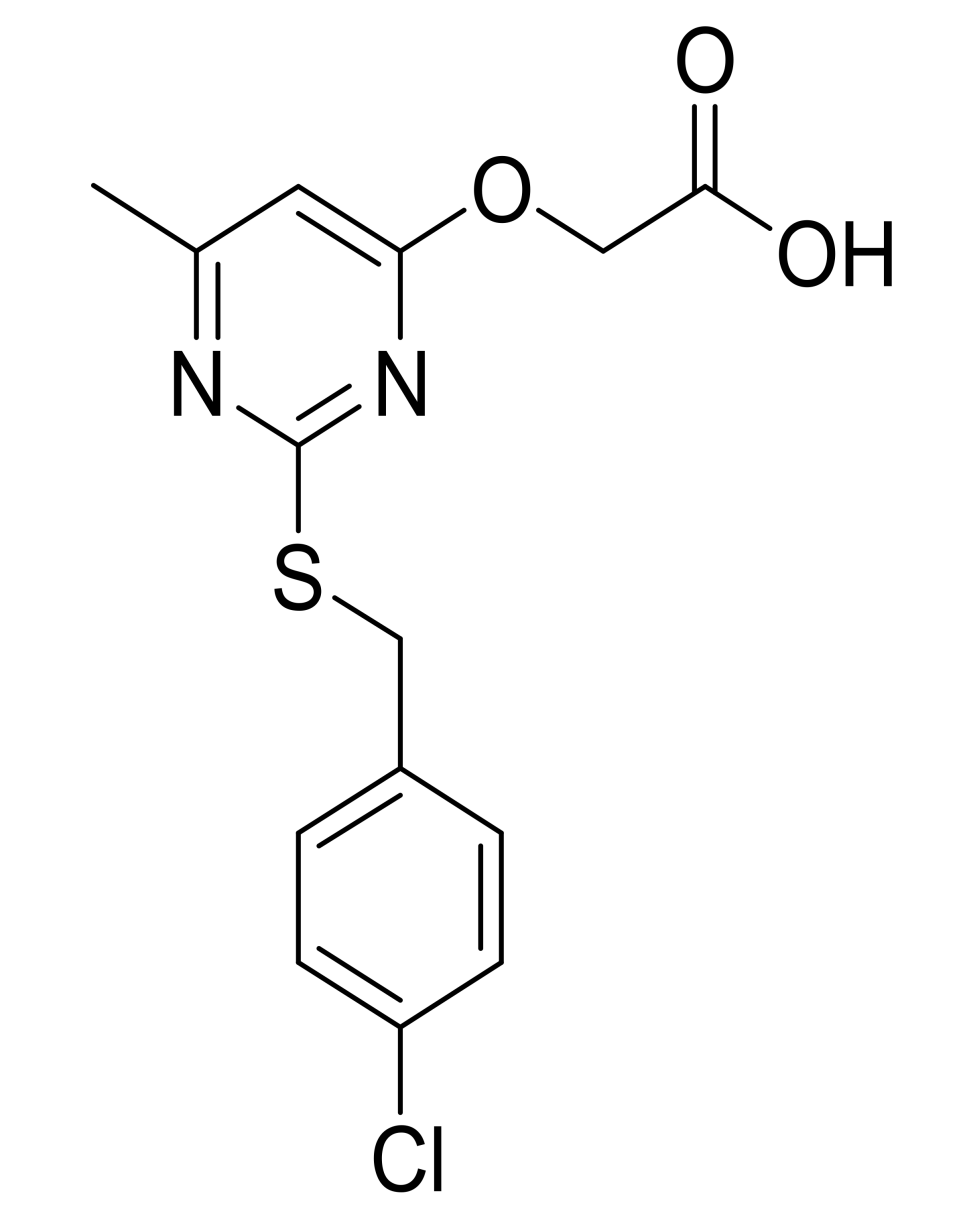	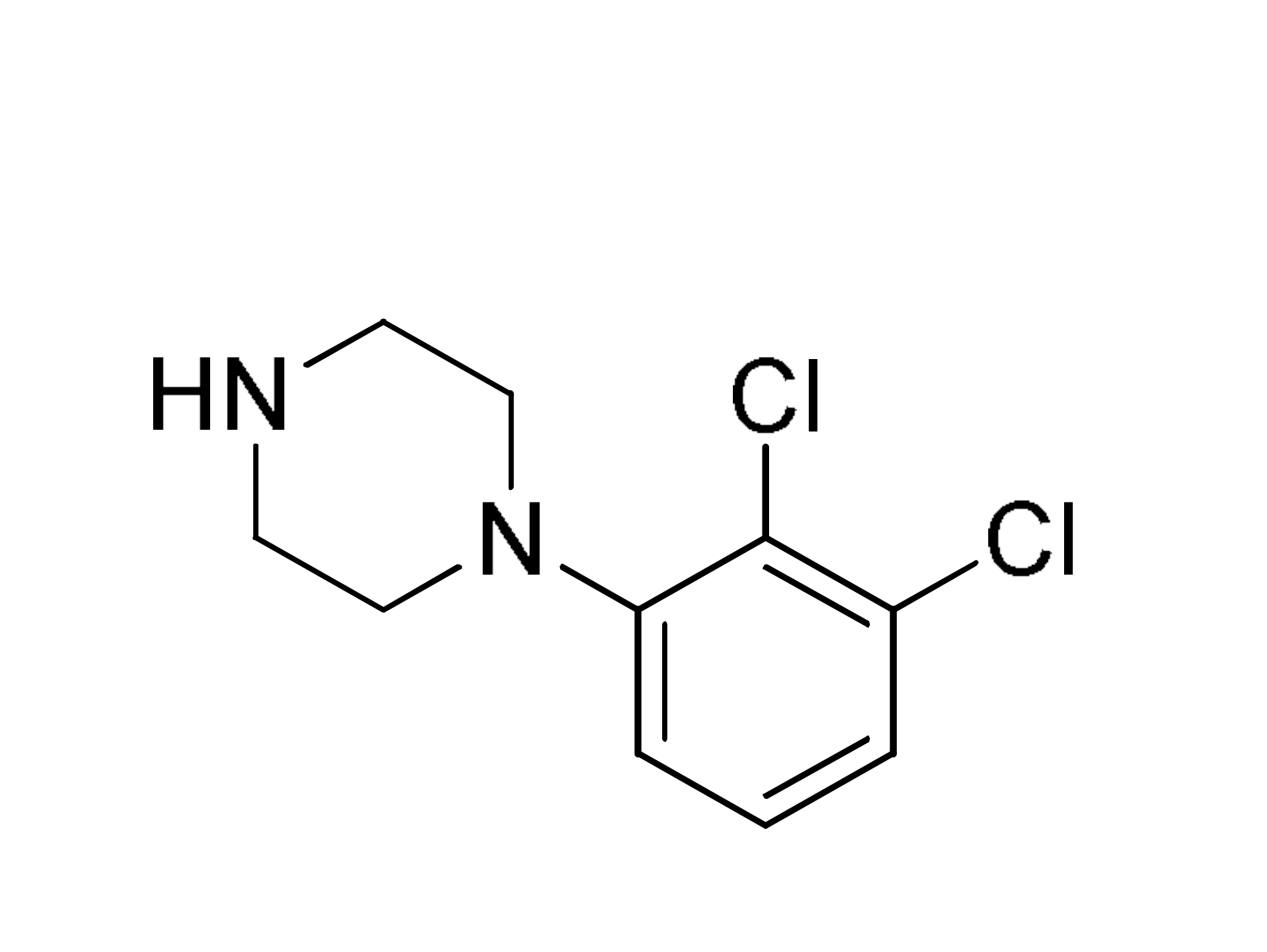	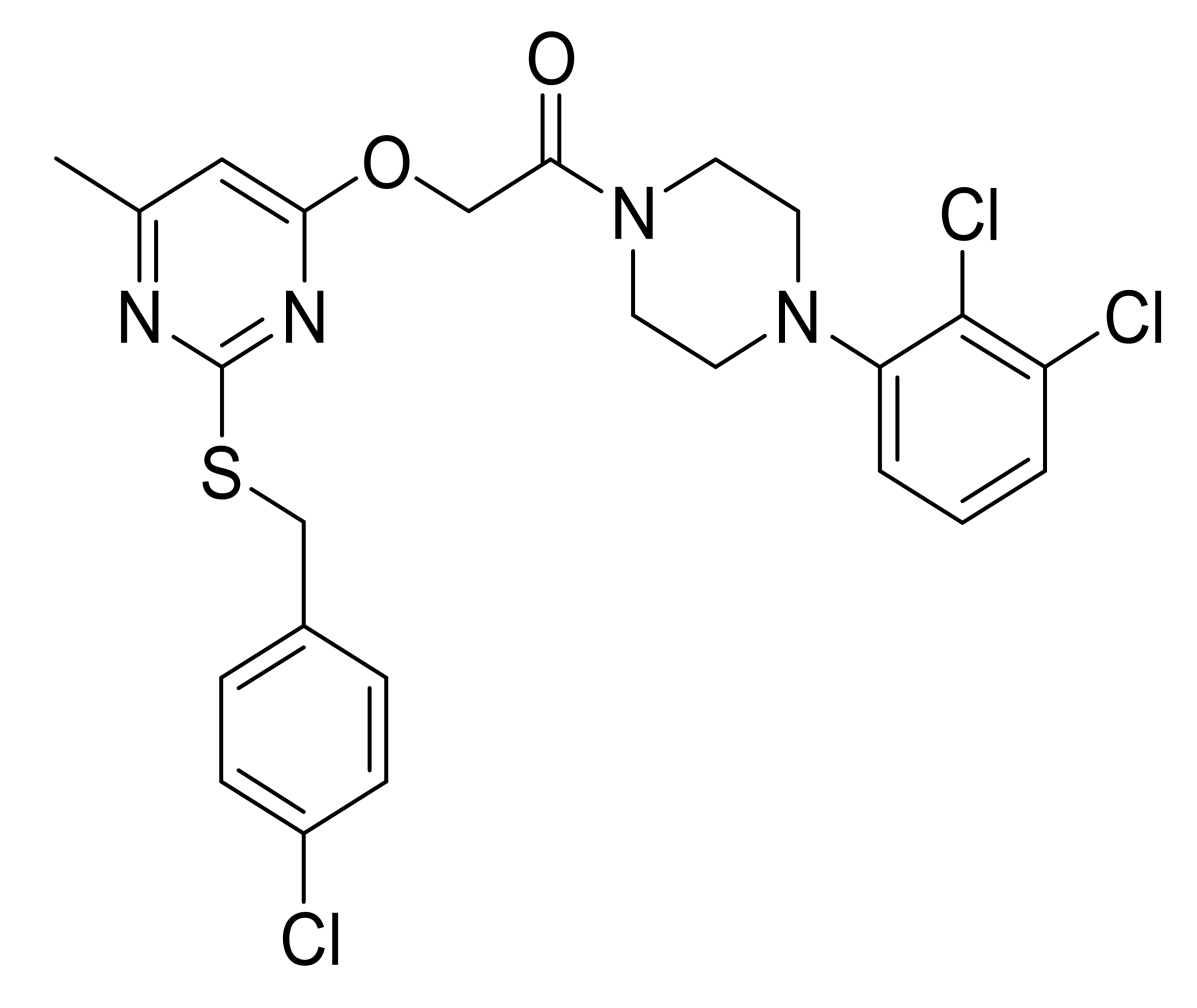	41.38
**5k**	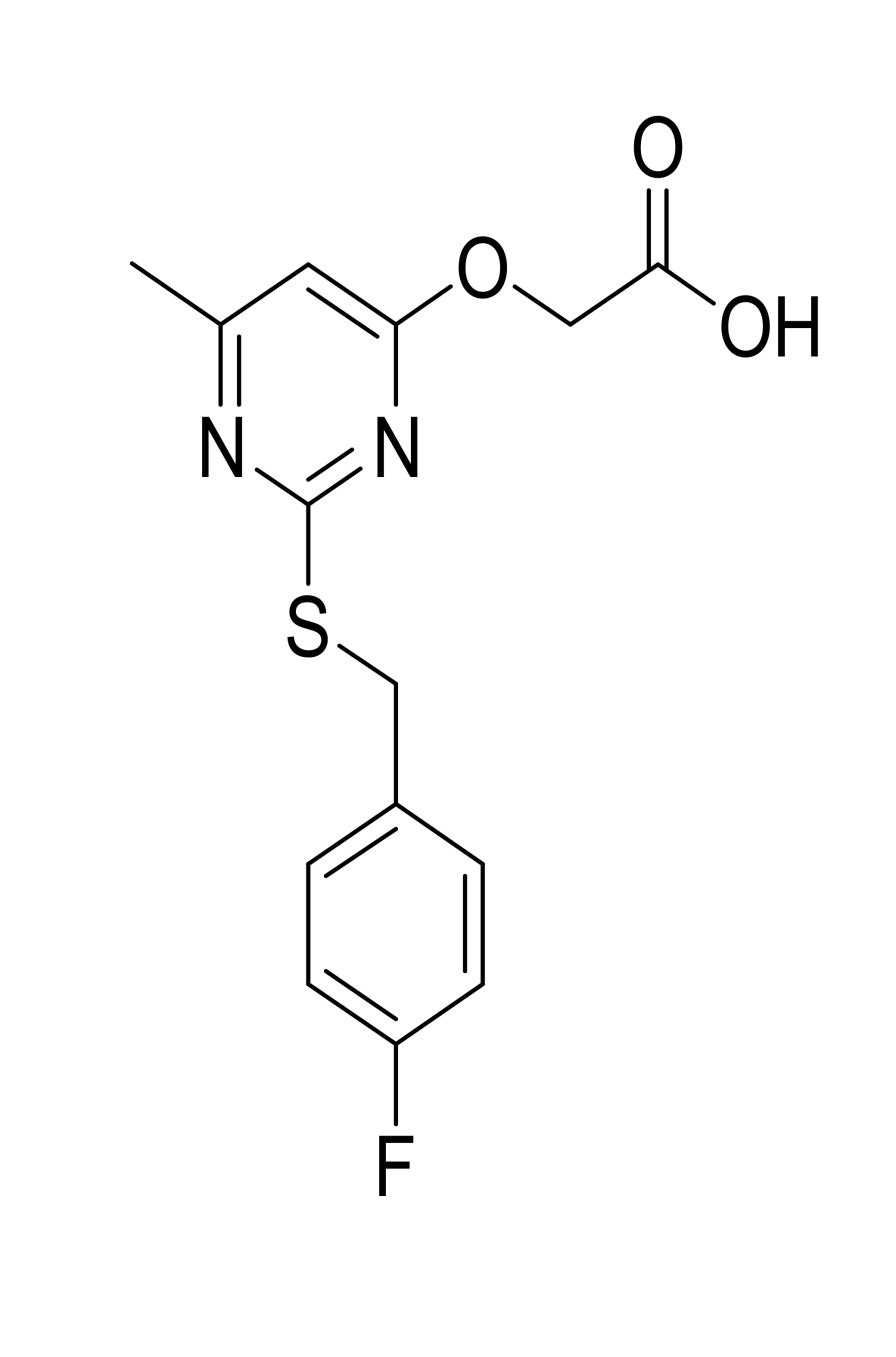	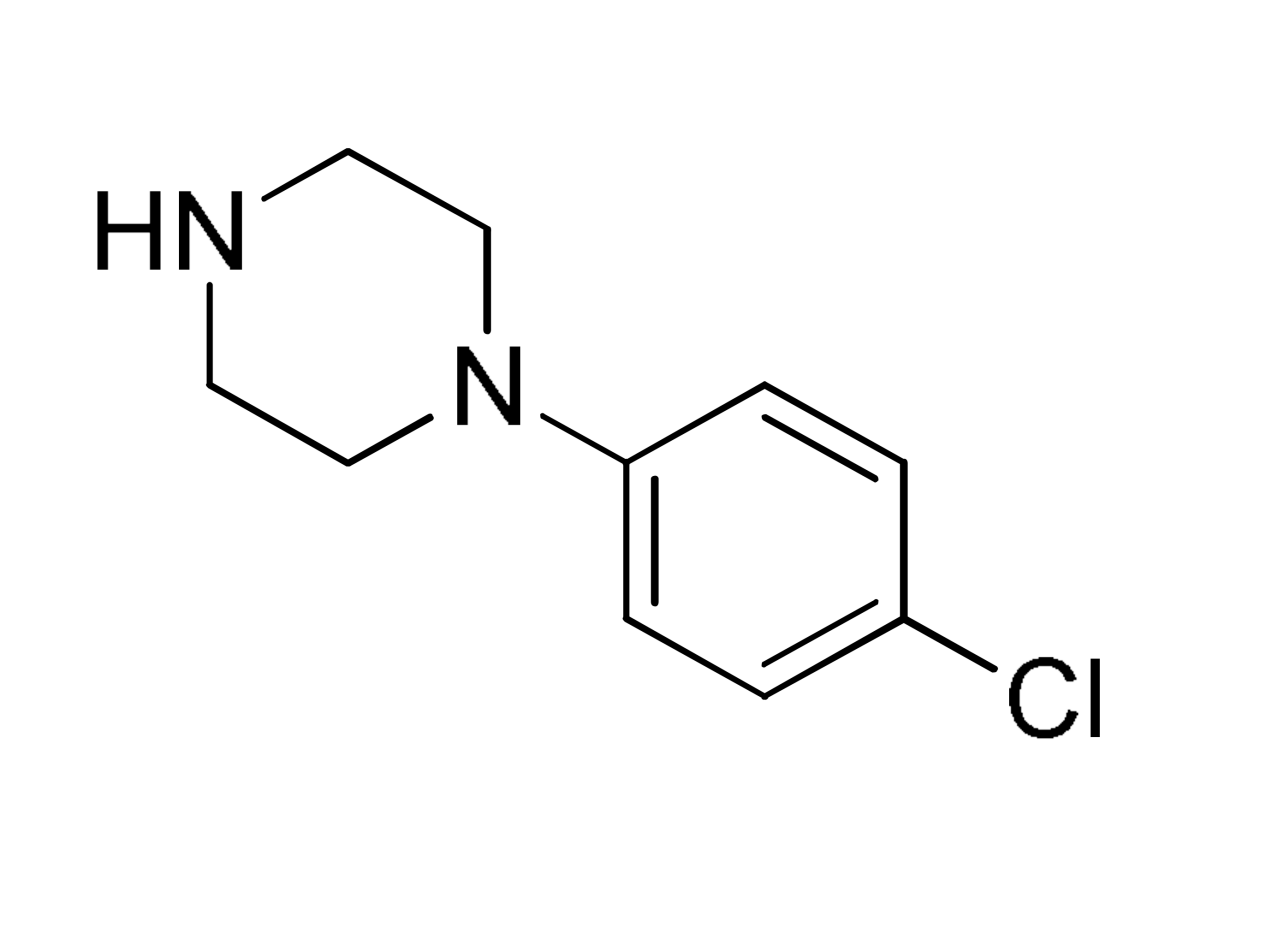	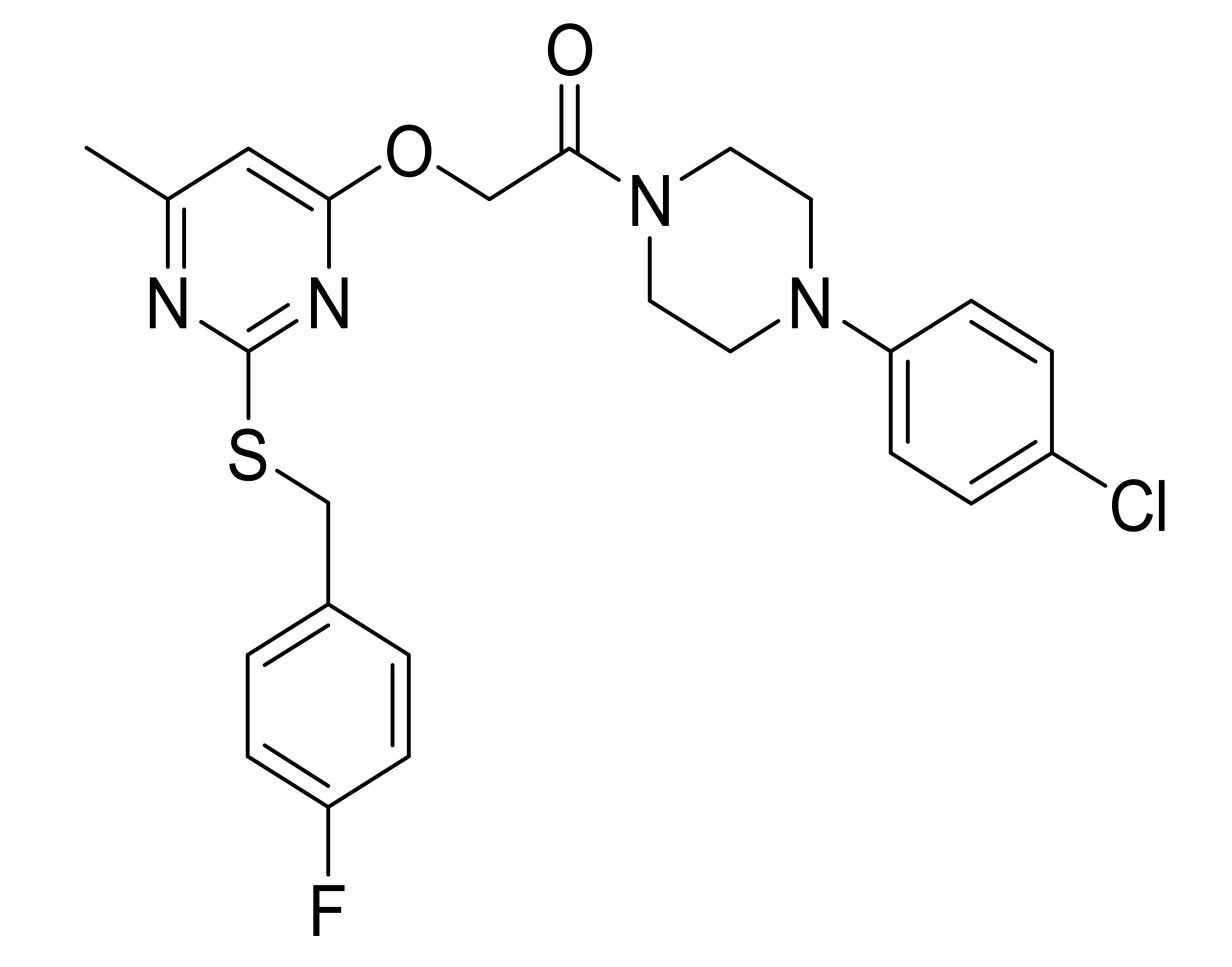	>100
**5l**		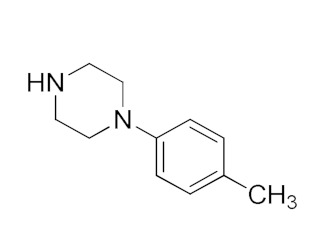	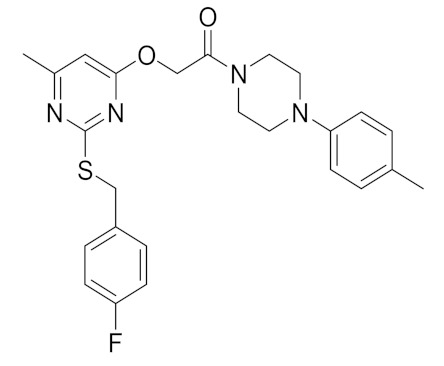	>100
Olaparib				**3.28**

**Table 2:** The calculated FMOs and global chemical reactivity descriptors.

Orbital Energy	DFT/B3LYP
E_HOMO_	−5.740 eV
E_LUMO_	−1.124 eV
ΔE_LUMO__−__HOMO_	4.615 eV
Ionization potential (I)	5.740 eV
Electron affinity (A)	1.124 eV
Hardness (η)	2.307 eV
Softness (S)	0.216 eV^−1^
Chemical potential (μ)	−3.432 eV
Electronegativity (χ)	3.432 eV
Electrophilicity (ψ)	2.553 eV

## Supplementary Materials

The following are available online at https://www.mdpi.com/article/10.3390/molecules27092848/s1, NMR spectra of Compounds **5a**–**5l**.

## Sample Availability

The synthesized compounds are available at Dr. Basappa Laboratory.
