# Supplementary material for: Development of 1-(4-(Substituted)piperazin-1-yl)-2-((2-((4-methoxybenzyl)thio)pyrimidin-4-yl)oxy)ethanones That Target Poly (ADP-Ribose) Polymerase in Human Breast Cancer Cells
Source: Molecules. 2022 Apr 29;27(9):2848. doi: 10.3390/molecules27092848 (PMC9100275; doi:10.3390/molecules27092848)
Supplement: Supplementary file 1 [file molecules-27-02848-s001.zip › molecules-1598313-supplementary.pdf]

Development of 1-(4-(substituted)piperazin-1-yl)-2-((2-((4-methoxybenzyl)thio)pyrimidin-4-yl)oxy)ethanones that target Poly(ADP -Ribose)Polymerase in human breast cancer cells

Suresha N Deveshegowda, Prashant K Metri, Rashmi Shivakumar, Ji-Rui Yang, Shobith Rangappa, Ananda Swamynayaka, Muthu K Shanmugam, Omantheswara Nagaraja, Mahendra Madegowda, Priya Babu Shubha, Arunachalam Chinnathambi, Sulaiman Ali Alharbi, Vijay Pandey, Kwang Seok Ahn, Peter E. Lobie and Basappa Basappa

Supplementary data for newly synthesized molecules and their IC50 values determined against human breast cancer cells

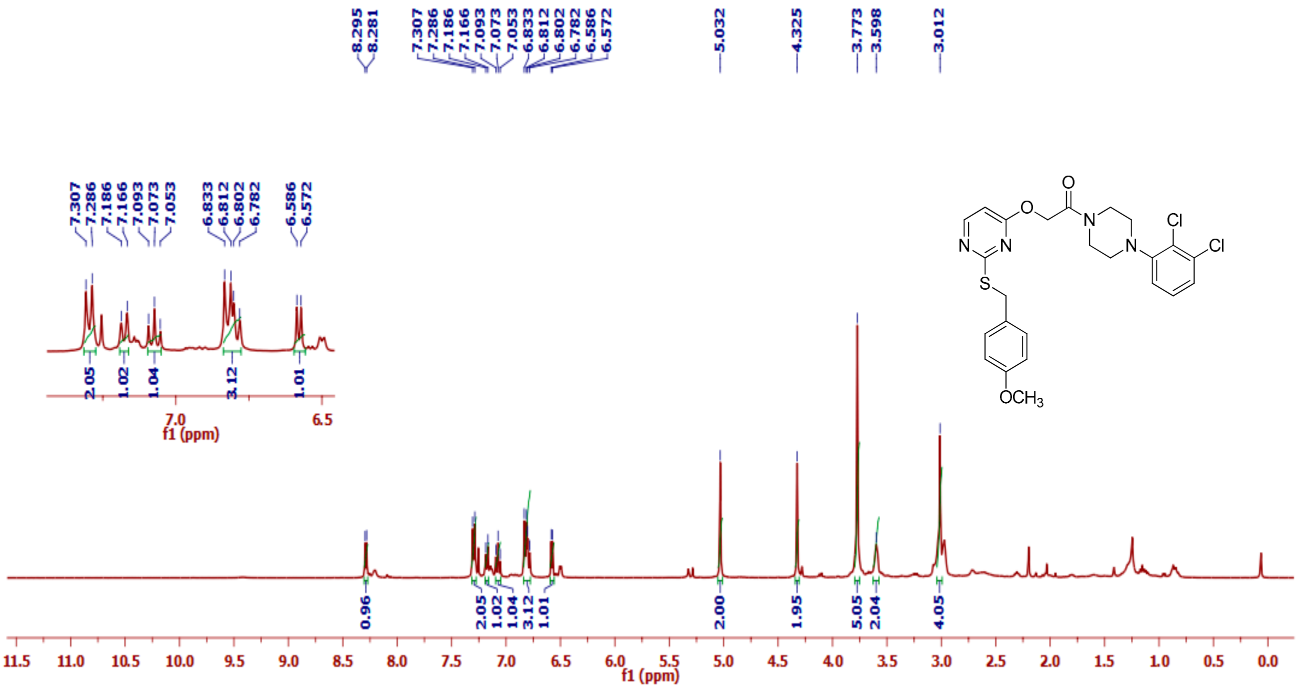

^{1}H NMR of compound **5a**

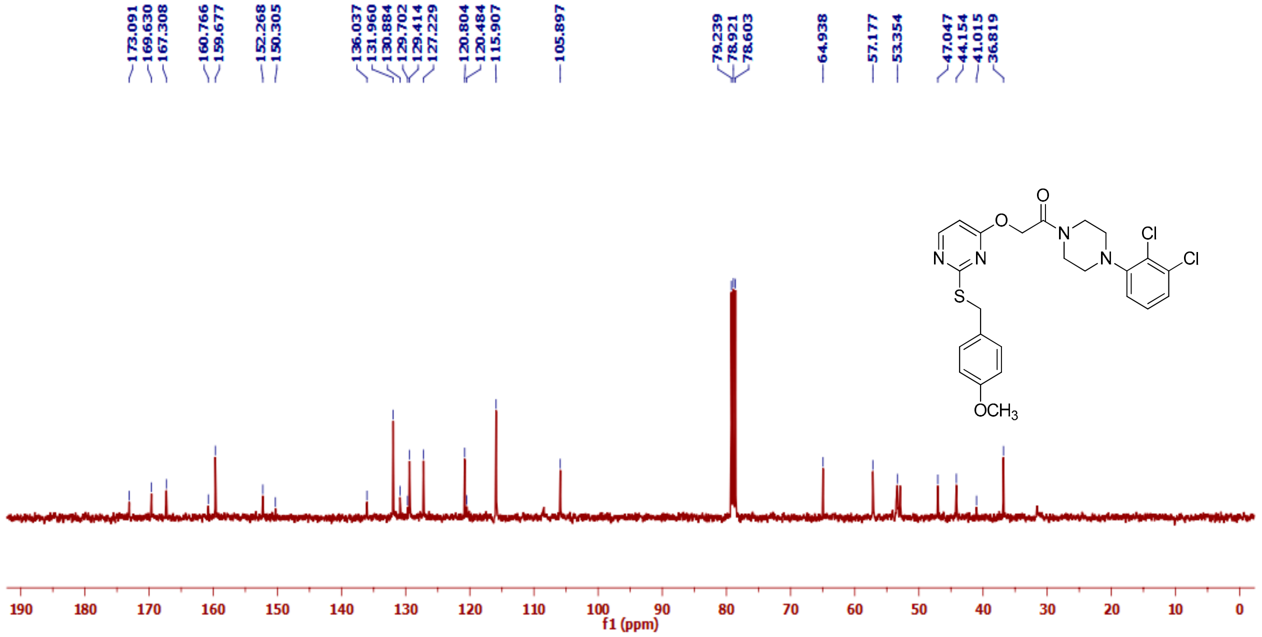

^{13}C NMR of compound **5a**

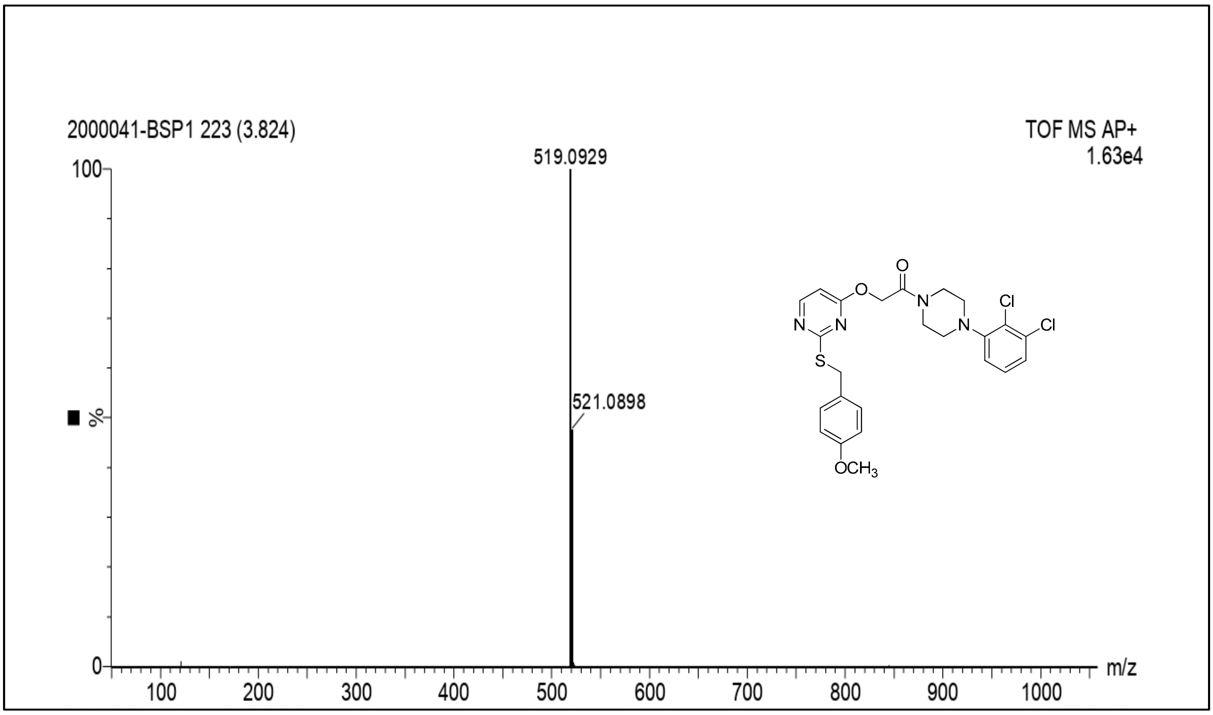

Mass spectra of **5a**

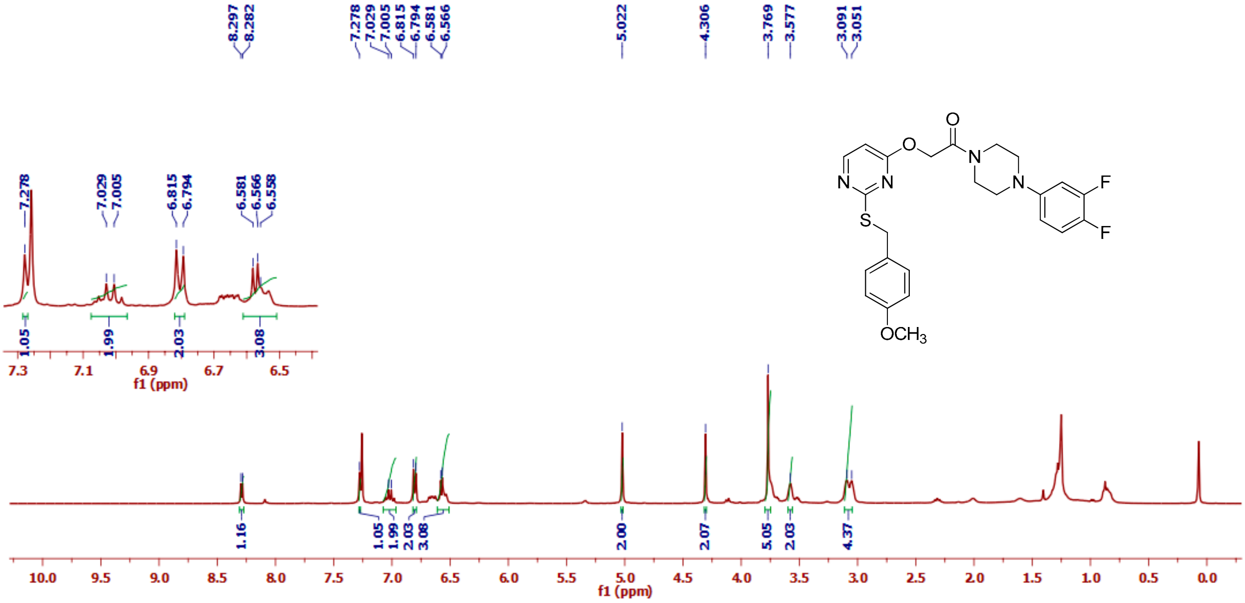

H NMR of compound **5b**

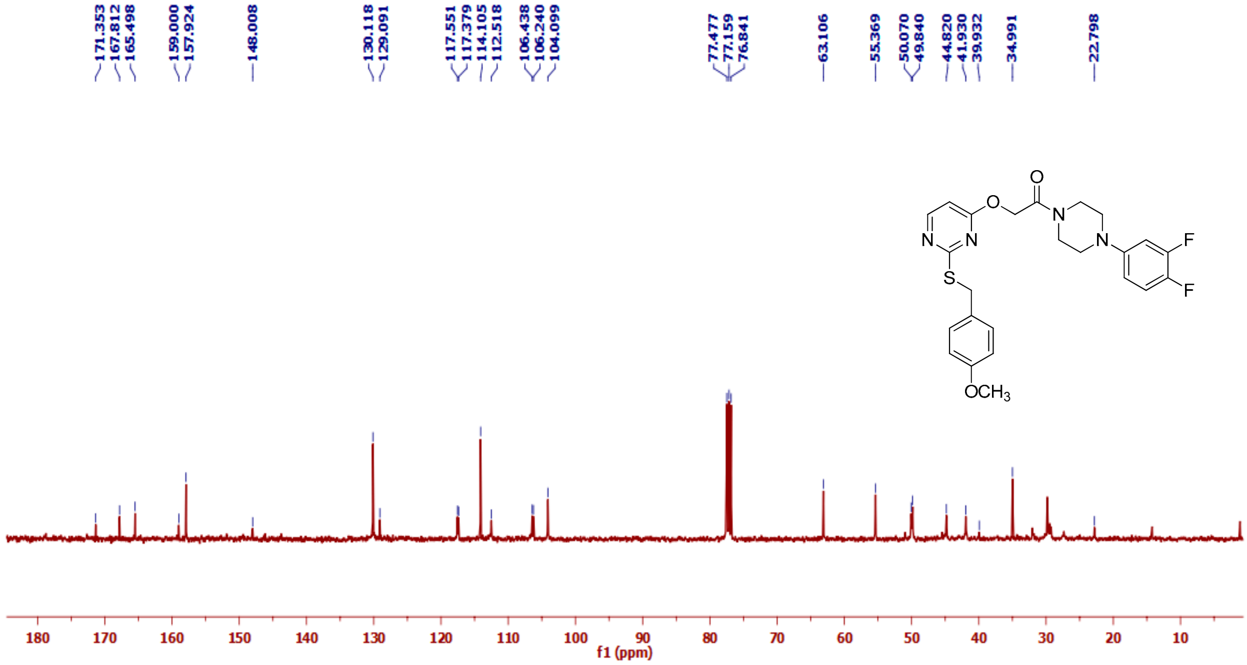

^{13}C NMR of compound **5b**

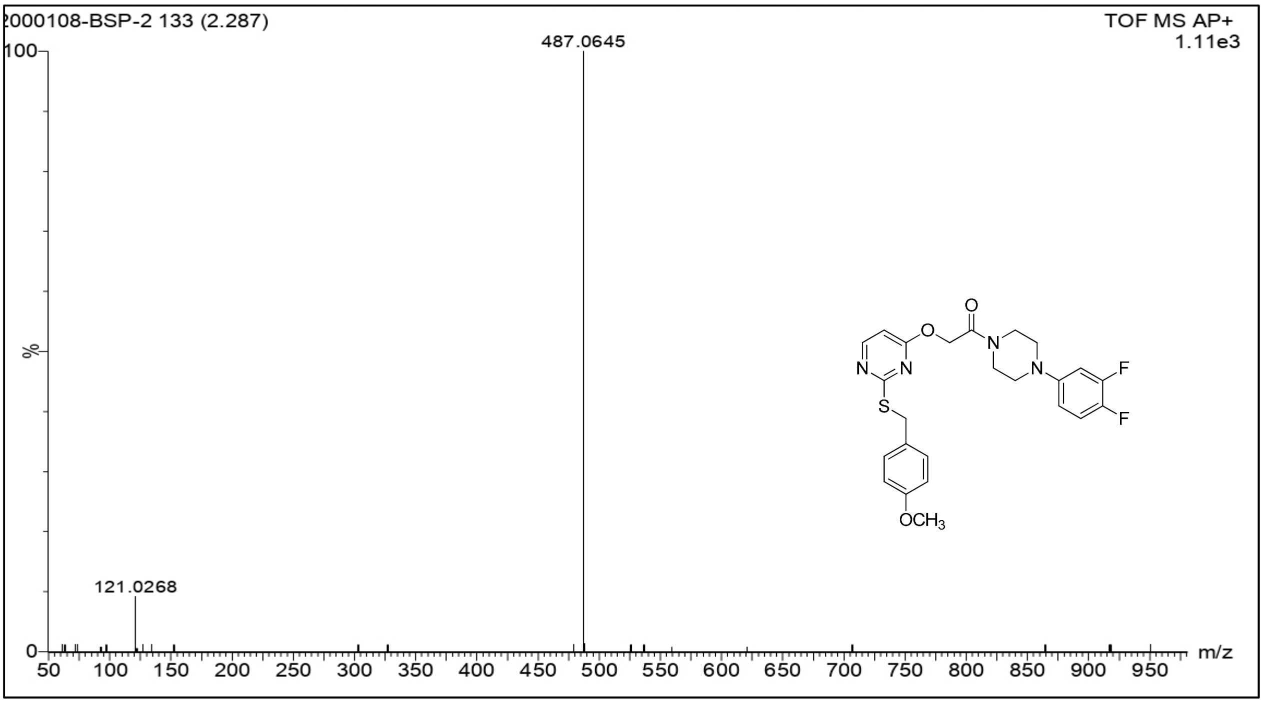

Mass spectra of **5b**

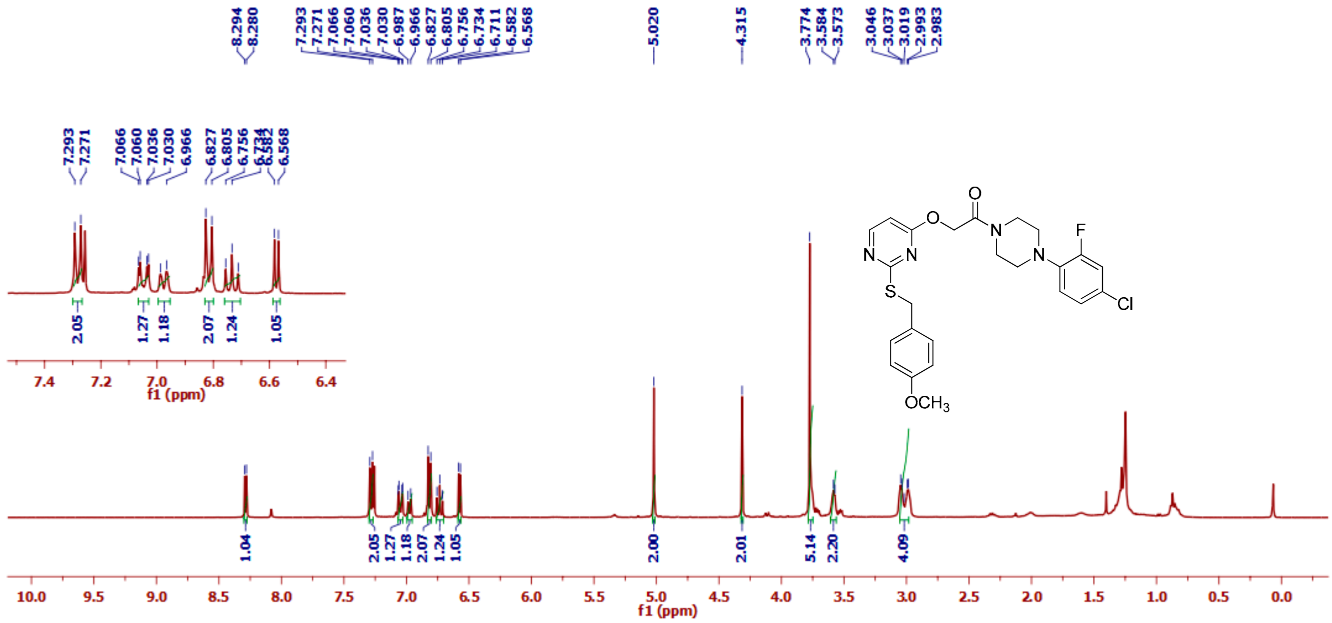

^{1}H NMR of compound **5c**

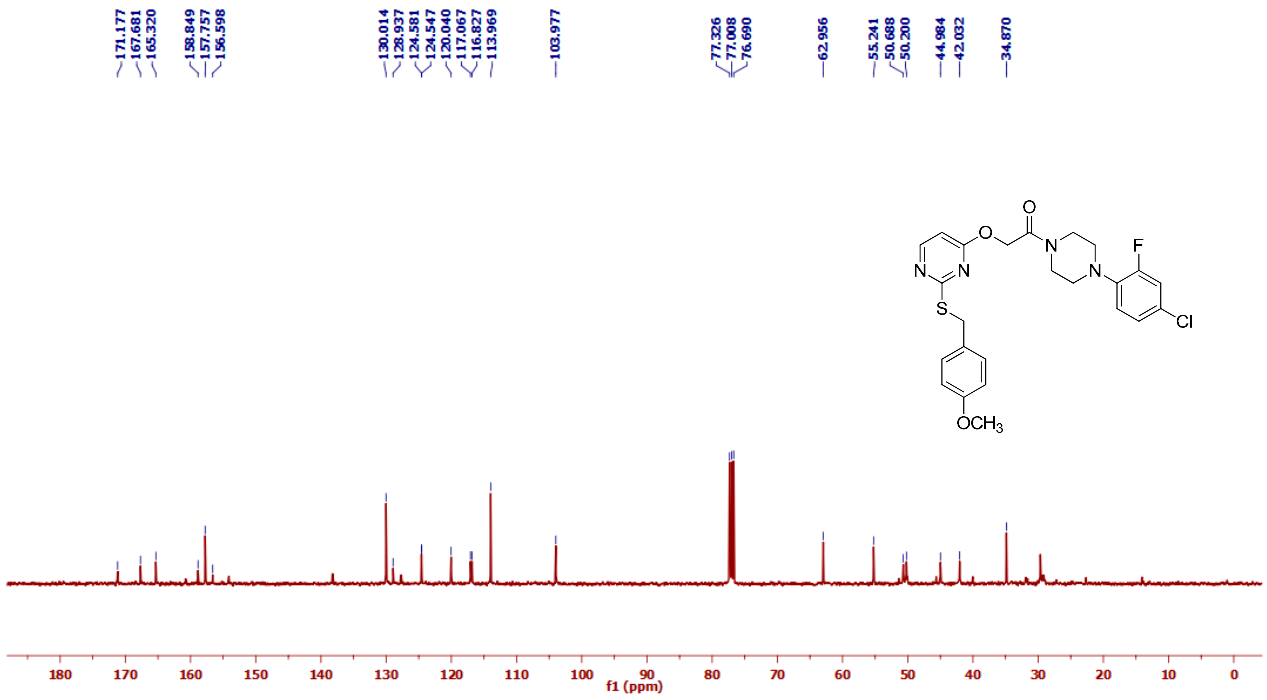

^{13}C NMR of compound **5c**

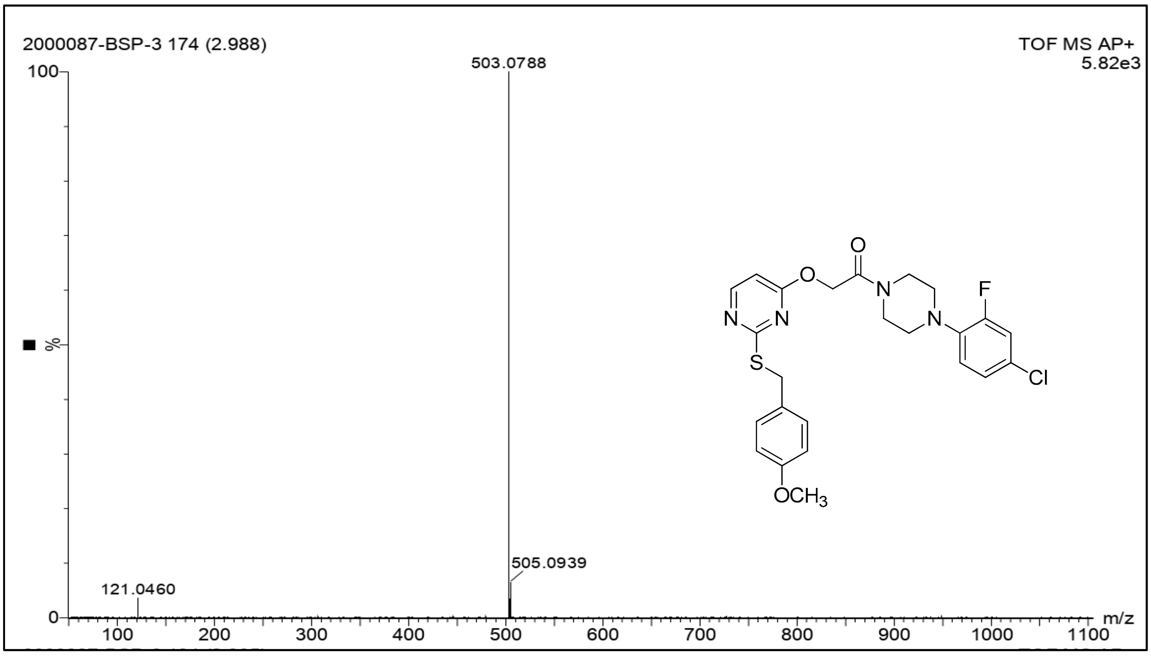

Mass spectra of **5c**

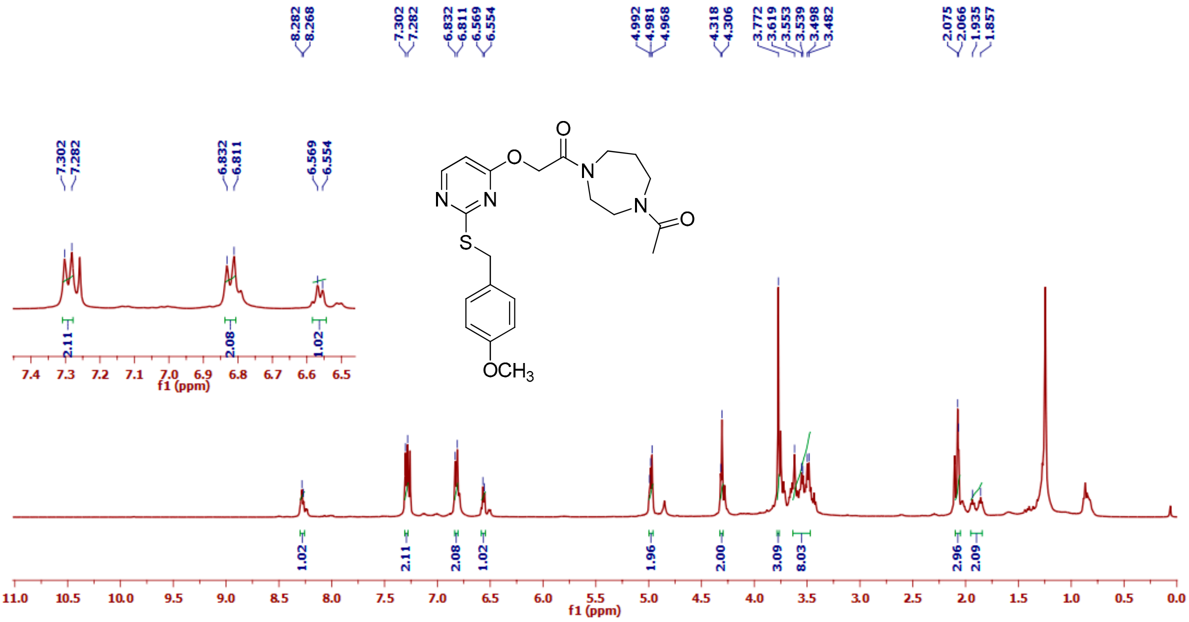

^{1}H NMR of compound **5d**

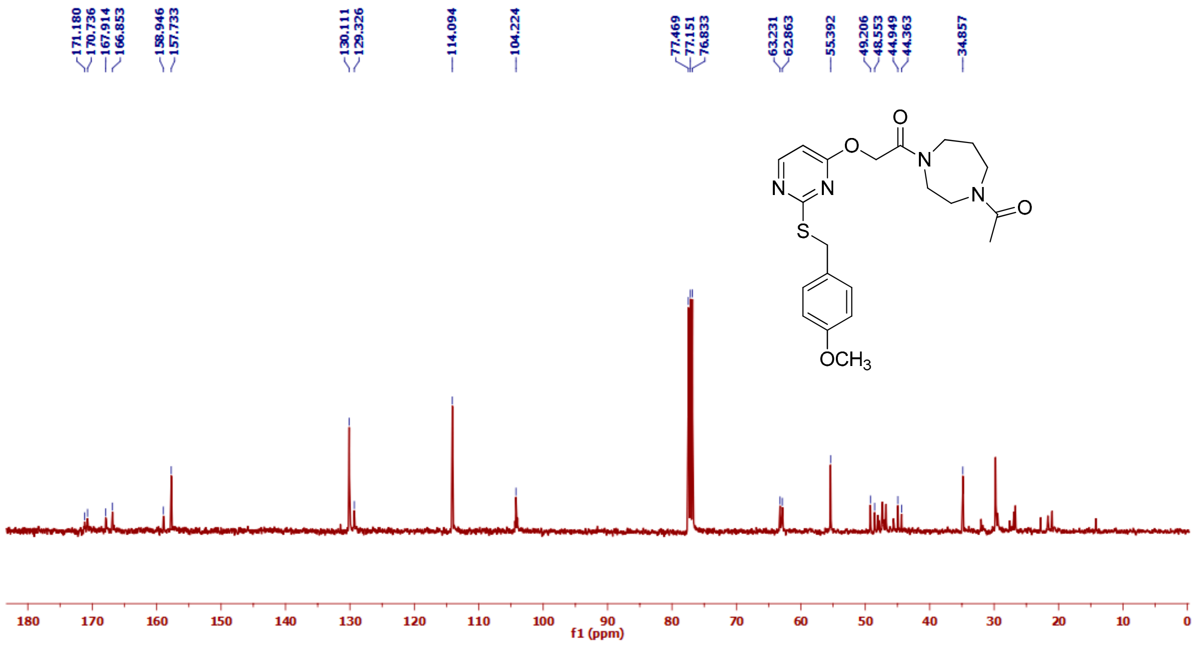

^{13}C NMR of compound **5d**

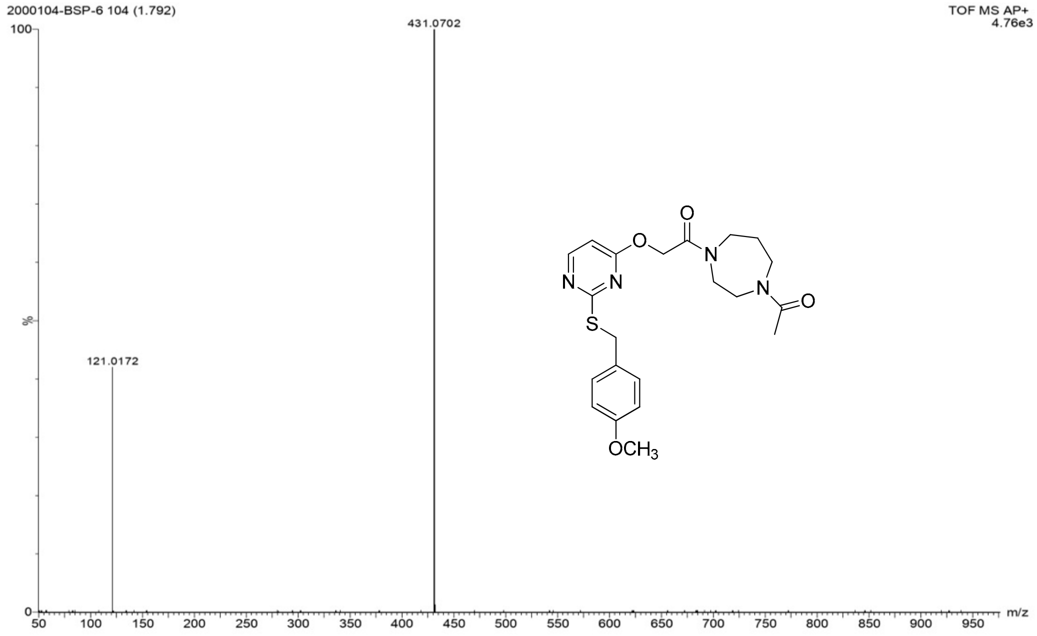

Mass spectra of **5d**

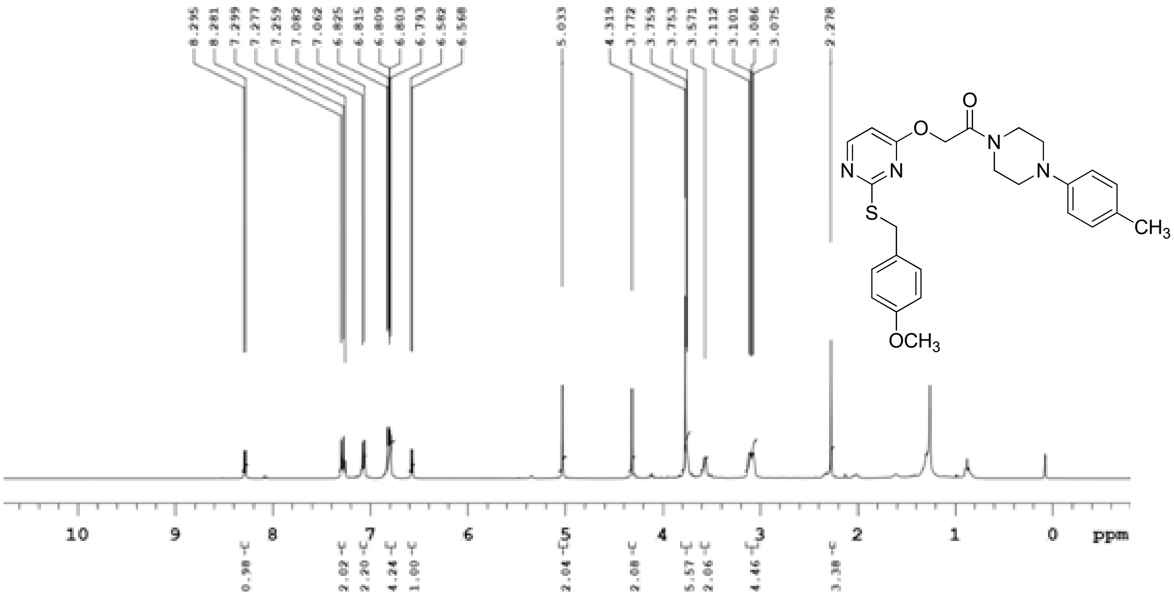

^{1}H NMR of compound **5e**

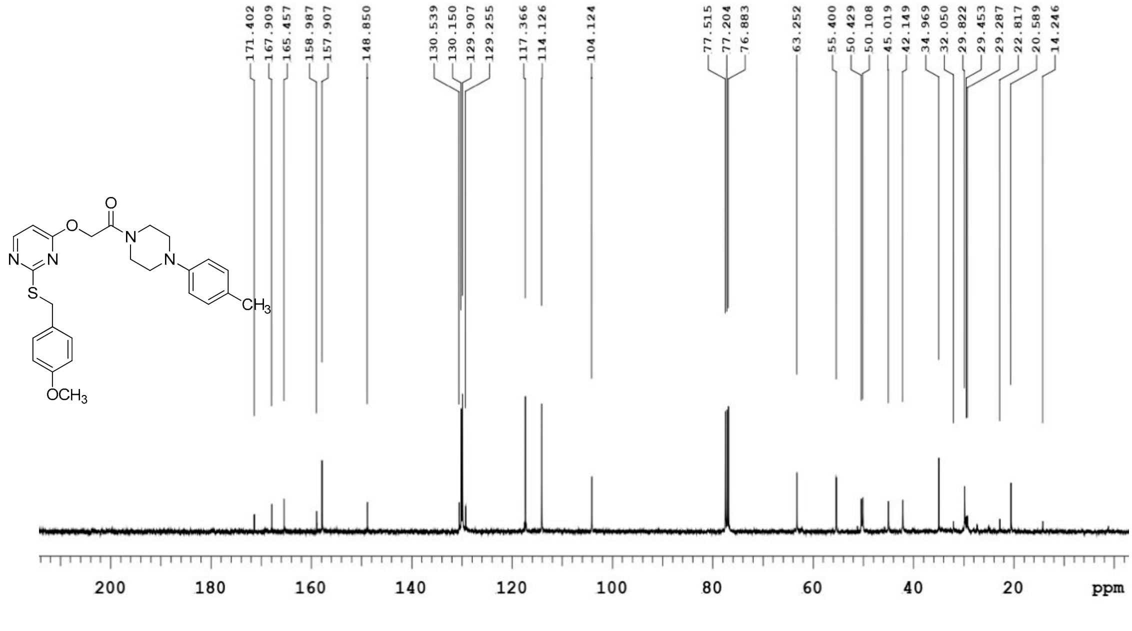

^{13}C NMR of compound **5e**

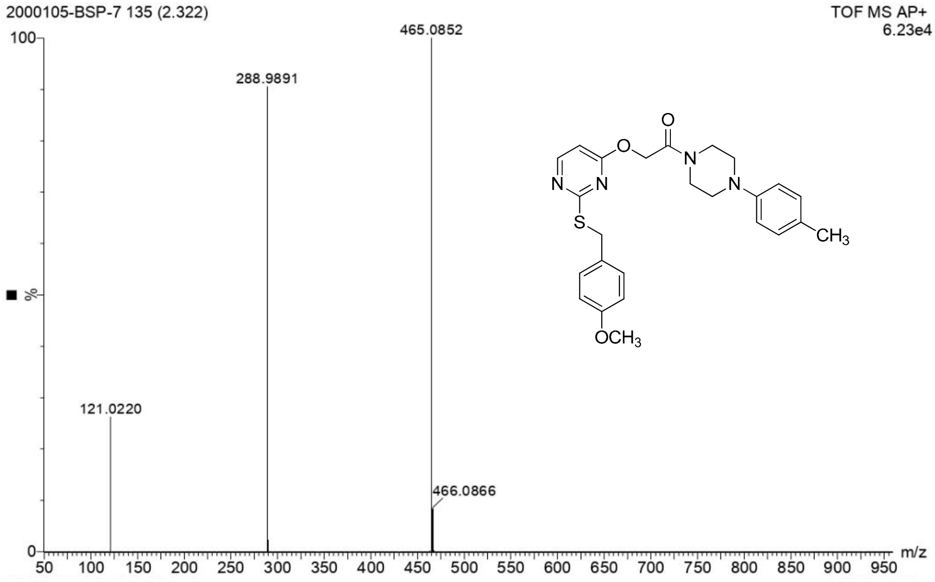

Mass spectra of 5e

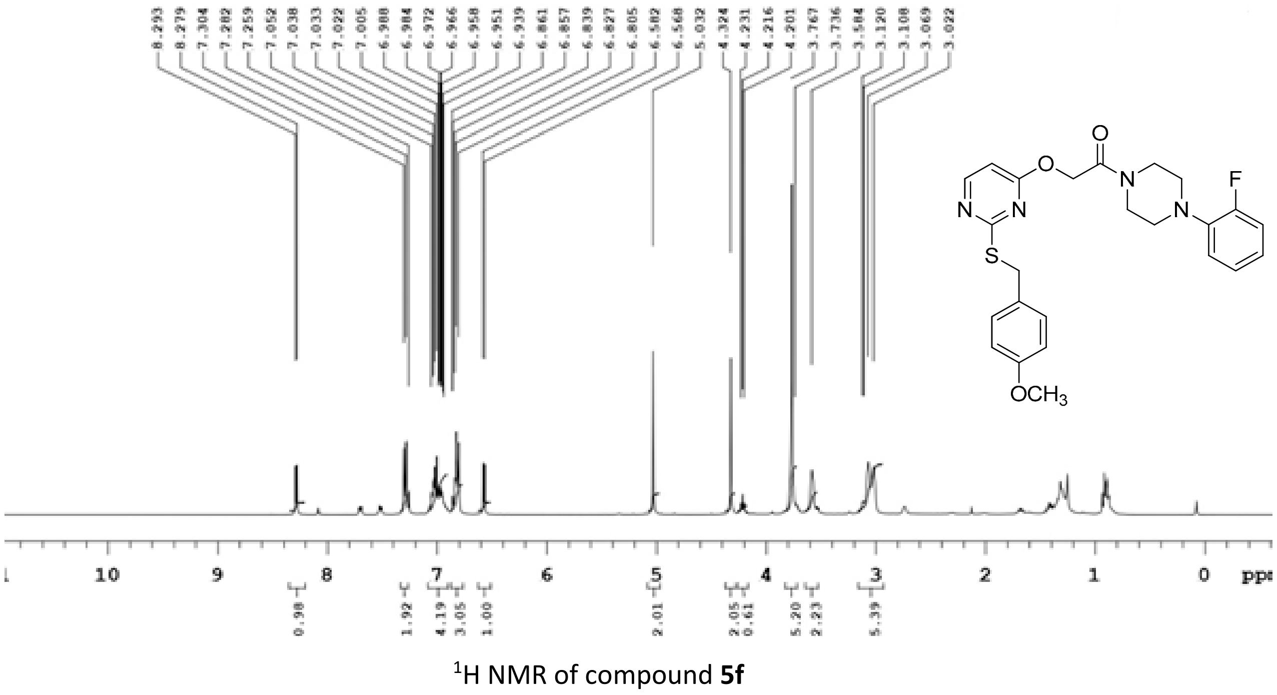

^{1}H NMR of compound **5f**

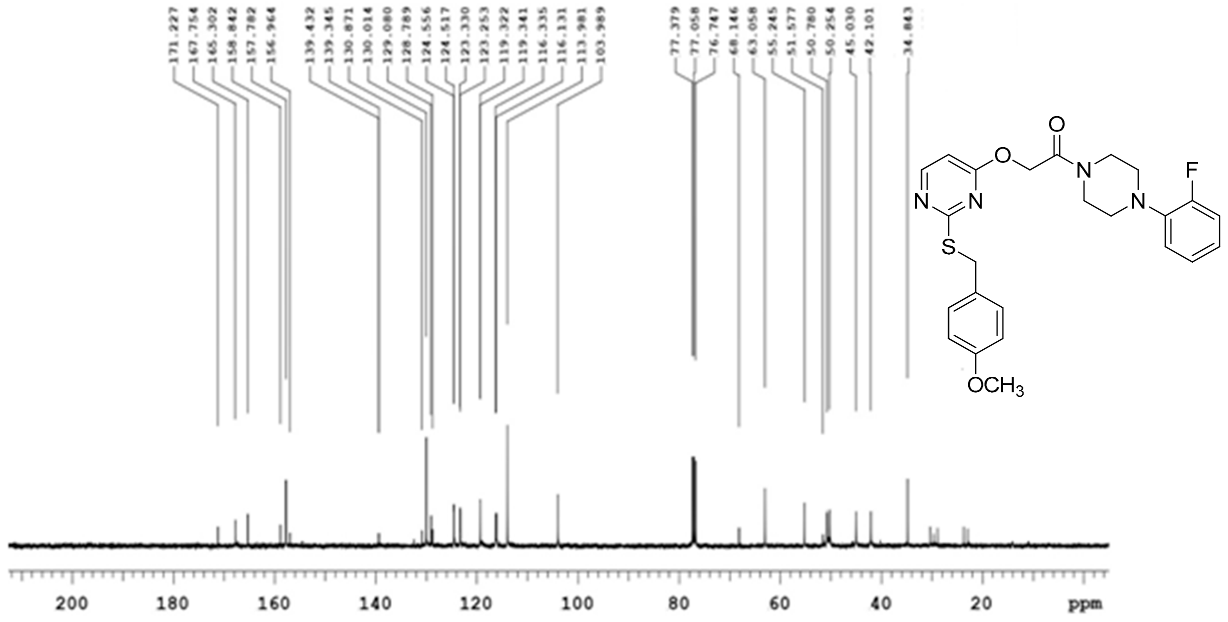

^{13}C NMR of compound **5f**

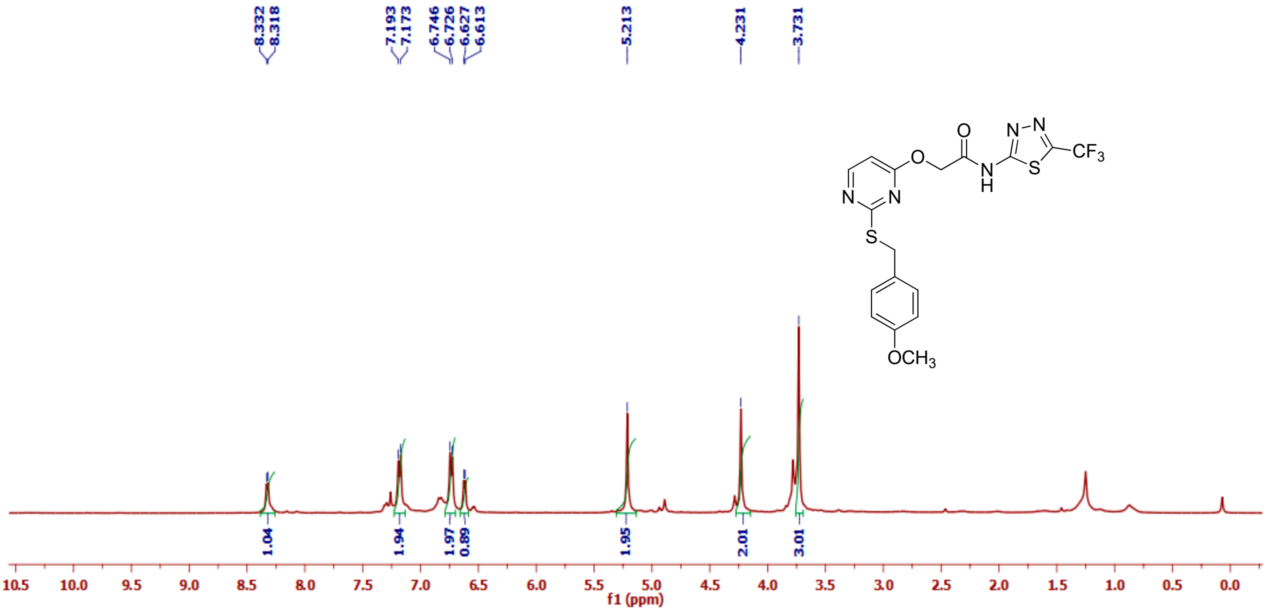

^{1}H NMR of compound **5g**

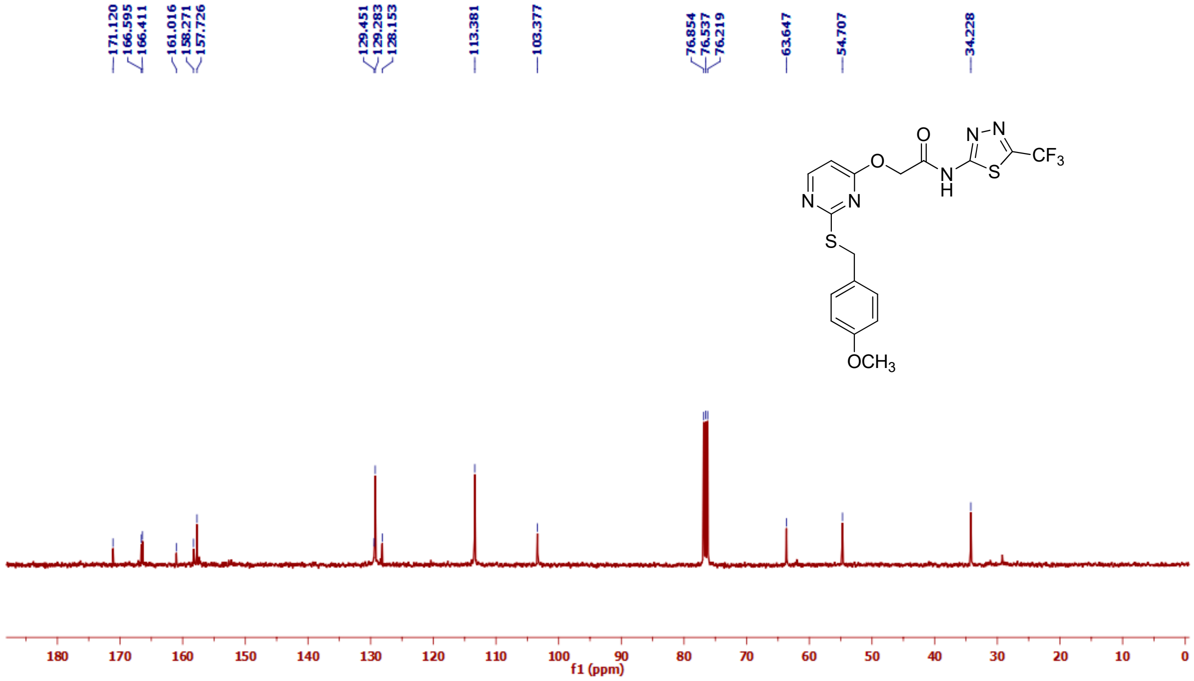

^{13}C NMR of compound **5g**

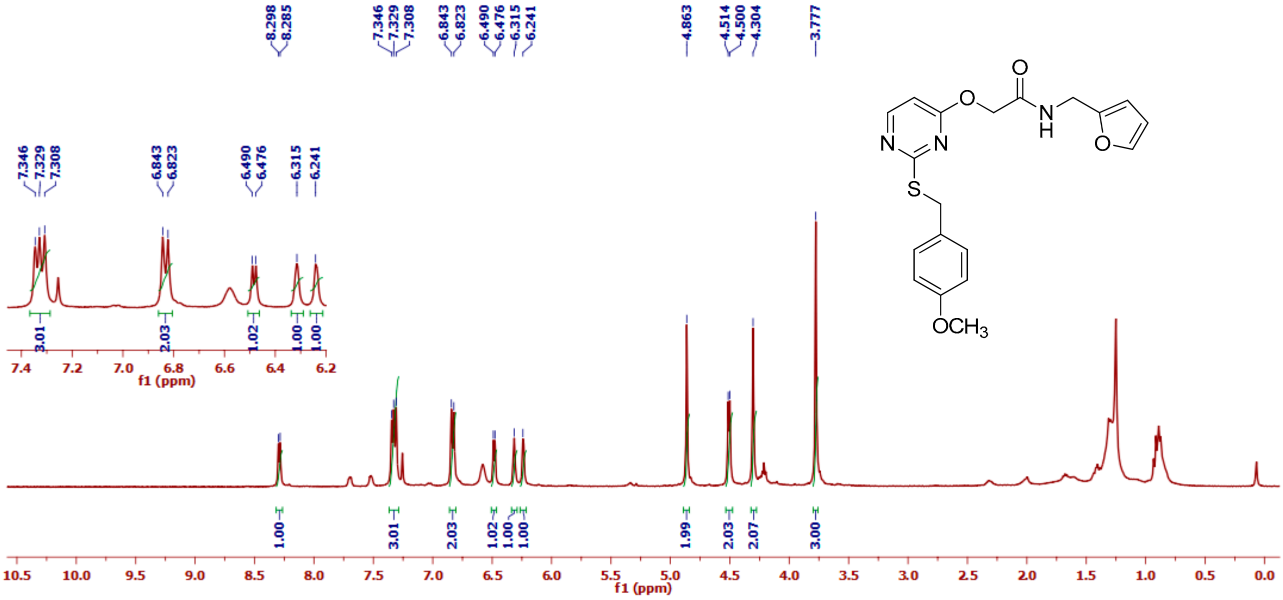

^{1}H NMR of compound **5h**

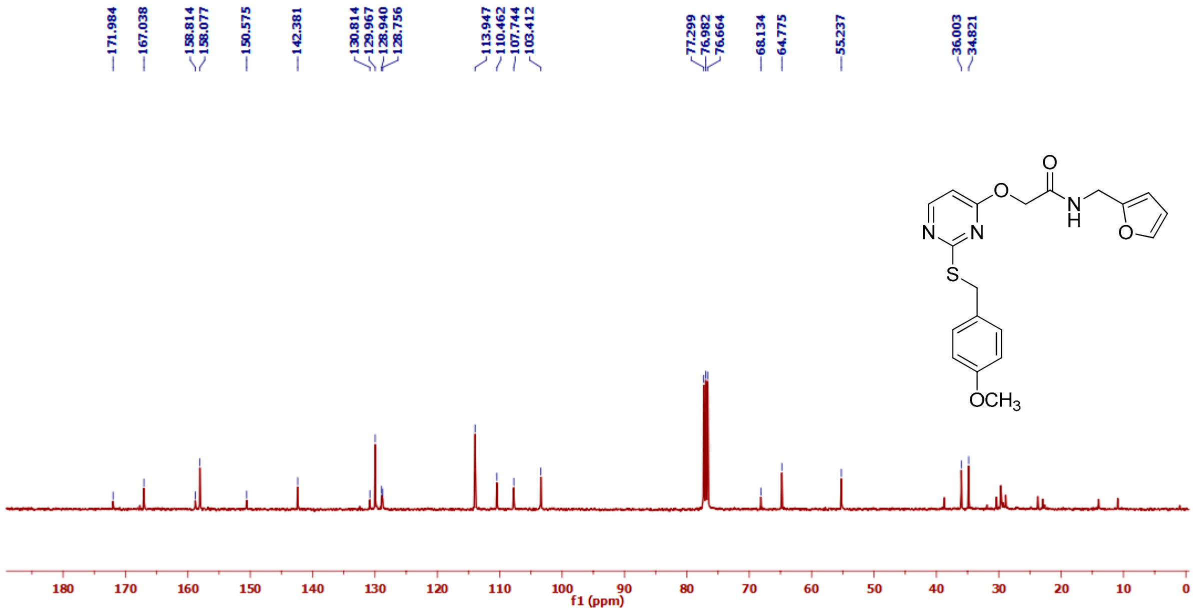

^{13}C NMR of compound **5h**

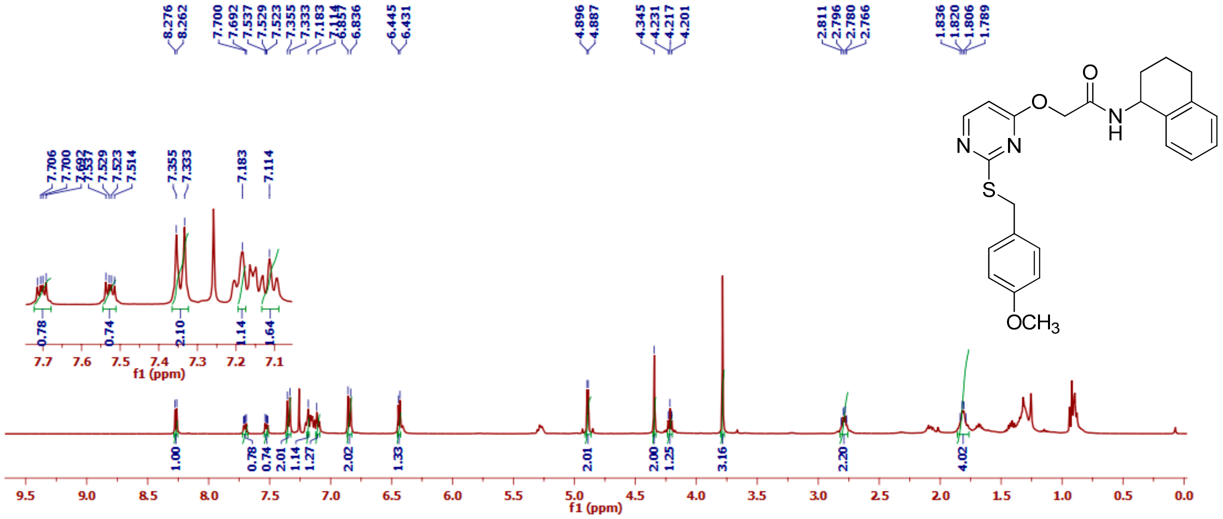

^{1}H NMR of compound **5i**

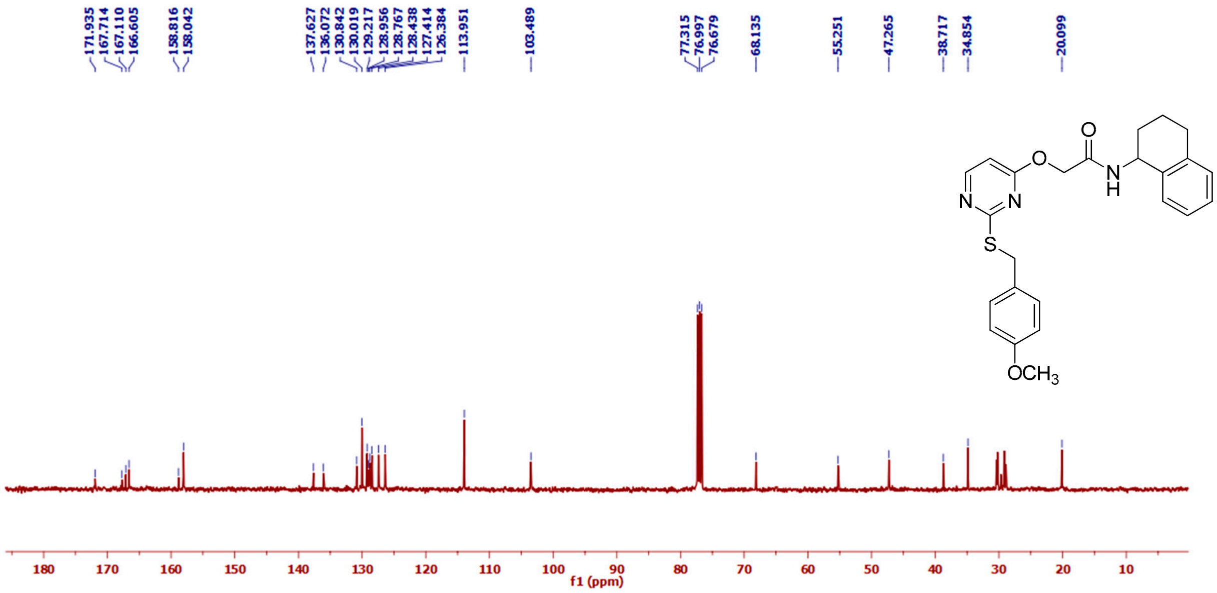

^{13}C NMR of compound **5i**

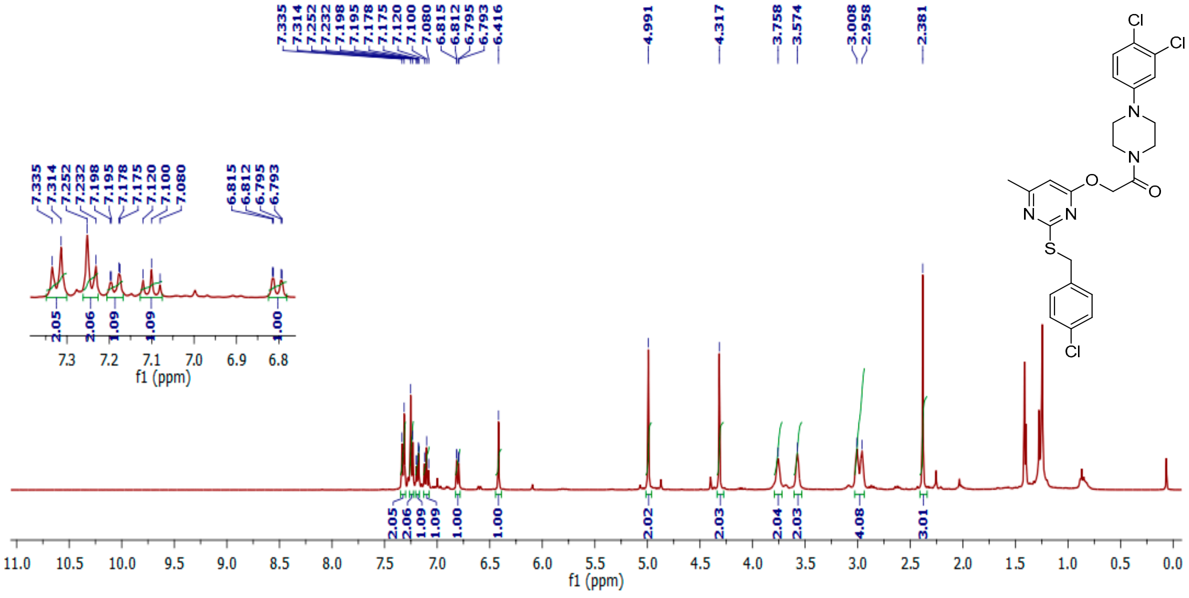

^{1}H NMR of compound **5j**

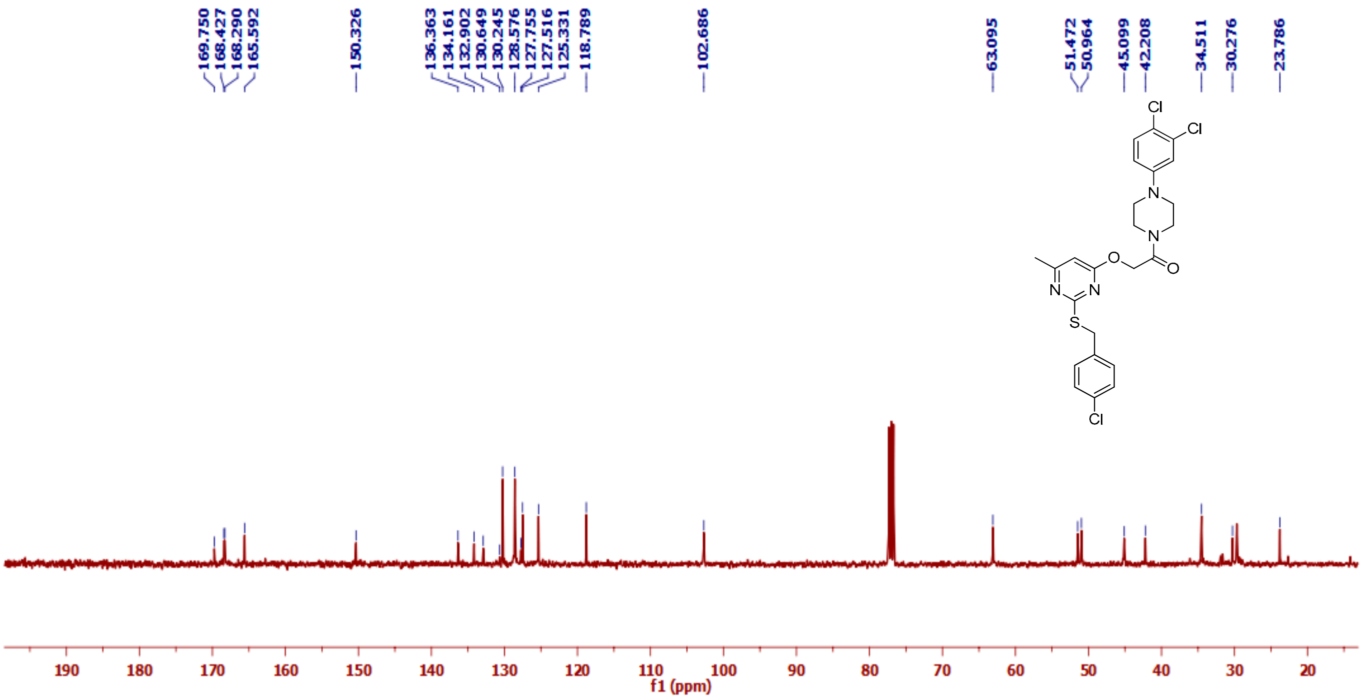

^{13}C NMR of compound **5j**

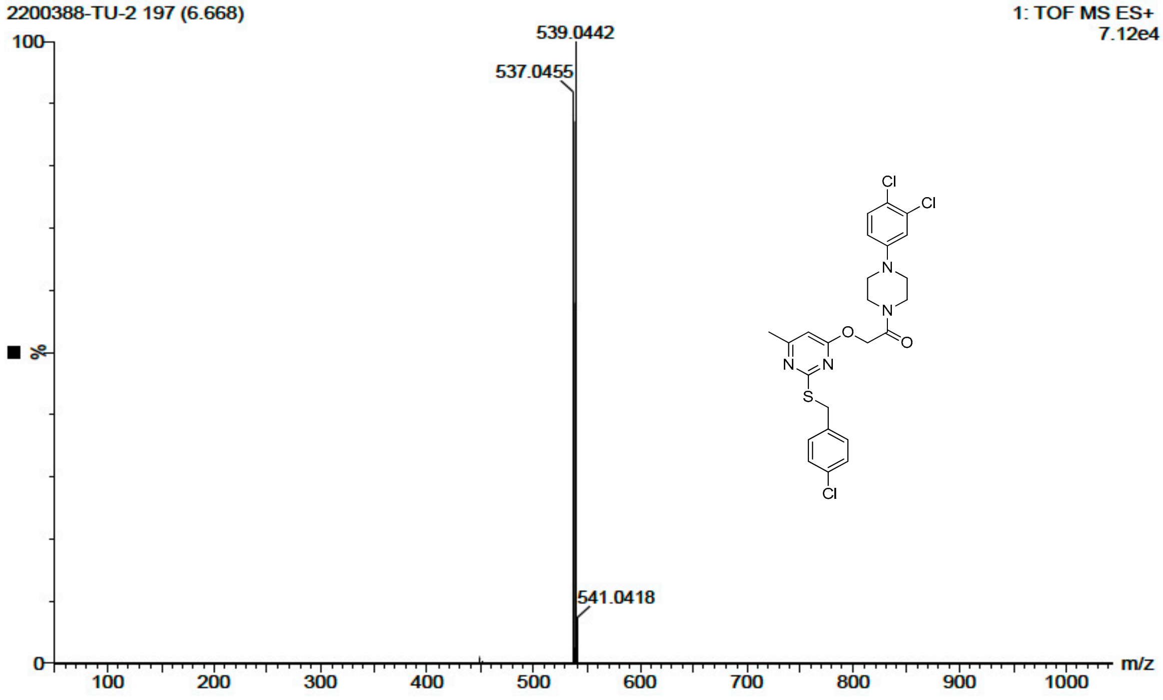

Mass spectra of 5**j**

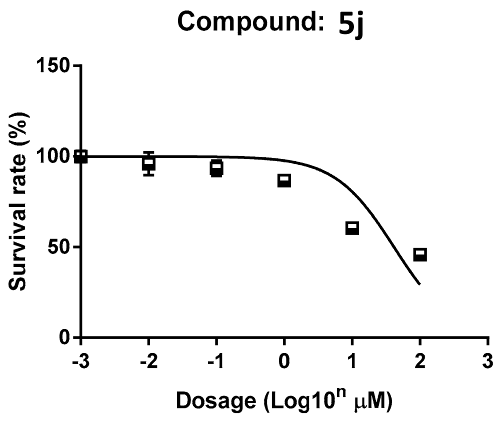

IC_{50} (μM) = 41.38
Log IC_{50} (μM) = 1.62

- **Cell line: MCF7 (2000 cells/per well[96])**
- **Treated time: 72hrs**
- **Assay: alamarBlue (4hrs incubated)**
- **Data: 5j**

Conc. (μM)	Viability	
	AVE.	± SD.
**0**	100.00	3.13
**0.01**	95.89	6.24
**0.1**	93.43	4.27
**1**	86.66	0.58
**10**	60.45	2.92
**100**	45.78	1.83

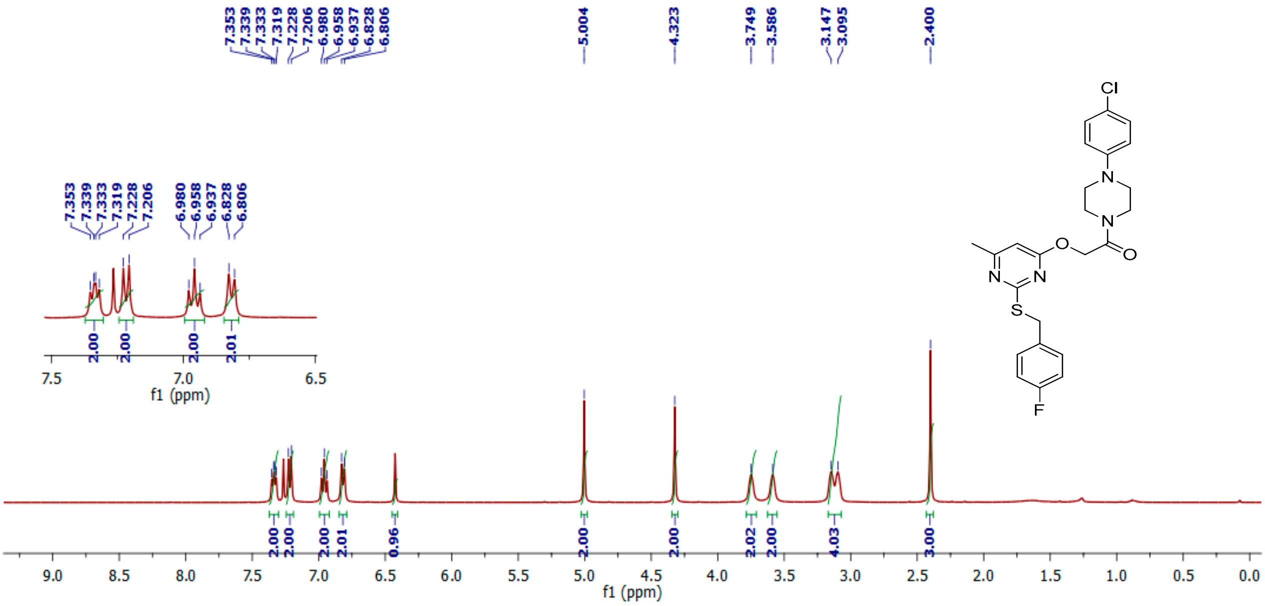

^{1}H NMR of compound **5k**

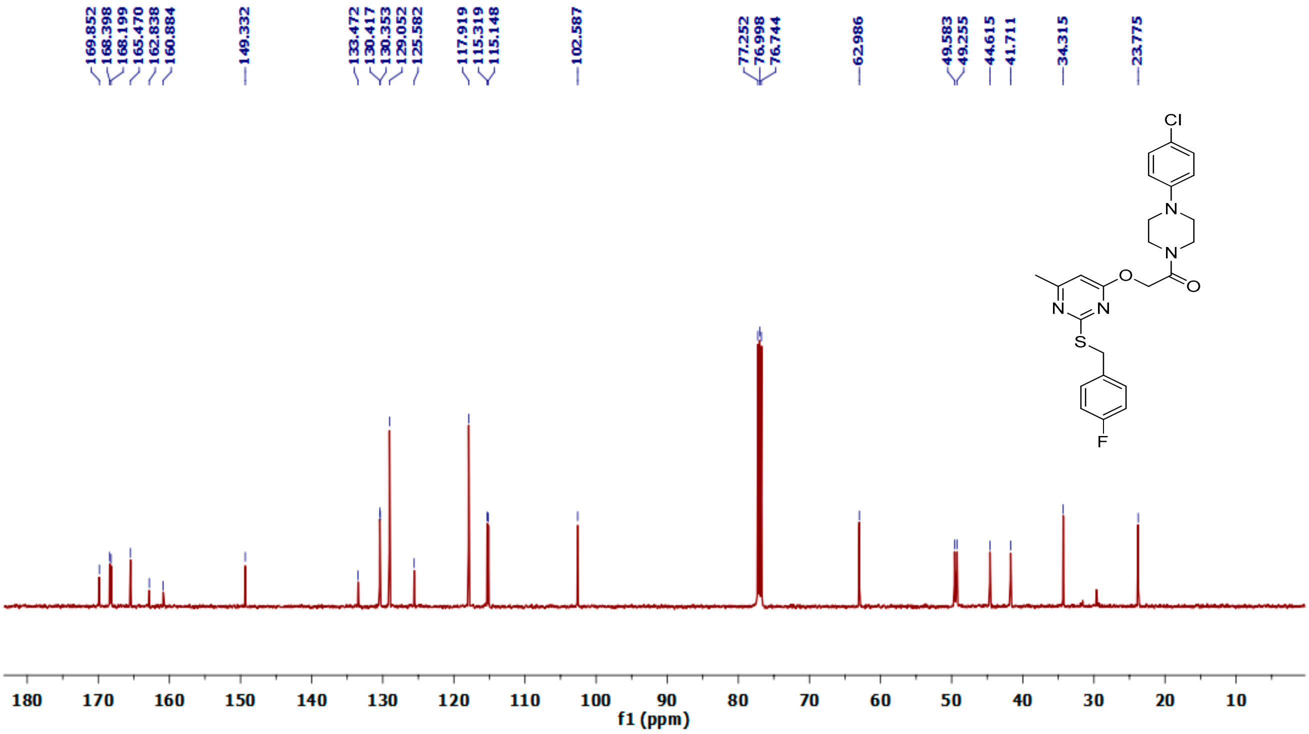

^{13}C NMR of compound **5k**

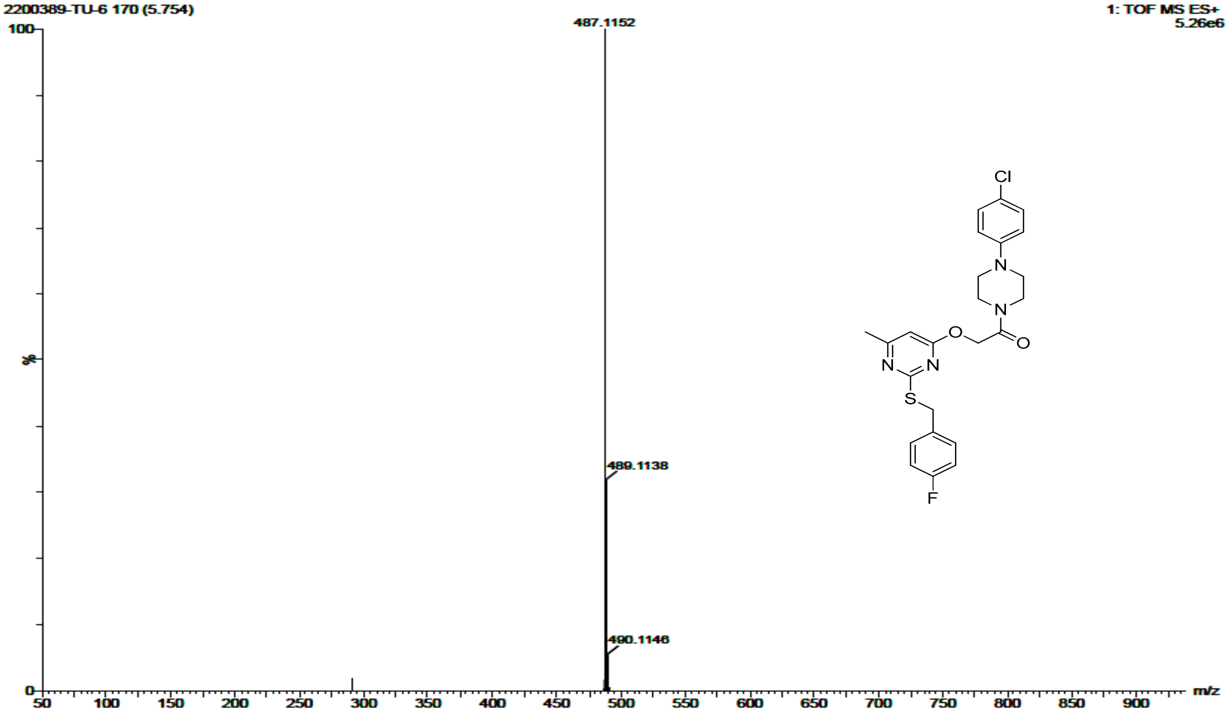

Mass spectra of **5k**

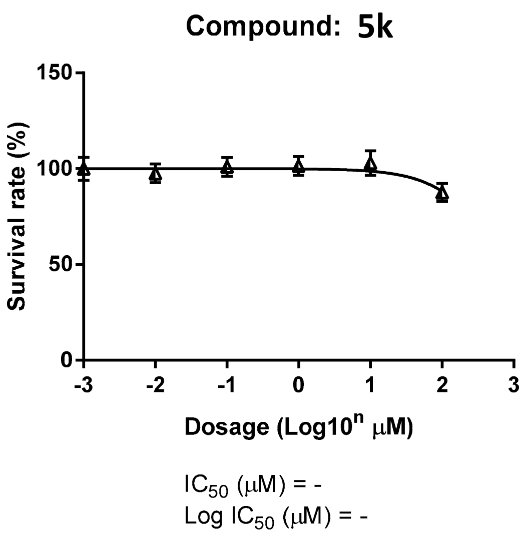

- Cell line: MCF7 (2000 cells/per well[96])
- Treated time: 72hrs
- Assay: alamarBlue (4hrs incubated)
- Data: 5k

Conc. (μM)	Viability	
	AVE.	± SD.
**0**	100.00	6.07
**0.01**	97.52	4.94
**0.1**	100.92	4.88
**1**	101.38	4.88
**10**	102.91	6.46
**100**	87.62	4.75

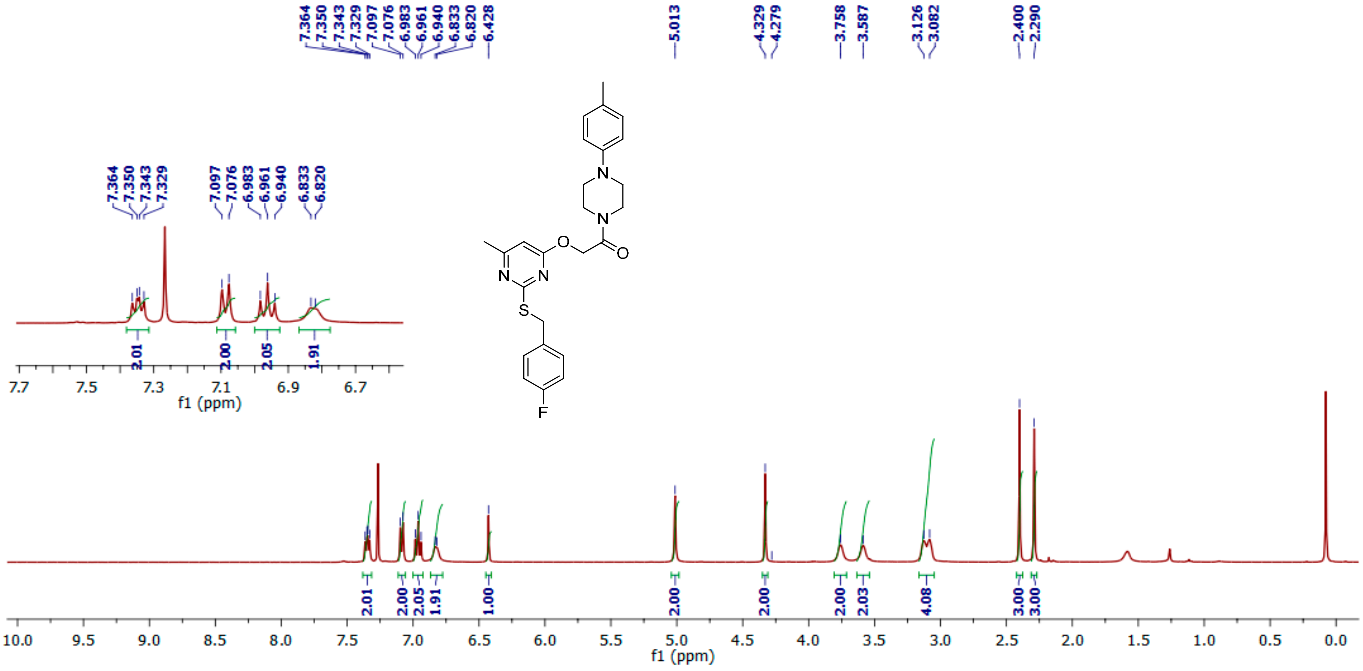

^{1}H NMR of compound **5l**

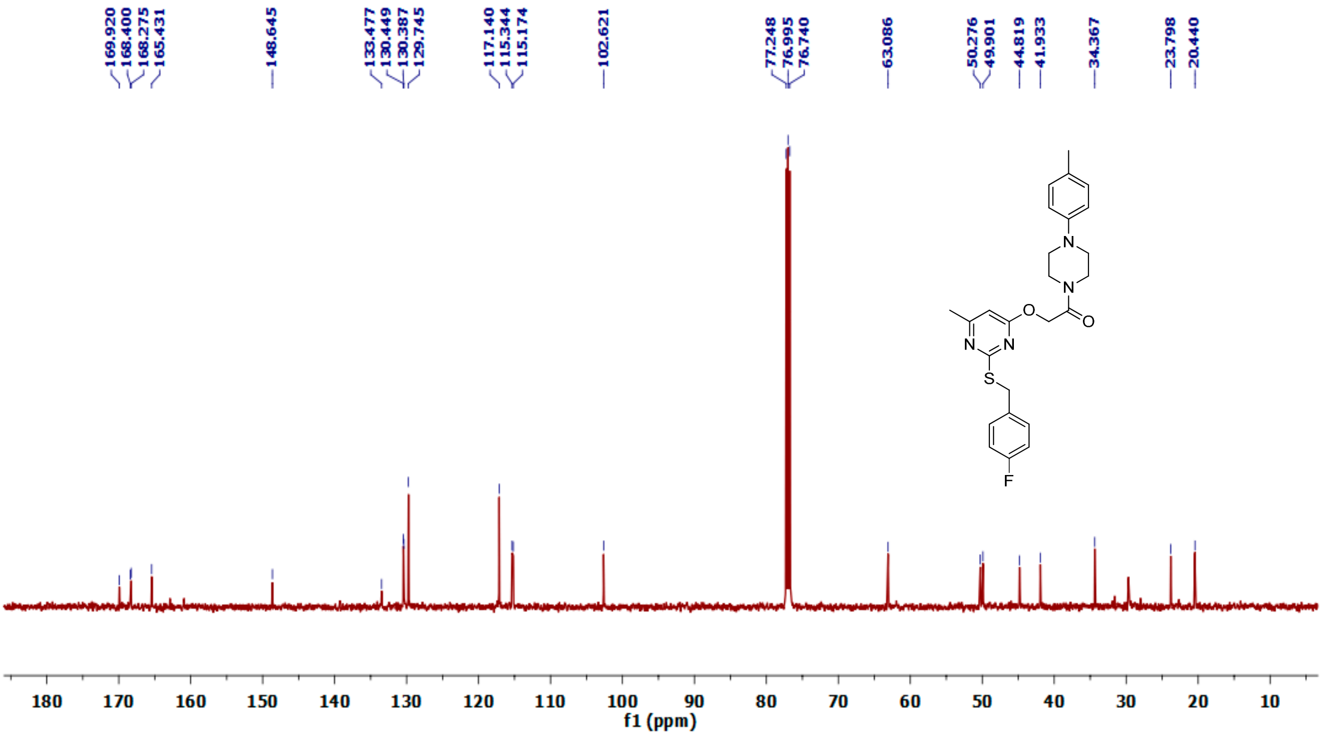

^{13}C NMR of compound **5l**

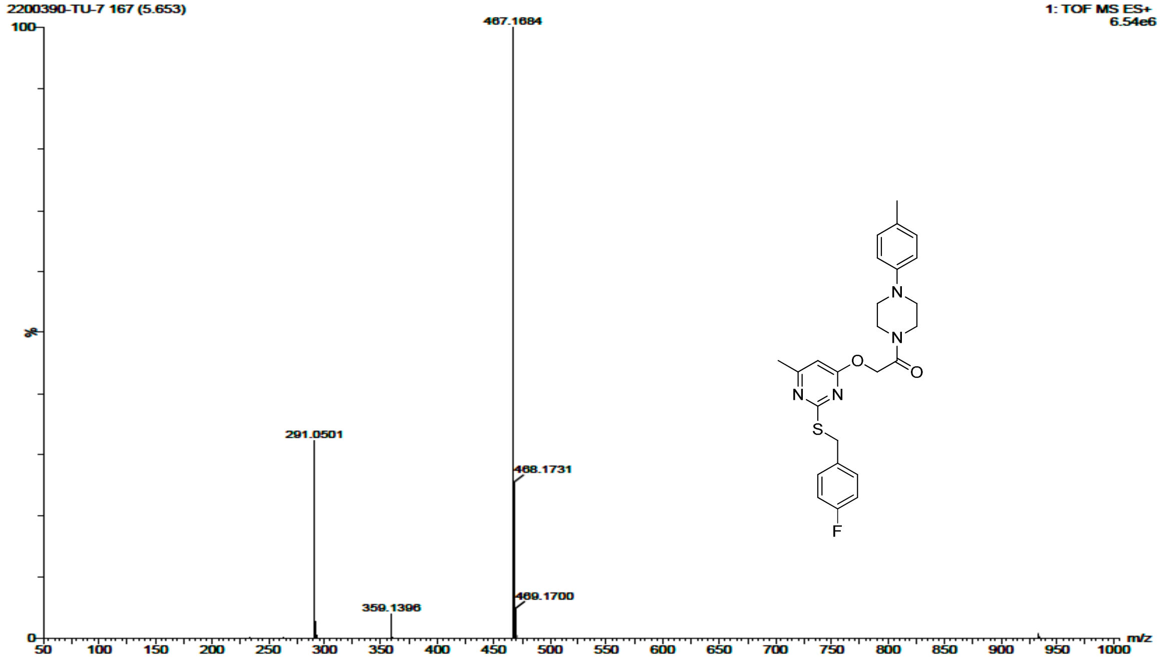

Mass spectra of **5l**

Compound: 5l

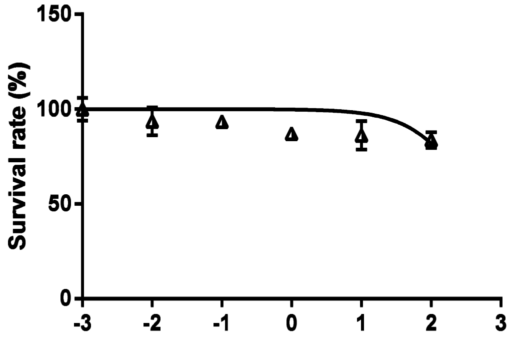

IC_{50} (μM) = -
Log IC_{50} (μM) = -

- **Cell line: MCF7 (2000 cells/per well[96])**
- **Treated time: 72hrs**
- **Assay: alamarBlue (4hrs incubated)**
- **Data: 5l**

Conc. (μM)	Viability	
	AVE.	± SD.
**0**	100.00	6.07
**0.01**	93.61	7.43
**0.1**	93.47	2.24
**1**	87.02	2.06
**10**	86.20	7.47
**100**	83.71	4.17

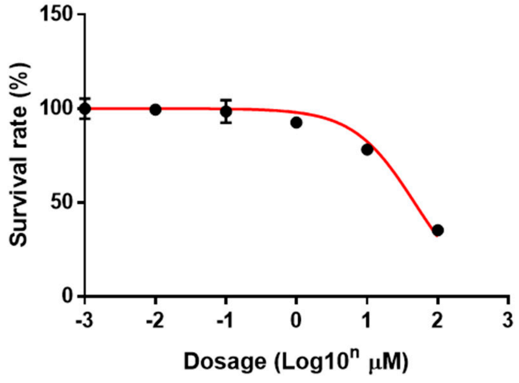

IC_{50} (μM) = 46.95
Log IC_{50} (μM) = 1.67

- **Assay:** alamarBlue
- **Cell line:** MCF-10A, 2000 cells/per well[96]
- **Compound: 5a**
- **Treatment:** 72hrs, 2% serum

**Conc. (μM)**	**Viability**	
	**AVE.**	**± SD.**
**0**	100.00	5.42
**0.01**	99.06	3.68
**0.1**	97.86	1.61
**1**	92.36	2.10
**10**	79.89	6.06
**100**	39.78	3.66

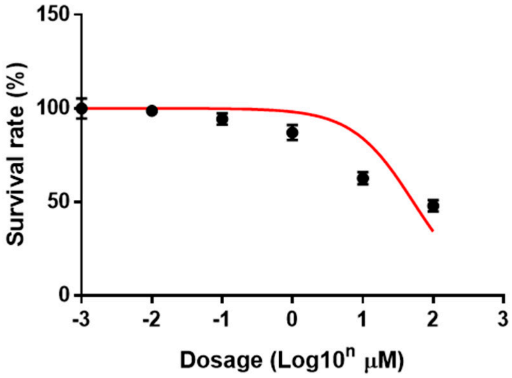

IC_{50} (μM) = 52.31
Log IC_{50} (μM) = 1.72

- **Assay:** alamarBlue
- **Cell line:** MCF-10A, 2000 cells/per well[96]
- **Compound: 5e**
- **Treatment:** 72hrs, 2% serum

Conc. (μM)	Viability	
	AVE.	± SD.
**0**	100.00	5.42
**0.01**	99.06	3.68
**0.1**	97.86	1.61
**1**	92.36	2.10
**10**	79.89	6.06
**100**	39.78	3.66

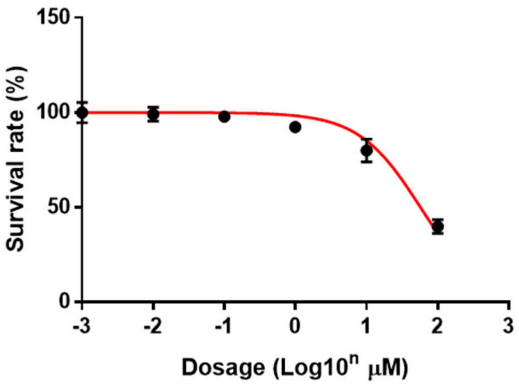

IC_{50} (μM) = 57.36
Log IC_{50} (μM) = 1.76

- **Assay:** alamarBlue
- **Cell line:** MCF-10A, 2000 cells/per well[96]
- **Compound: Olaparib**
- **Treatment:** 72hrs, 2% serum

Conc. (μM)	Viability	
	AVE.	±SD.
**0**	100.00	5.42
**0.01**	99.06	3.68
**0.1**	97.86	1.61
**1**	92.36	2.10
**10**	79.89	6.06
**100**	39.78	3.66
